# Targeting AKR1B1 inhibits metabolic reprogramming to reverse systemic therapy resistance in hepatocellular carcinoma

**DOI:** 10.1038/s41392-025-02321-9

**Published:** 2025-08-01

**Authors:** Qi Wang, Juan Liu, Ming Yang, Jun Zhou, Yaxuan Li, Jingjing Zheng, Hao Jia, Shuhua Yue, Yinpeng Le, Yuxin Su, Wenrui Ma, Ni An, Yunfang Wang, Jiahong Dong

**Affiliations:** 1 Hepato-Pancreato-Biliary Center, Beijing Tsinghua Changgung Hospital, Key Laboratory of Digital Intelligence Hepatology, Ministry of Education, School of Clinical Medicine, Tsinghua Medicine, Tsinghua University, Beijing, China; 2 Department of Hepatobiliary and Pancreatic Surgery, The First Hospital of Jilin University, Jilin University, Changchun, China; 3 Hubei Shizhen Laboratory, Wuhan, China; 4 Key Laboratory of Biomechanics and Mechanobiology (Beihang University), Ministry of Education, Institute of Medical Photonics, Beijing Advanced Innovation Center for Biomedical Engineering, School of Biological Science and Medical Engineering, Beihang University, Beijing, China; 5 Clinical Translational Science Center, Beijing Tsinghua Changgung Hospital, School of Clinical Medicine, Tsinghua Medicine, Tsinghua University, Beijing, China

**Keywords:** Cancer metabolism, Cancer therapy

## Abstract

Hepatocellular carcinoma (HCC) is a leading cause of cancer-related mortality, and resistance to systemic therapies remains a significant clinical challenge. This study investigated the mechanisms by which metabolic reprogramming contributes to systemic treatment resistance in HCC. We established HCC cell lines with multidrug resistance characteristics and observed enhanced metabolic activity in these cells. Integrated multiomics analyses revealed hyperactive glucose‒lipid and glutathione metabolic pathways that play critical roles in supporting tumor cell proliferation and survival. We constructed a metabolic reprogramming atlas for HCC-resistant cells and identified aldo-keto reductase (Aldo-keto reductase family 1 Member B1, AKR1B1) as a key regulator of this reprogramming, which sustains drug resistance by regulating energy metabolism and enhancing stress tolerance. Importantly, AKR1B1 expression levels are closely associated with drug resistance and poor prognosis in HCC patients. The secretory nature of AKR1B1 not only underscores its predictive value but also facilitates the intercellular transmission of drug resistance. In terms of overcoming resistance, the AKR1B1 inhibitor epalrestat significantly mitigated drug resistance when it was used in combination with standard therapies. These findings underscore the importance of metabolic reprogramming in the development of HCC resistance. AKR1B1, a key enzyme that regulates metabolic reprogramming, has been identified as a potential biomarker and therapeutic target, providing new insights into overcoming resistance in HCC treatment.

## Introduction

HCC, one of the leading causes of cancer-related mortality worldwide, is characterized by persistently high incidence and mortality rates.^1^ The majority of patients are diagnosed at advanced stages of the disease, necessitating systemic therapy as the primary treatment approach.^2–4^ However, the emergence of tumor resistance to therapeutic agents poses a significant clinical challenge, frequently leading to suboptimal therapeutic outcomes and unfavorable prognoses.^5^ As first-line tyrosine kinase inhibitors for advanced HCC, lenvatinib and sorafenib exhibit distinct receptor-targeting profiles despite their shared antiangiogenic properties. Lenvatinib potently inhibits VEGFR1–3, FGFR1–4, and PDGFRα,^6^ preferentially disrupting angiogenic signaling. In contrast, sorafenib broadly targets RAF kinase alongside VEGFR2/3 and PDGFRβ,^7^ enabling dual inhibition of proliferation and angiogenesis pathways. These differential selectivity patterns may contribute to the heterogeneous resistance mechanisms observed in clinical practice.^8,9^ Traditionally, the mechanisms underlying tumor resistance have been attributed to factors such as alterations in drug efflux pumps, dysregulation of intracellular signaling pathways, variations in drug metabolism, and enhanced DNA repair mechanisms.^10^ HCC develops heterogeneous resistance to lenvatinib and sorafenib through genetic, signaling, and microenvironmental adaptations. Mutation-driven resistance mechanisms include KRAS variants that sustain EGFR pathway activation, bypassing TKI targeting.^11^ Signaling plasticity is evidenced by EGFR-STAT3-ABCB1 axis reactivation in lenvatinib-resistant models^12^ and FGFR4-ERK pathway dependency in sorafenib-refractory cases.^13^ Noncoding RNAs further modulate resistance phenotypes, with circTTC13 enhancing sorafenib tolerance via SLC7A11 upregulation,^14^ whereas LINC01056 loss promotes compensatory survival signaling.^15^ Microenvironmental contributions span hypoxia-induced HIF-1α-mediated stemness^16,17^ to immune cell-mediated cytokine networks that sustain therapeutic tolerance.^18,19^ Notably, emerging evidence implicates exosomal cross-talk in the dissemination of resistance factors across tumor subclones.^20^ With the advent of single-cell transcriptomics, the ability to investigate resistance-related cellular heterogeneity at the single-cell level and analyze the genetic characteristics of resistant cells has provided unprecedented insights, facilitating a more precise elucidation of the mechanisms underlying therapeutic resistance.

In recent years, metabolic reprogramming in tumor cells has been identified as a novel mechanism contributing to therapeutic resistance, attracting considerable attention within the field. Tumor metabolic reprogramming refers to the adaptive process by which cancer cells alter their metabolic pathways to meet the heightened demands for energy and biosynthetic precursors, thereby facilitating cellular proliferation and promoting survival under adverse conditions.^21^ The concept of metabolic reprogramming traces back to the early 20th century, when Otto Warburg first reported that tumor cells exhibit an abnormally high rate of glycolysis even under aerobic conditions, a phenomenon now acknowledged as a hallmark of the extensive metabolic rewiring that defines cancer cells.^22^ This metabolic reprogramming not only perturbs conventional metabolic networks but also critically contributes to tumor cell proliferation, survival, immune evasion, and metastasis. With the advent of advanced multiomics technologies, researchers have increasingly focused on characterizing the intricate metabolic reprogramming landscape of tumor cells. Such efforts hold the promise of revealing the mechanisms underlying resistance and pinpointing key metabolic enzymes that shape therapeutic outcomes. In our previous review of studies on metabolic reprogramming in resistant HCC cells, we reported that, compared with their nonresistant counterparts, resistant HCC cells undergo a striking metabolic shift, which is characterized by increased energy storage, elevated energy production, and increased antioxidant capacity.^23^ However, to date, no study has comprehensively integrated metabolite- and enzyme-level analyses to construct a comprehensive metabolic reprogramming atlas of resistant HCC. Leveraging multiomics approaches provides a promising framework for identifying novel therapeutic targets, thereby paving the way for more effective treatment strategies against HCC.

Through an integrated approach combining transcriptomics, metabolomics, and metabolic flux analysis, we identified AKR1B1 as a key enzymatic target implicated in therapeutic resistance in HCC. AKR1B1, a rate-limiting enzyme in the polyol pathway, catalyzes the reduction of glucose to sorbitol and consequently modulates cellular metabolism and energy homeostasis.^24,25^ Previous studies have demonstrated that, in lung cancer cells, AKR1B1 can interact with STAT3 to regulate the expression of the cystine transporter SLC7A11, thereby promoting resistance to TKIs.^26^ For the first time, our study revealed that AKR1B1 is critically involved in mediating resistance in HCC by modulating metabolic reprogramming. However, its precise role in the context of precise HCC diagnosis and targeted therapy warrants further investigation.

This study aims to comprehensively investigate the metabolic reprogramming mechanisms underlying therapeutic resistance in HCC, with an emphasis on delineating the metabolic features linked to resistance. By constructing a detailed atlas of glucose and lipid metabolic reprogramming in resistant HCC cells, this work offers valuable insights into the metabolic adaptations that underpin resistance. Notably, our findings highlight AKR1B1 as a secreted protein with considerable potential as a biomarker for the early detection of HCC resistance. Furthermore, as a key metabolic enzyme influencing resistance, AKR1B1 has been identified as a promising therapeutic target for combination treatment strategies. Importantly, we demonstrated that the combined use of epalrestat, a clinically approved AKR1B1 inhibitor commonly used for the treatment of diabetic peripheral neuropathy, effectively mitigates Lenvatinib resistance. These findings not only offer a novel perspective for understanding the mechanisms of resistance in HCC but also lay a theoretical foundation for the development of novel predictive biomarkers and therapeutic approaches aimed at overcoming resistance in HCC.

## Results

### Metabolic alterations accompanying drug resistance in hepatocellular carcinoma

**Fig. 1:**
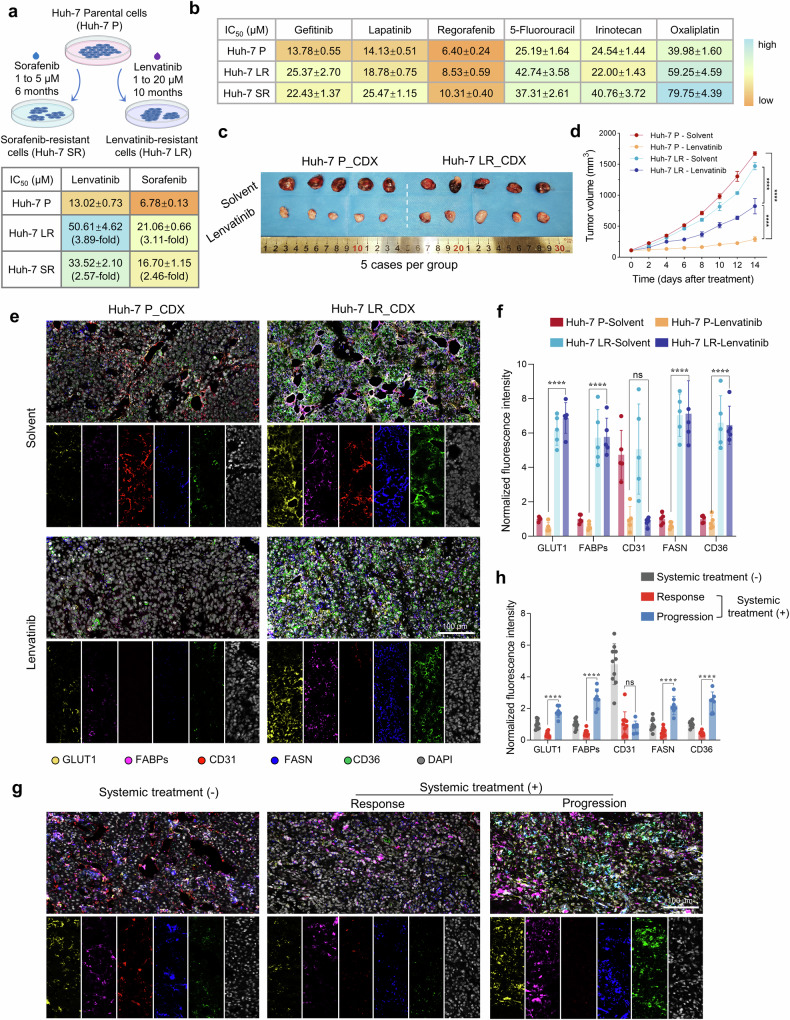
Multidrug resistance and associated metabolic adaptations in drug-resistant cells. **a** Schematic representation of the drug-resistant cell model development process alongside lenvatinib and sorafenib IC_50_ values. The drug concentrations used for induction ranged from 1–20 μM for the Huh-7 LR cells and from 1–5 μM for the Huh-7 SR cells. Schematic figures were generated with BioRender (https://app.biorender.com/). **b** Drug sensitivity testing for targeted therapies and chemotherapeutic agents in drug-resistant and parental cells, presented as IC_50_ values. **c** Drug sensitivity evaluation in CDX models derived from drug-resistant and parental cells (*n* = 5/group). CDX model drug application concentrations: solvent: 5‰ carboxymethyl cellulose sodium; lenvatinib: 5 mg/kg/d. **d** Tumor growth kinetics in CDX models derived from drug-resistant and parental cells. Testing method: Unpaired Student’s t-test. **e** Multicolor IF staining of metabolic enzymes in tumor tissues from CDX models of drug-resistant and parental cells. GLUT1 (yellow), FABPs (purple), CD31 (red), FASN (blue), CD36 (green), and DAPI (gray). Scale bar = 100 μm. **f** Quantification of multicolor IF staining intensity in tumor tissues from CDX models. Testing method: Unpaired Student’s t-test. **g** Multicolor IF staining of metabolic enzymes in tumor tissues from HCC patients treated with or without systemic therapy. GLUT1 (yellow), FABPs (purple), CD31 (red), FASN (blue), CD36 (green), and DAPI (gray). Scale bar = 100 μm. **h** Quantification of multicolor IF staining intensity in patient-derived tumor tissues. Testing method: Unpaired Student’s t-test. *p < 0.05, **p < 0.01, ***p < 0.001, ****p < 0.0001

The development of resistance to systemic therapies in HCC patients poses a significant barrier to extending patient survival. To elucidate the mechanisms of resistance and identify potential strategies for improvement, we established lenvatinib and sorafenib-resistant HCC cell lines, Huh-7 LR and Huh-7 SR, respectively, through a prolonged drug escalation method in Huh-7 cells (Fig. 1a and Supplementary Fig. 1a, b). Compared with the parental cell line (Huh-7 P), the resistant variants presented substantially greater IC_50_ values for lenvatinib and sorafenib. Specifically, the IC_50_ value for lenvatinib in Huh-7 LRs increased 3.89-fold, and the IC_50_ value for sorafenib in Huh-7 SRs increased 2.46-fold, indicating stable resistance characteristics (Fig. 1a and Supplementary Fig. 1c). Further analysis revealed cross-resistance between the two cell lines, in which Huh-7 LRs exhibited resistance to sorafenib and Huh-7 SRs displayed resistance to lenvatinib (Fig. 1a and Supplementary Fig. 1c). We also assessed the sensitivity of these resistant cell lines to other clinically relevant tyrosine kinase inhibitors (regorafenib, gefitinib, and lapatinib) and chemotherapeutic agents (5-fluorouracil, irinotecan, and oxaliplatin). The results indicated a general reduction in sensitivity to these drugs (Fig. 1b, Supplementary Fig. 2, and Table 1). In 3D cultured tumor spheroids, we observed that spheroids derived from resistant cells similarly exhibited resistance to both targeted and chemotherapeutic agents, with a lower rate of cell death (Supplementary Fig. 3). Moreover, compared with spheroids derived from Huh-7 P cells, those from resistant lines demonstrated enhanced tolerance to commonly used clinical treatment regimens for HCC patients (Supplementary Fig. 4).

**Table 1:** IC_50_ values and fold increase in drug resistance of parental vs. drug-resistant cells

Drugs	Huh-7 P	Huh-7 LR		Huh-7 SR	
	IC_50_ (μM)	IC_50_ (μM)	Resistant Index	IC_50_ (μM)	Resistant Index
Lenvatinib	13.02 ± 0.73	50.61 ± 4.62	3.89 ± 0.42	33.52 ± 2.10	2.57 ± 0.22
Sorafenib	6.78 ± 0.13	21.06 ± 0.66	3.11 ± 0.11	16.70 ± 1.15	2.46 ± 0.18
Regorafenib	6.40 ± 0.24	8.53 ± 0.59	1.33 ± 0.11	10.31 ± 0.40	1.61 ± 0.09
Gefitinib	13.78 ± 0.55	25.37 ± 2.70	1.84 ± 0.21	22.43 ± 1.37	1.63 ± 0.12
Lapatinib	14.13 ± 0.51	18.78 ± 0.75	1.32 ± 0.07	25.47 ± 1.15	1.80 ± 0.09
5-Fluorouracil	25.19 ± 1.64	42.74 ± 3.58	1.70 ± 0.18	37.31 ± 2.61	1.48 ± 0.13
Irinotecan	24.54 ± 1.44	22.00 ± 1.43	0.90 ± 0.08	40.76 ± 3.72	1.66 ± 0.18
Oxaliplatin	39.98 ± 1.60	59.25 ± 4.59	1.48 ± 0.13	79.75 ± 4.39	1.99 ± 0.12

When implanted into nude mice and subjected to lenvatinib intervention, tumors derived from resistant cells demonstrated heightened drug tolerance, characterized by increased tumor volumes and masses (Fig. 1c, d and Supplementary Fig. 5a, b). Consistent with the antiangiogenic properties of lenvatinib, quantitative CD31 IF demonstrated a significant reduction in microvascular density in treated parental tumors (78.87% decrease vs. solvent), whereas resistant tumors retained elevated vascularization (Fig. 1c and Supplementary Fig. 5c, d). This persistence of vascularization in resistant models aligns with their accelerated growth kinetics despite TKI administration. However, the volume of the Huh-7 LR-resistant cell-derived xenografts did not decrease, and the cells remained highly proliferative, indirectly suggesting the presence of alternative nutrient pathways supporting tumor growth (Supplementary Fig. 5e, f). Multicolor IF analysis revealed elevated expression of key enzymes associated with glucose and lipid metabolism (GLUT1 for glucose transport; FASN for fatty acid synthesis; CD36 and FABPs for fatty acid transport) in resistant tumor tissues compared with parental cells, which was unaffected by lenvatinib treatment, indicating metabolic reprogramming in resistant tumors (Fig. 1e, f). Furthermore, in tumor samples collected from certain HCC patients, metabolic enzymes related to glucose and lipid synthesis and transport were significantly upregulated in tissues from resistant patients compared with those from untreated patients and those from patients with a therapeutic response (Fig. 1g, h and Supplementary Table 1).

### Single-cell transcriptome sequencing highlights increased stemness and metabolic activity in drug-resistant cells

**Fig. 2:**
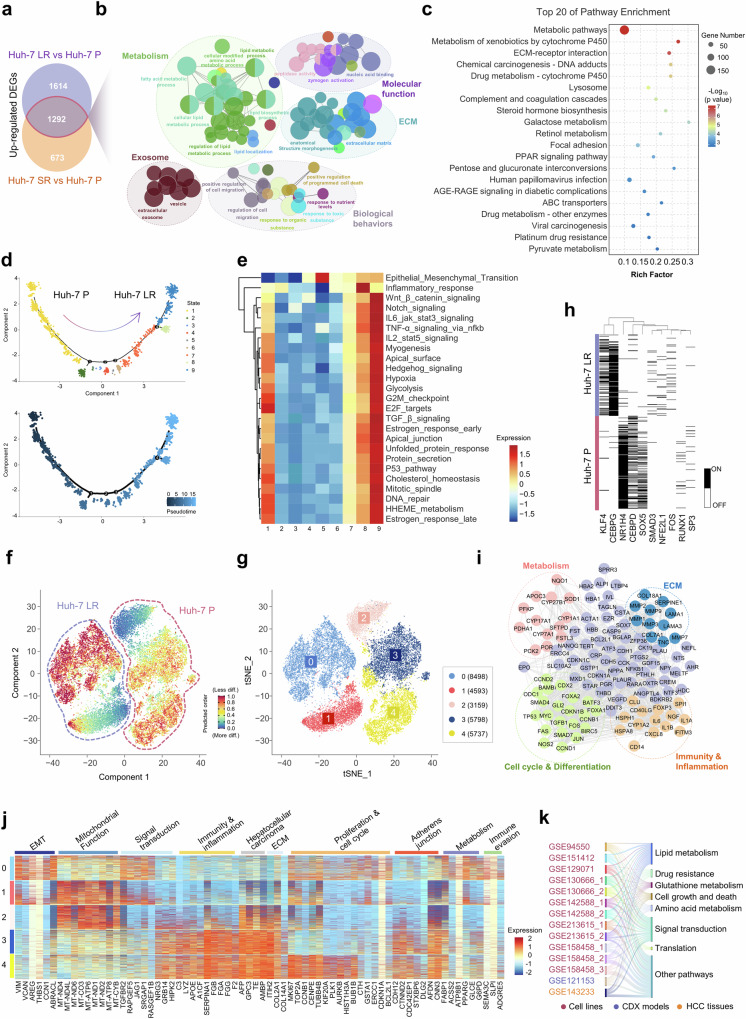
Transcriptomic analysis highlights stemness characteristics and metabolic reprogramming in drug-resistant cells. **a** Venn diagram depicting the upregulated genes shared between Huh-7 LR and Huh-7 SR (FC > 1.5, *p* < 0.05) based on bulk RNA sequencing. **b** PPI network analysis using ClueGo for commonly upregulated genes in the Huh-7 LR and Huh-7 SR. **c** KEGG analysis identified the top 20 enriched pathways of commonly upregulated genes in Huh-7 LR and Huh-7 SR cells. **d** Pseudotime trajectory analysis via Monocle2 and cell state clustering derived from single-cell sequencing of drug-resistant and parental cells. **e** Heatmap generated through GSVA via the MSigDB hallmark dataset for the 9 states derived from pseudotime analysis. **f** Stemness assessment of drug-resistant and parental cells from single-cell sequencing data conducted via CytoTRACE. **g** Clustering visualization through t-SNE dimensionality reduction from single-cell sequencing data. **h** Transcription factor analysis derived from single-cell sequencing of drug-resistant and parental cells. **i** PPI network analysis of downstream target gene clusters regulated by the transcription factors KLF4 and CEBPG. **j** GSEA was performed on clustering data from single-cell sequencing of drug-resistant and parental cells. **k** Proportional KEGG pathway enrichment analysis of upregulated genes across drug resistance-related datasets from multiple GEO database sources

To gain deeper insights into the molecular alterations associated with the acquisition of drug resistance in HCC cells, we performed an integrated analysis utilizing bulk RNA sequencing and single-cell transcriptomics. A total of 1,292 commonly upregulated genes were identified in two drug-resistant cell lines (Huh-7 LR and Huh-7 SR) (Fig. 2a, Supplementary Fig. 6a, and Supplementary Data 1–3). Gene Ontology (GO) biological process analysis revealed that these upregulated genes were enriched predominantly in pathways associated with cellular metabolism, exosome biogenesis, and extracellular matrix (ECM) organization (Fig. 2b). Further analysis via the Kyoto Encyclopedia of Genes and Genomes (KEGG) pathway database revealed significant enrichment in metabolic pathways, ECM‒receptor interactions, drug metabolism, lysosomal processes, ATP-binding cassette (ABC) transporters, and pyruvate metabolism in drug-resistant cells (Fig. 2c and Supplementary Data 4). Gene set enrichment analysis (GSEA) additionally demonstrated that fatty acid metabolism and epithelial‒mesenchymal transition (EMT)-related processes were markedly upregulated in drug-resistant cell lines compared with their parental counterparts (Supplementary Fig. 6b).

Single-cell transcriptomic sequencing revealed significant heterogeneity between drug-resistant cells and their parental counterparts. The t-distributed stochastic neighbor embedding (t-SNE) dimensionality reduction analysis clearly distinguished drug-resistant cell lines from drug-sensitive ones (Supplementary Fig. 7). Pseudotime trajectory analysis, performed via the Monocle2 algorithm, classified the cells into 9 distinct states, with states 1–3 corresponding to the parental Huh-7 P cells and states 4–9 representing the drug-resistant Huh-7 LR cells (Fig. 2d and Supplementary Fig. 8). GSVA of each state revealed a gradual increase in stemness-related regulation as cells transitioned from parental to drug-resistant states. In contrast, pathways related to metabolism and cell survival initially declined, followed by peak activity at state 9 (Fig. 2e). Furthermore, CytoTRACE analysis indicated that, overall, drug-resistant cells displayed significantly higher stemness levels than did parental cells (Supplementary Fig. 9). Both the resistant and parental populations exhibited notable intratumoral heterogeneity, consisting of subpopulations with differing levels of stemness (Fig. 2f).

To characterize the molecular distinctions between drug-resistant and parental cells further, we classified the resistant cells into two clusters (clusters 0 and 1) and the parental cells into three clusters (clusters 2, 3, and 4) (Fig. 2g). Transcription factor (TF) analysis revealed that KLF4, CEBPG, SMAD3, and FOS were upregulated in Huh-7 LR cells, whereas NR1H4, CEBPD, and SOX5 were downregulated (Fig. 2h). Further investigation demonstrated that the transcription factors KLF4 and CEBPG in resistant cells primarily modulate biological processes related to metabolism, the cell cycle and differentiation, immune and inflammatory responses, and ECM remodeling (Fig. 2i, Supplementary Fig. 10, and Supplementary Data 5). Notably, KLF4 has been demonstrated to induce cancer stem cell-like phenotypes in nonstem cancer cells,^27^ whereas CEBPG has been implicated in promoting disease progression across various cancers.^28–30^ Conversely, TFs silenced in resistant cells, such as CEBPD and NR1H4, play critical roles in promoting immune and inflammatory gene expression while enhancing tumor cell chemosensitivity^31–33^ (Supplementary Fig. 11 and Supplementary Data 6). GSEA of the identified clusters highlighted unique biological profiles. Cluster 0 (resistant cells) displayed the upregulation of genes associated with EMT, metabolism, the cell cycle, and immune evasion pathways. Clusters 1 (resistant cells) and 2 (parental cells) were associated with pathways related to mitochondrial function and signal transduction. Clusters 3 (parental cells) and 4 (parental cells) were associated with pathways involving adherens junctions, immune and inflammatory responses, HCC-specific signaling, and ECM organization (Fig. 2j and Supplementary Fig. 12). Additionally, a multidimensional pathway analysis was conducted using nine publicly available datasets, including drug-resistant cell lines, CDX (cell line-derived xenograft) models, and HCC tissues. This analysis revealed that genes upregulated in drug-resistant cells, compared with their parental counterparts, were associated primarily with lipid metabolism, drug resistance, glutathione metabolism, and signal transduction pathways (Fig. 2k and Supplementary Data 7).

### Metabolic reprogramming features of drug-resistant HCC cells and their association with resistance mechanisms

**Fig. 3:**
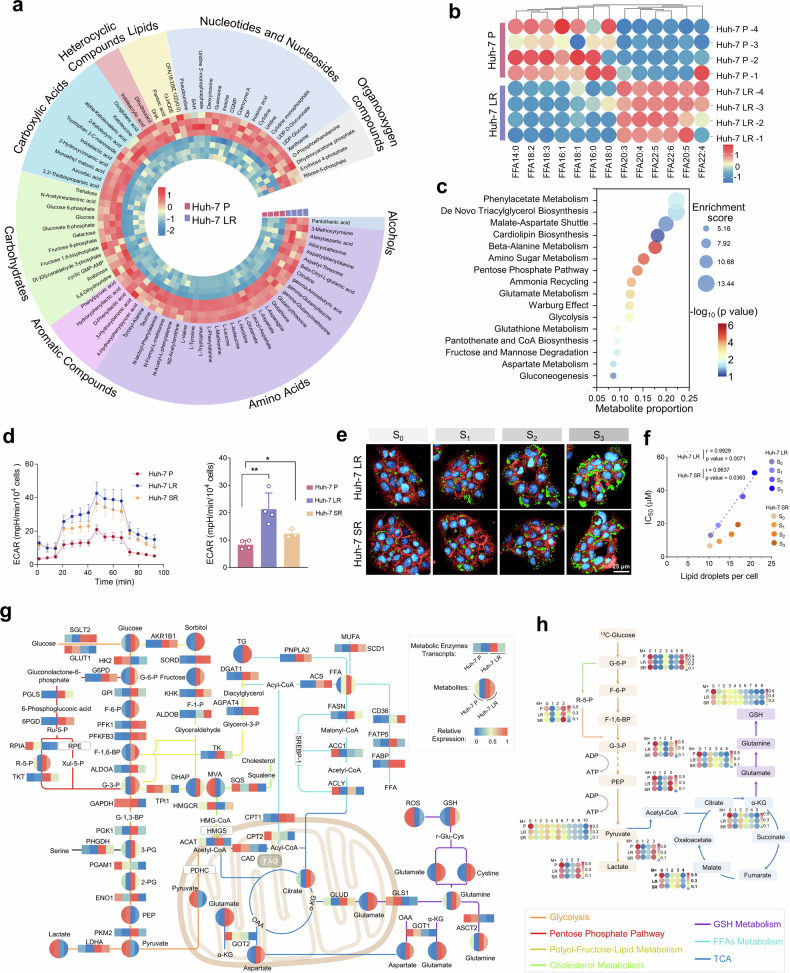
Metabolic adaptations in drug-resistant HCC cells. **a** Nontargeted metabolomics profiling of parental and Huh-7 LR cells. **b** FFA profiling in parental and Huh-7 LR cells. **c** Enrichment analysis of upregulated metabolites (FC > 1.2, *p* < 0.05) in Huh-7 LR cells compared with parental cells. **d** Seahorse extracellular flux analysis of glycolytic activity (ECAR) in parental and drug-resistant cells. Testing method: Unpaired Student’s t-test. **e** Visualization of intracellular lipid droplet accumulation during drug resistance development. Lipid droplets (green), the cell membrane (red), and the nucleus (blue). Scale bar = 25 μm. IC_50_ values: Huh-7 LR_lenvatinib (S_0_: 13.02 μM; S_1_: 19.15 μM; S_2_: 36.40 μM; S_3_: 50.61 μM); Huh-7 SR_sorafenib (S_0_: 6.77 μM; S_1_: 9.46 μM; S_2_: 13.53 μM; S_3_: 19.46 μM). f Correlation analysis between the intracellular lipid droplet content and drug IC_50_ in drug-resistant cells. Testing method: Pearson’s correlation coefficient test. **g** Integrated metabolomic (four replicates per cell type, averaged in pairs), transcriptomic (two replicates per cell type), and supplementary kit-based assay profiling to map metabolic adaptations in drug-resistant cells. **h** High-resolution ^13^C-glucose metabolic flux analysis of glycolysis, the TCA cycle, and glutathione metabolism pathways. The “M+number” indicates the number of additional ¹³C atoms in the metabolite molecule. *p < 0.05, **p < 0.01, ***p < 0.001, ****p < 0.0001

The metabolomic analysis revealed substantial variations across multiple metabolite categories, including heterocyclic compounds, lipids, nucleotides, and organic oxygen compounds, between the drug-resistant HCC cell line and its parental counterpart (Fig. 3a and Supplementary Data 8). Significant alterations in FFA metabolism, including carbon chain elongation and increased unsaturation, were evident in the drug-resistant cells (Fig. 3b and Supplementary Data 9). Pathway enrichment analysis of significantly upregulated metabolites in drug-resistant cells revealed notable alterations in pathways, including β-alanine metabolism, the Warburg effect, glutamine metabolism, de novo triglyceride synthesis, and glutathione metabolism, all of which are pivotal for cellular growth, energy production, and substrate availability (Fig. 3c and Supplementary Data 10). This study corroborated the phenomenon of aerobic glycolysis, as first described by Otto Warburg, whereby tumor cells produce high levels of lactate even under aerobic conditions, using a Seahorse extracellular flux analyzer. Compared with their parental counterparts, drug-resistant cells presented significantly increased glycolytic activity, glycolytic capacity, and glycolytic reserve, emphasizing the centrality of glycolysis as a primary energy source (Fig. 3d and Supplementary Fig. 13). The substantial increase in the levels of NAD^+^, a primary hydrogen acceptor involved in both glycolysis and the TCA cycle, further indicated an increased metabolic state of glycolysis and increased TCA cycle activity in the drug-resistant cells. Moreover, the observed increases in NADP^+^ and NADPH levels, which are linked to the pentose phosphate pathway, biosynthesis, biotransformation, and antioxidant responses, suggest enhanced energy metabolism and biosynthetic capacity in drug-resistant cells (Supplementary Fig. 14).

In the evaluation of energy storage metabolites, the triglyceride content in drug-resistant cells was significantly greater than that in parental cells, which aligns with lipid content assessments in tumor tissues (Supplementary Fig. 15a). Analysis of the lipid droplet size, number, and distribution via the neutral lipid dye Nile Red revealed significant increases in both the quantity and size of lipid droplets in drug-resistant cells compared with those in parental cells (Supplementary Fig. 15b–e). Raman spectroscopy (Raman shift: 2850 cm⁻¹), which is utilized for lipid molecule identification, further confirmed the elevated lipid content in the drug-resistant cells (Supplementary Fig. 16). Stimulated Raman scattering (SRS) vibrational imaging with a deuterium-labeled palmitic acid (D_31_-PA) probe was employed to observe lipid uptake, revealing a significantly stronger CD_L_ channel signal (Raman shift: 2107 cm⁻¹) in drug-resistant cells than in parental cells (Supplementary Fig. 17). Real-time quantification of FFA uptake, conducted with an FFA tracer and high-content fluorescence imaging, corroborated the findings from SRS imaging, which revealed markedly increased fatty acid uptake in drug-resistant cells (Supplementary Fig. 18 and Supplementary Data 11). Additionally, the level of FFA β-oxidation (FAO) was significantly elevated in drug-resistant cells, indicating enhanced metabolic activity that supports survival and proliferation under drug-induced stress (Supplementary Fig. 19). Importantly, the IC_50_ values of lenvatinib and sorafenib enabled us to define four resistance stages (S_0_, S_1_, S_2_, and S_3_) during the establishment of the drug-resistant cell model. The quantification of lipid droplet accumulation at each stage revealed a progressive increase in lipid droplet abundance corresponding to the development of resistance. (Fig. 3e and Supplementary Fig. 20). A strong correlation was observed between the degree of resistance and the number of lipid droplets (Fig. 3f). Furthermore, exposure of parental cells to palmitic acid (PA) demonstrated that increased lipid droplet accumulation facilitates both cell proliferation and drug resistance. These findings highlight the pivotal role of lipid droplet accumulation in the progression of drug resistance (Supplementary Fig. 21).

By integrating transcriptomic data, which reflect the expression of metabolic enzymes, with metabolomic data, which capture metabolite levels, a comprehensive metabolic reprogramming map associated with hepatocellular carcinoma drug resistance was constructed. This integrative analysis revealed a high degree of concordance between the regulation of metabolic enzymes and metabolites in drug-resistant cells, with glucose, lipid, and amino acid metabolic pathways demonstrating significantly elevated activity (Fig. 3g and Supplementary Data 2, 8). Metabolic flux analysis via ^13^C-labeled glucose further revealed significantly increased activity across several critical metabolic pathways, including glutathione metabolism, the TCA cycle, glycolysis, the pentose phosphate pathway, and the hexosamine biosynthetic pathway (Fig. 3h, Supplementary Fig. 22–24, and Supplementary Data 12). These findings highlight the pivotal role of these metabolic pathways in meeting the elevated energy demands and supporting the survival of drug-resistant cells.

### Knocking down AKR1B1 enhances drug sensitivity in drug-resistant hepatocellular carcinoma cells

**Fig. 4:**
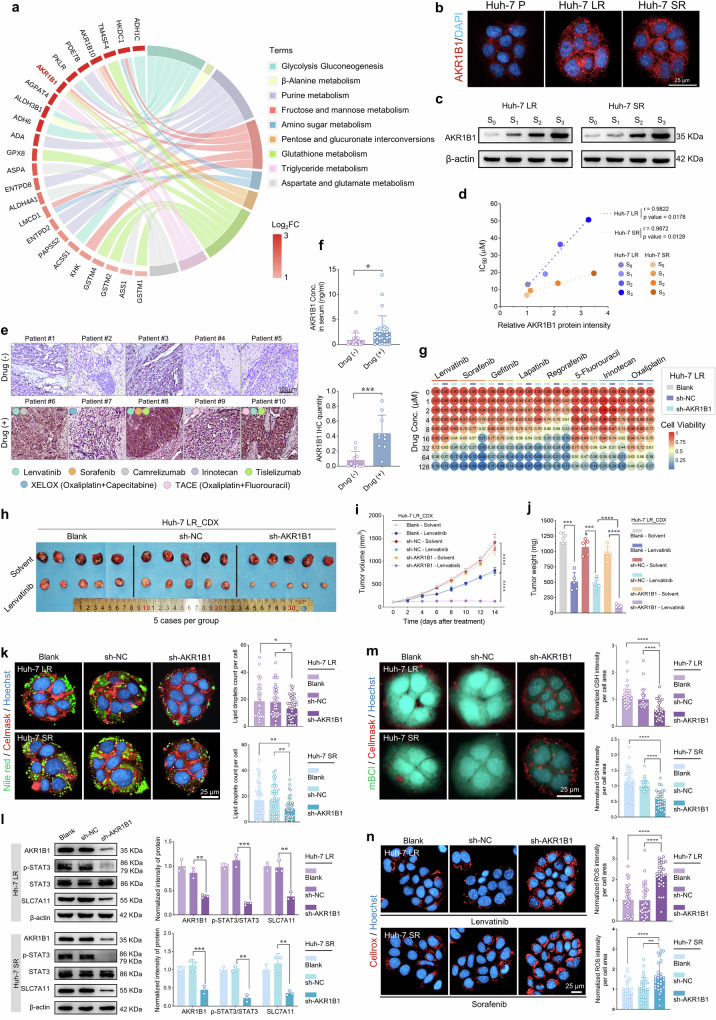
AKR1B1 overexpression in drug-resistant cells modulates drug sensitivity. **a** Integrated transcriptomic (upregulated genes, FC > 2, *p* < 0.05) and metabolomic profiling identified the key enzymes implicated in HCC drug resistance. **b** IF validation of AKR1B1 expression in parental and drug-resistant cells. AKR1B1 (red) and DAPI (blue). Scale bar = 25 μm. **c** WB analysis of AKR1B1 expression in cells during the development of drug resistance. **d** Correlation analysis of AKR1B1 expression levels with drug resistance in drug-resistant cells. Testing method: Pearson’s correlation coefficient test. **e** IHC staining of AKR1B1 in tumor tissues from HCC patients treated with or without systemic therapy. The results of the IHC quantification of AKR1B1 are presented in the right panel. Scale bar = 100 μm. Testing method: Unpaired Student’s t-test. **f** Quantification of serum AKR1B1 levels in HCC patients treated with or without systemic therapy. Testing method: Unpaired Student’s t-test. **g** Heatmap illustrating drug sensitivity in Huh-7 LR cells following AKR1B1 knockdown. **h** Drug sensitivity evaluation in CDX models derived from drug-resistant cells following AKR1B1 knockdown (*n* = 5/group). CDX model drug application concentrations: solvent: 5‰ carboxymethyl cellulose sodium; lenvatinib: 5 mg/kg/d. **i** Tumor growth kinetics in CDX models generated from drug-resistant cells following AKR1B1 knockdown. Testing method: Unpaired Student’s t-test. **j** Tumor weight measurements in CDX models generated from drug-resistant cells following AKR1B1 knockdown. Testing method: Unpaired Student’s t-test. **k** Visualization of intracellular lipid droplet levels in drug-resistant cells following AKR1B1 knockdown. Lipid droplets (green), the cell membrane (red), and the nucleus (blue). Scale bar = 25 μm. Testing method: Unpaired Student’s t-test. **l** WB analysis of key glutathione-regulating enzymes in drug-resistant cells following AKR1B1 knockdown via statistical analysis. Testing method: Unpaired Student’s t-test. **m** Quantification of intracellular GSH levels in drug-resistant cells following AKR1B1 knockdown. GSH (teal green), the cell membrane (red), and the nucleus (blue). Scale bar = 25 μm. Testing method: Unpaired Student’s t-test. **n** Quantification of ROS levels in drug-resistant cells following AKR1B1 knockdown. ROS (red), nuclei (blue). Lenvatinib: 50.61 μM (IC_50_), 12 h; sorafenib: 16.70 μM (IC_50_), 12 h. Scale bar = 25 μm. Testing method: Unpaired Student’s t-test. *p < 0.05, **p < 0.01, ***p < 0.001, ****p < 0.0001

Through metabolomic analysis, metabolic pathways significantly enriched during the progression of drug resistance in HCC were identified. Key rate-limiting enzymes upregulated within these pathways were further identified through the integration of transcriptomic data. As shown in Fig. 4a, AKR1B1 was identified as a prominently upregulated rate-limiting enzyme closely associated with fructose metabolism, the pentose phosphate pathway, glutathione metabolism, and triglyceride metabolism (Supplementary Data 13). Previous studies have established the critical role of the fructose metabolism pathway in tumor progression.^34,35^ To elucidate the underlying mechanisms, key enzymes involved in the polyol‒fructose‒lipid metabolic axis, including AKR1B1, sorbitol dehydrogenase (SORD), ketohexokinase (KHK), aldolase B (ALDOB), α-glucosidase 14 (AGPA14), and diacylglycerol O-acyltransferase 1 (DGAT1), were analyzed (Supplementary Fig. 25a). Notably, the majority of these enzymes were progressively upregulated during acquired resistance (Supplementary Fig. 25b). ALDOB, while included for completeness, presented baseline expression levels below reliable detection thresholds (FPKM < 1.0) across all the cell models. The silencing of these rate-limiting enzymes universally enhanced drug sensitivity, with AKR1B1, the most upstream enzyme in this pathway, demonstrating the most pronounced effect (Supplementary Fig. 25c, d).

As shown in Fig. 4b and Supplementary Fig. 26, AKR1B1 protein expression levels were significantly elevated in drug-resistant cells compared with their parental counterparts, with a progressive increase observed during the development of resistance (Fig. 4c). Notably, AKR1B1 expression was strongly positively correlated with the IC_50_ values of lenvatinib and sorafenib in Huh-7 LR and Huh-7 SR cells (Fig. 4d). To further validate these findings, tissue and blood samples from HCC patients who had undergone systemic therapy for more than 3 months and those without such treatment were analyzed. IHC and IF staining revealed substantially greater AKR1B1 expression in the treated group than in the untreated group (Fig. 4e and Supplementary Fig. 27). Moreover, the serum levels of AKR1B1 were markedly elevated in patients receiving sustained treatment compared with those in untreated individuals (Fig. 4f and Supplementary Table 2). These results suggest that AKR1B1 is upregulated during drug therapy, thereby mediating cellular survival under therapeutic stress conditions, indicating its potential utility as a biomarker for predicting therapeutic resistance in HCC patients.

Silencing AKR1B1 in drug-resistant cells substantially improved their sensitivity to the therapeutic agents responsible for inducing resistance (Supplementary Fig. 28). Heatmap analysis of multidrug resistance further demonstrated that AKR1B1 silencing significantly increased cellular sensitivity across a spectrum of targeted therapies and chemotherapeutic agents, including regorafenib, gefitinib, lapatinib, irinotecan, 5-fluorouracil, and oxaliplatin, underscoring AKR1B1’s pivotal role in mediating drug resistance phenotypes (Fig. 4g, Supplementary Fig. 29, and Supplementary Table 3). To further investigate the functional implications of AKR1B1 in vivo, tumor growth assays were performed in a nude mouse model. Compared with control tumors, tumors derived from AKR1B1-silenced cells presented significantly reduced volumes, slower growth rates, and lower tumor masses (Fig. 4h–j and Supplementary Fig. 30a). Additionally, Lenvatinib-treated tumors presented reduced vascular density, as indicated by Ki-67 staining, indicating pronounced suppression of tumor proliferation in the AKR1B1-silenced group (Supplementary Fig. 30b–d). Even in untreated tumors, proliferation was moderately inhibited, although to a comparatively lesser extent. Collectively, these findings highlight AKR1B1 as a compelling therapeutic target for enhancing the sensitivity of HCC cells to conventional treatments.

### AKR1B1 enhances drug resistance in HCC cells via multiple pathways

To determine the established role of AKR1B1 in cellular drug resistance, this study further investigated the impact of altered AKR1B1 expression on the metabolic phenotype of resistant cells. Figure 4k and Supplementary Fig. 31 revealed that silencing of the AKR1B1 gene led to a marked reduction in lipid droplet accumulation and triglyceride content within resistant cells, accompanied by a significant decrease in FAO levels. Integrated transcriptomic and metabolomic profiling revealed coordinated hyperactivation of glutathione metabolism, glycolysis, and polyol/fructose metabolic pathways during acquired resistance (Fig. 3c, g, h). Among these pathways, the glutathione pathway has been shown to play a pivotal role in maintaining cell survival. Research has demonstrated that AKR1B1 modulates cystine uptake and glutathione synthesis flux in lung cancer cells via the STAT3/SLC7A11 signaling axis.^26^ Consistently, this study revealed similar patterns in drug-resistant HCC cells, where AKR1B1 knockdown significantly suppressed STAT3 phosphorylation and SLC7A11 protein expression (Fig. 4l). To further elucidate the role of AKR1B1 in regulating the glutathione regulatory pathway in drug-resistant HCC cells, the baseline activity of this pathway was assessed. The results revealed that the glutathione regulatory pathway was significantly upregulated in resistant cells compared with parental cells (Supplementary Fig. 32a). The quantification of glutathione levels further confirmed that compared with parental cells, drug-resistant cells presented significantly increased glutathione levels (Supplementary Fig. 32b). Concurrently, measurement of ROS levels revealed that parental cells exhibited increased sensitivity to drug treatment with increased ROS accumulation (Supplementary Fig. 32c). These findings suggest that resistant cells utilize a robust self-protective mechanism involving increased glutathione levels and decreased ROS accumulation, both of which contribute to increased cell survival. However, upon AKR1B1 knockdown, this self-protective adaptation was effectively disrupted. The glutathione regulatory pathway was significantly suppressed, as evidenced by the suppressed expression of key regulatory proteins, leading to a decrease in intracellular glutathione levels and a pronounced increase in ROS levels following drug treatment (Fig. 4l–n). Furthermore, in vivo experiments validated the in vitro findings and further confirmed that AKR1B1 plays a pivotal role in the glutathione regulatory pathway in drug-resistant HCC cells (Supplementary Fig. 33). These results highlight the pivotal regulatory role of AKR1B1 in sustaining the metabolic adaptations that underlie chemoresistance in HCC.

To further investigate the mechanisms underlying the upregulation of AKR1B1 in response to drug-induced selective pressure, we employed multiple predictive tools, including Cistrome, TCGA_LIHC, hTFtarget, and ENCODE, to identify potential transcription factors regulating AKR1B1 expression. By integrating these predictions with RNA-seq data from drug-resistant cells, we identified FOSL2, a member of the FOS transcription factor family, as a key regulator of AKR1B1 expression (Supplementary Fig. 34). This finding aligns with the results from single-cell transcriptomic sequencing (Fig. 2h). Previous studies have reported that FOSL2 can indirectly promote angiogenesis in tumor tissues, even when classical VEGF signaling is inhibited by anti-VEGF antibodies or axitinib.^36^ These findings suggest that FOSL2 may be adaptively upregulated in response to antiangiogenic therapies such as lenvatinib and sorafenib. In support of this hypothesis, our data demonstrated that FOSL2 expression was markedly elevated in drug-resistant cells compared with their parental counterparts. Additionally, GO enrichment analysis revealed significant upregulation of the Wnt signaling pathway, which may have contributed to the observed increase in FOSL2 expression (Supplementary Fig. 35a). We observed significant nuclear accumulation of nonphospho (active) β-catenin, a critical protein in the Wnt pathway, in drug-resistant cells (Supplementary Fig. 35b). Furthermore, the Wnt signaling inhibitor XAV9393 downregulated FOSL2 expression, underscoring the regulatory role of the Wnt pathway in modulating FOSL2 expression (Supplementary Fig. 35c). This observation is consistent with previous reports, which demonstrated the role of the Wnt pathway in promoting TKI resistance in liver cancer cells^37–39^ and enhancing FOSL2 expression in drug-resistant colorectal carcinoma cells.^40^ Through analysis via the JASPAR database,^41^ a potential binding motif for FOSL2 was predicted on the AKR1B1 promoter region: “CAGTGACTCAT” (Supplementary Fig. 36a, b). Furthermore, analysis of downstream target genes potentially regulated by FOSL2 via GO enrichment analysis revealed that these genes were associated primarily with the regulation of cellular metabolic processes (Supplementary Fig. 36c, d). These findings suggest a critical role for FOSL2 in driving the adaptive upregulation of AKR1B1 and orchestrating broader metabolic reprogramming in drug-resistant cancer cells.

### AKR1B1 mediates the transmission of drug resistance and correlates with poor prognosis

**Fig. 5:**
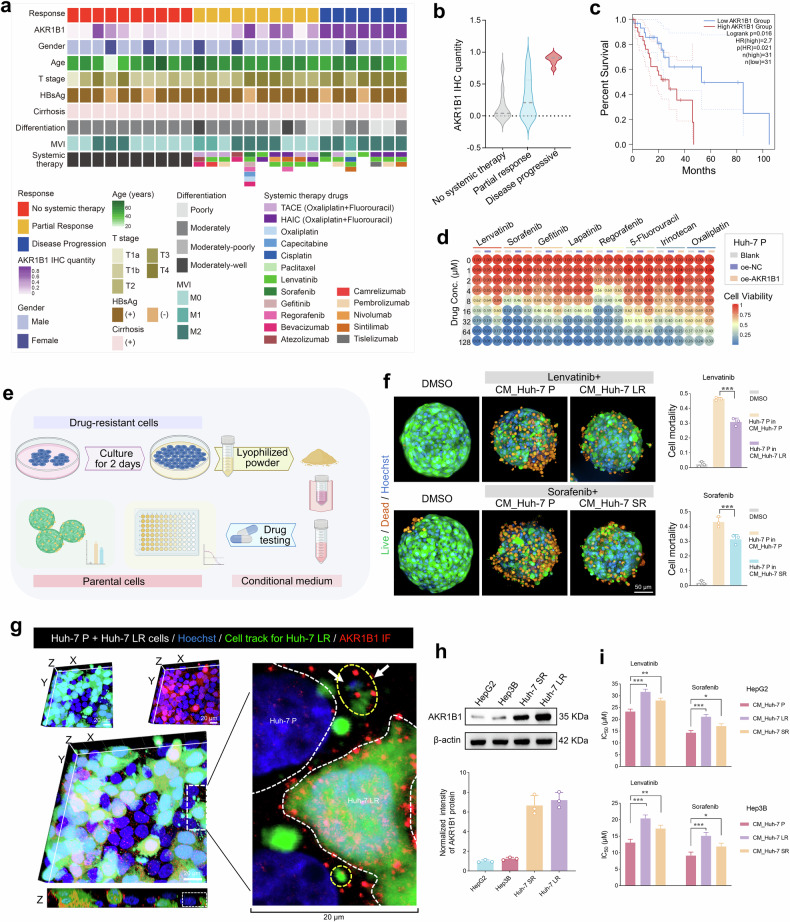
Associations of AKR1B1 expression with HCC patient prognosis and primary drug resistance. **a** The HCC cohort was divided into three clinical therapy subcohorts: no chemotherapy (NC), partial response (PR), and disease progression (DP). Clinical parameters are indicated in the heatmap. MVI: microvascular invasion; T stage: tumor stage; TACE: transcatheter arterial chemoembolization; HAIC: hepatic arterial infusion chemotherapy. **b** Comparison of AKR1B1 expression levels in tumor tissues among HCC patients in the NC, PR, and DP subcohorts. **c** OS rate of patients in the TCGA HCC iCluster_3 cohort in relation to AKR1B1 expression levels. Testing methods: Comparison of survival differences between the two groups: log-rank test; calculation of the hazard ratio (HR): Cox regression model. **d** Heatmap illustrating drug sensitivity in parental cells following AKR1B1 overexpression. **e** Schematic representation of CM extraction from drug-resistant cells and its application to parental cells. Schematic figures were generated with BioRender (https://app.biorender.com/). **f** Live/dead fluorescent probe analysis of drug resistance transfer and cell mortality in parental 3D microtissues cultured with conditioned medium from drug-resistant cells. Scale bar = 50 μm. Testing method: Unpaired Student’s t-test. **g** Cell tracing and IF analysis of AKR1B1 transfer from drug-resistant cells to parental cells. Cell tracing (green): drug-resistant cells; AKR1B1 (red); nuclei (blue). Scale bar = 20 μm. **h** WB analysis of AKR1B1 expression in various HCC cell lines and drug-resistant cells via statistical analysis. **i** Drug sensitivity evaluation was performed on the basis of IC_50_ values to assess drug resistance transfer between multiple HCC cell lines via CM. Testing method: Unpaired Student’s t-test. *p < 0.05, **p < 0.01, ***p < 0.001, ****p < 0.0001

These findings demonstrate that AKR1B1 expression is induced during the development of drug resistance. Moreover, patients with intrinsically elevated AKR1B1 expression tend to exhibit primary resistance to therapy. To explore this phenomenon, we collected pretreatment tumor tissues from HCC patients and stratified them into two groups on the basis of their clinical response: partial response (PR) and disease progression (DP) (Fig. 5a and Supplementary Table 1). IHC analysis of AKR1B1 expression revealed markedly higher AKR1B1 levels in tumors from DP patients than in those from PR patients (Fig. 5a, b and Supplementary Fig. 37). Consistently, proteomic data from Jiang et al. revealed markedly elevated AKR1B1 expression in the tumor tissues of HCC patients with poor prognosis compared with adjacent nontumorous tissues.^42^ AKR1B1 expression is progressively increased in higher-grade malignant subtypes^42^ (Supplementary Fig. 38). Additionally, data from David A. Wheeler’s study, which employed the iCluster algorithm to integrate information from five molecular platforms (DNA copy number variation, DNA methylation, mRNA expression, miRNA expression, and RPPA proteomics), classified HCC patients into three molecularly distinct subgroups. Among these clusters, iCluster_3 exhibited marked chromosomal instability, increased TP53 mutation rates, and widespread hypomethylation at multiple CpG loci.^43^ Survival analysis of TCGA data revealed that patients in this group with high AKR1B1 expression had significantly shorter survival times, highlighting the robust correlation between elevated AKR1B1 expression and unfavorable outcomes in HCC (Fig. 5c).

To further investigate the causal relationship between AKR1B1 and drug resistance, a stable AKR1B1-overexpressing cell line was established from parental Huh-7 cells, and its drug sensitivity was assessed (Supplementary Fig. 39). As shown in Fig. 5d and Supplementary Table 4, AKR1B1 overexpression substantially diminished the sensitivity of Huh-7 cells to various targeted therapies and chemotherapeutic agents, including regorafenib, gefitinib, lapatinib, irinotecan, 5-fluorouracil, and oxaliplatin, underscoring its critical function in mediating the drug response. Intriguingly, AKR1B1 overexpression also resulted in notable accumulation of intracellular lipid droplets, elevated glutathione levels, increased glutathione-regulating pathway activity, and the upregulation of key related proteins (Supplementary Fig. 40).

AKR1B1 was previously identified as a secreted protein detectable in the serum of patients with HCC (Fig. 4f). Consistent with these findings, increased AKR1B1 secretion was observed in the supernatants of drug-resistant cells. On the basis of these findings, we hypothesized that AKR1B1 may mediate the intercellular transmission of drug resistance, contributing to the increased resistance of previously sensitive cells. To validate this hypothesis, parental Huh-7 cells were cocultured with CM derived from drug-resistant cells (Fig. 5e). This approach significantly increased the resistance of parental Huh-7 cells to lenvatinib and sorafenib (Supplementary Fig. 41a and Supplementary Table 5). Furthermore, live/dead staining in 3D microtissue models confirmed that CM from drug-resistant cells protected against drug-induced cell death, as demonstrated by a decreased number of dead cells (Fig. 5f). We further examined the exosomal content in the conditioned medium, and WB analysis revealed high expression levels of the exosome marker proteins CD81 and CD63 (Supplementary Fig. 41b). Following the enrichment and extraction of exosomes from Huh-7 LR cells via nanoporous ultrafiltration, nanoparticle tracking analysis (NTA) revealed characteristic vesicle dimensions (D50: 89.0 ± 36.7 nm; D10‒D90: 63‒134 nm) and an initial particle concentration of (2.49 ± 0.08) × 10¹¹ particles over 24 h from 1 × 10⁷ cells (Supplementary Fig. 41c, d). This size distribution is consistent with previously reported HCC exosome profiles in Hep3B/MHCC97H models^44^ and Huh-7 SR systems.^45^ Moreover, we examined the involvement of exosomes in mediating drug resistance by depleting exosomes from conditioned medium. Exosome-depleted medium or medium containing an exosome secretion inhibitor (GW4869) was unable to confer enhanced drug resistance to parental cells (Supplementary Fig. 41e, f). The use of cell-tracking probes (green fluorescence) offered initial evidence supporting the role of exosomal vesicles in mediating drug resistance (Supplementary Fig. 42 and Supplementary Data 14). We observed significantly elevated levels of AKR1B1 in the supernatants of drug-resistant cells (Supplementary Fig. 43a). The colocalization of cell-tracking probes with AKR1B1 IF (red fluorescence) revealed that exosomal vesicles transport AKR1B1 from drug-resistant Huh-7 LR cells to parental Huh-7 cells, thereby promoting the acquisition of resistance (Fig. 5g). Furthermore, IF imaging confirmed the colocalization of AKR1B1 with the exosomal marker CD81 (Supplementary Fig. 43b), confirming that AKR1B1 can be transmitted via exosomes. Finally, the HepG2 and Hep3B cell lines, which exhibit low endogenous AKR1B1 expression, presented a significant decrease in drug sensitivity after exposure to CM from drug-resistant Huh-7 cells, mirroring the results observed in Huh-7 P cells (Fig. 5h, i and Supplementary Fig. 44). Collectively, these findings demonstrate that AKR1B1 mediates drug resistance transmission through extracellular exosomal vesicles, highlighting the strong association between AKR1B1 expression and poor prognosis in HCC patients.

### Clinical translational potential of the AKR1B1 inhibitor epalrestat in reversing HCC drug resistance

**Fig. 6:**
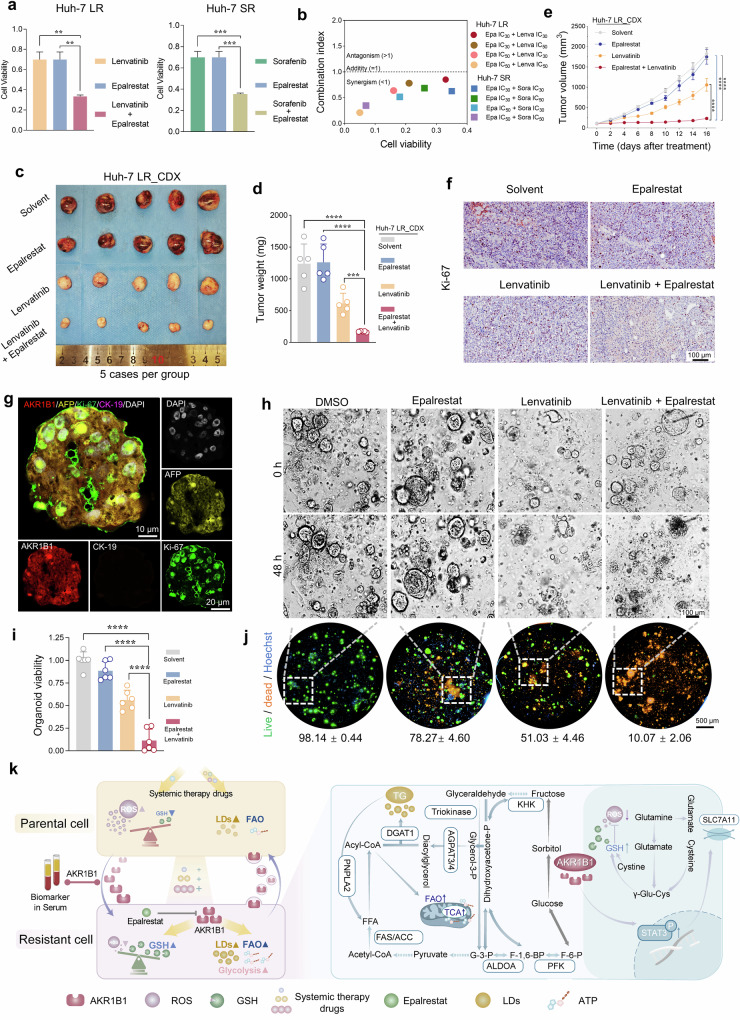
A combination therapy strategy mitigates drug resistance in HCC. **a** Evaluation of the activity of drug-resistant cells treated with epalrestat (IC_30_ = 27.50 μM) in combination with lenvatinib (IC_30_ = 43.32 μM) or sorafenib (IC_30_ = 8.89 μM). Testing method: Unpaired Student’s t-test. **b** CI analysis for the coadministration of epalrestat with lenvatinib or sorafenib. **c** Drug sensitivity evaluation in CDX models derived from drug-resistant cells following coadministration of epalrestat and lenvatinib (*n* = 5/group). CDX model drug application concentrations: solvent: 5‰ carboxymethyl cellulose sodium; epalrestat: 50 mg/kg/d; lenvatinib: 5 mg/kg/d; combined regimen: epalrestat 50 mg/kg/d + lenvatinib 5 mg/kg/d. **d** Tumor weight measurements in CDX models generated from drug-resistant cells following coadministration of epalrestat and lenvatinib. Testing method: Unpaired Student’s t-test. **e** Tumor growth kinetics in CDX models generated from drug-resistant cells following coadministration of epalrestat and lenvatinib. Testing method: Unpaired Student’s t-test. **f** Ki-67 IHC staining of tumor tissues from nude mice treated with the combination therapy. Scale bar = 100 μm. **g** IF analysis of AKR1B1-expressing HCC PDOs. DAPI (gray), AFP (yellow), AKR1B1 (red), CK19 (orange), and Ki-67 (green) are shown. Scale bar = 10 μm/20 μm. **h** Brightfield microscopy image of the impact of coadministration of epalrestat and lenvatinib on HCC PDOs at 0 h and 48 h. Scale bar = 100 μm. Organoid drug application concentrations: Epalrestat: 53.96 μM; lenvatinib: 50.61 μM; combined regimen: Epalrestat 53.96 μM + lenvatinib 50.61 μM. **i** Resazurin assay to evaluate the viability of HCC PDOs after 48 h of coadministration of epalrestat and lenvatinib. Testing method: Unpaired Student’s t-test. **j** Live/dead probe staining was used to assess the survival rate of HCC PDOs following 48 h of coadministration of epalrestat and lenvatinib. Scale bar = 500 μm. **k** Schematic representation of metabolic reprogramming in drug-resistant cells and the role of AKR1B1 in HCC drug resistance. *p < 0.05, **p < 0.01, ***p < 0.001, ****p < 0.0001

Encouragingly, a clinically approved drug, epalrestat, has emerged as a promising therapeutic agent that targets the key molecule AKR1B1. Epalrestat, which is currently approved for the prevention and treatment of diabetic peripheral neuropathy, has also demonstrated potential in oncology, as supported by its ongoing clinical trial for the treatment of metastatic triple-negative breast cancer (NCT03244358). To evaluate the effects of epalrestat on HCC cells, we initially assessed its cytotoxic effects on both parental and drug-resistant cell lines (Supplementary Fig. 45). The results demonstrated that epalrestat elicited markedly stronger growth-inhibitory effects in drug-resistant cells than in parental cells (IC_50_ of Huh-7 P: 85.01 ± 1.01 μM; IC_50_ of Huh-7 LR: 53.96 ± 0.98 μM; IC_50_ of Huh-7 SR: 67.22 ± 0.99 μM). Notably, combination drug testing revealed that epalrestat significantly enhanced the anticancer efficacy of lenvatinib or sorafenib in drug-resistant cell lines (Huh-7 LR and Huh-7 SR) and exhibited robust synergistic effects across various concentration combinations (combination index (CI) < 1) (Fig. 6a, b and Supplementary Fig. 46).

The therapeutic potential of epalrestat was further confirmed in vivo through a xenograft mouse model. After fully evaluating the safety of epalrestat in vivo, we conducted drug combination experiments (Supplementary Fig. 47). Compared with monotherapy with either epalrestat or lenvatinib, the combination of epalrestat and lenvatinib resulted in a significant reduction in tumor volume, slower tumor growth, and decreased tumor weight (Fig. 6c–e and Supplementary Fig. 48a, b). IHC staining for Ki-67 revealed that, compared with single-agent treatment, combination therapy significantly inhibited the proliferation of drug-resistant tumor cells (Fig. 6f and Supplementary Fig. 48c).

To further investigate the clinical relevance of epalrestat-lenvatinib combination therapy, we employed patient-derived organoid models of HCC. Four organoids derived from different HCC patients were selected, and AKR1B1 protein expression was subsequently quantified. Among these, organoid #3 presented comparatively elevated AKR1B1 expression (Supplementary Fig. 49a). Histopathological analyses, including H&E staining, IHC, and IF staining of the corresponding patient tumor tissues, confirmed that organoid #3 accurately recapitulated the expression patterns of key tumor-specific markers, such as AFP and CK18 (Fig. 6g and Supplementary Fig. 49b, c). Subsequent experiments demonstrated that the combination of lenvatinib and epalrestat significantly inhibited the growth of organoids and induced extensive cell death, thereby effectively countering drug resistance in HCC (Fig. 6h–j, Supplementary Fig. 50, and Supplementary Data 15).

In brief, this study demonstrated that in drug-sensitive HCC cells, conventional therapies primarily exert their antitumor effects by inducing oxidative stress-mediated cell death and reducing angiogenesis, ultimately leading to tumor shrinkage. However, prolonged exposure to standard therapies enables tumor cells to activate protective mechanisms, culminating in the development of complete drug resistance. Notably, while TKIs continue to suppress angiogenesis in resistant tumors, tumor growth remains unchecked. Our research revealed that AKR1B1 is a critical enzyme that plays a dual role in regulating glucose and lipid metabolism to increase energy supply and stress resistance, as well as modulating oxidative phosphorylation, thereby promoting cellular protection and the development of multidrug resistance. Importantly, we demonstrated that targeting AKR1B1 with the specific inhibitor epalrestat markedly enhances the therapeutic efficacy of existing systemic treatments (see mechanism illustration in Fig. 6k).

## Discussion

Resistance remains a critical obstacle to improving patient prognosis and prolonging survival in the systemic treatment of advanced HCC. The emergence of multidrug resistance (MDR) is recognized as a key contributor to the failure of systemic therapies. Currently, substantial research efforts are dedicated to unraveling the mechanisms underlying MDR, aiming to counteract this phenomenon and establish a theoretical basis for clinical strategies.^17,46,47^ In this study, drug-resistant HCC cell models were successfully established via the first-line targeted therapeutic agents sorafenib and lenvatinib, facilitating a comprehensive investigation of resistance mechanisms in systemic HCC treatment. After confirming the resistance of the constructed cell lines, cross-resistance and MDR phenomena were further observed in these cells. These findings indicate that resistance is more likely due to the adaptive reprogramming of tumor cells at the systemic level rather than the evasion of specific drug cytotoxic mechanisms. Furthermore, consistent with clinical observations, resistance frequently arises in combination therapy contexts, with resistant cells displaying analogous resistance profiles across various therapeutic regimens.

The mechanisms underlying tumor resistance are multifaceted and include tumor burden, growth kinetics, intratumoral heterogeneity, physical barriers, immune system interactions, the tumor microenvironment, untargeted cancer-driving factors, and the impact of therapeutic pressure.^48^ Insights from single-cell transcriptomic sequencing revealed that drug-resistant cells display heightened activity in stemness-related properties and metabolic regulation, conferring enhanced self-renewal capacity and adaptive plasticity. These alterations allow tumor cells to resist pharmacological interventions, thereby presenting increasingly complex therapeutic challenges. Notably, as EMT phenotypes and tumor stem cell-like characteristics are acquired, resistant cells exhibit markedly increased invasive potential, further amplifying resistance via a positive feedback loop.^49^

In recent years, metabolic reprogramming has attracted increasing attention for its critical role in mediating resistance to systemic cancer therapies. This phenomenon involves pathways related to nutrient uptake, biosynthesis, storage, transformation, and rapid ATP production, with glycolysis, fatty acid synthesis, and glutathione metabolism being particularly prominent. The ultimate goal of these reprogrammed metabolic pathways is to provide the essential building blocks necessary to satisfy the nutritional and energetic demands of cancer cells, thereby supporting their growth and resilience to environmental stressors.^50,51^ In this study, we utilized both in vitro and in vivo models to elucidate the critical roles of glucose‒lipid metabolic reprogramming and the glutathione metabolic pathway in enabling resistant cells to sustain growth and survive under pressure. Specifically, assessments of glycolytic energy production, triglyceride levels, lipid droplet accumulation, and the fatty acid-driven energy supply revealed that the metabolic activity of resistant cells was markedly elevated compared with that of their parental counterparts. This upregulation of energy and substrate metabolism enables resistant cells to adapt to stress-inducing conditions, functioning as a key driver of resistance. Further multiomics analyses revealed marked upregulation of molecules and metabolites associated with glucose‒lipid and glutathione metabolic pathways, highlighting the strong association between resistance and hyperactivation of these processes. While the glucose‒lipid metabolic pathway predominantly fulfills the energy demands essential for tumor cell survival, the glutathione metabolic pathway mitigates oxidative stress and prevents cell death. Collectively, these findings elucidate a comprehensive resistance mechanism wherein resistant cells leverage multiple metabolic reprogramming pathways to survive and evade therapeutic pressures. Notably, the drug-resistant models we developed are resistant not only to TKIs but also to common chemotherapeutic drugs. Given that the primary mechanism of action of many chemotherapeutic agents involves interference with DNA metabolism,^52,53^ we propose that drug-resistant cells counteract these effects by actively supplying abundant nucleic acid synthesis precursors through glycolysis, the PPP, one-carbon metabolism, and the TCA cycle, thereby driving therapeutic resistance.

Previous studies have shown that high fructose intake facilitates the initiation and progression of HCC in in vivo models with a complex tumor microenvironment.^34,54^ Furthermore, fructose metabolism plays a critical role in shaping cancer cell invasion and migration phenotypes.^55–57^ Fructose uptake and metabolism in vascular endothelial cells activate the Akt and Src signaling pathways, thereby significantly enhancing endothelial cell proliferation, migration, and tube formation.^56^ In this study, we identified a positive role of fructose metabolism in drug-resistant HCC cells. Through metabolic reprogramming, resistant cells activate the fructose metabolic pathway to promote lipogenesis and sustain survival. Our findings indicate that resistant cells depend not only on glycolysis-derived intermediates for lipid synthesis but also on alternative lipid biosynthesis pathways to sustain survival and growth under therapeutic pressure. Integrated transcriptomic and metabolomic analyses revealed AKR1B1 as a key enzyme that regulates both glucose‒lipid metabolism and glutathione metabolism. AKR1B1 exerts pleiotropic effects on physiological processes and has been implicated in the progression of various diseases, including cancer.^58^ In studies on breast and lung cancer, AKR1B1 was shown to promote tumor growth and metastasis by modulating the NF-κB‒Twist2 axis and the glucose‒polyol‒EMT pathway.^59,60^ However, the precise role of AKR1B1 in HCC progression and drug resistance development remains unclear.

We conducted a comprehensive investigation of AKR1B1 expression in drug-resistant HCC models from multiple perspectives. Our findings revealed significant upregulation of AKR1B1 expression in resistant cells compared with their parental counterparts. Both in vitro and in vivo experiments demonstrated that this elevated expression critically influences the development of drug resistance by regulating key metabolic processes. Furthermore, analysis of pathological samples from clinical HCC patients revealed high AKR1B1 expression in the tumor tissues of drug-resistant patients, whereas AKR1B1 expression was significantly lower in those who achieved partial remission. These findings suggest that AKR1B1 expression could serve as a predictive biomarker for the onset of drug resistance. Notably, as a secreted protein, AKR1B1 appears to mediate resistance transmission, suggesting that resistant cells may promote tumor heterogeneity by conferring resistance to previously drug-sensitive cells. Exosome secretion is primarily mediated through classical and nonclassical pathways. The classical pathway involves the formation of multivesicular bodies (MVBs), which are characterized by endosomal membrane invagination to form intraluminal vesicles (ILVs). These MVBs subsequently fuse with the plasma membrane, releasing ILVs into the extracellular space as exosomes. Nonclassical pathways include autophagy-mediated mechanisms, mitochondrial pathways, nuclear envelope pathways, secretory autophagy, and plasma membrane budding. These mechanisms facilitate exosome secretion through distinct processes that are generally independent of the conventional MVB‒plasma membrane fusion mechanism.^61–63^ AKR1B1, on the basis of tracking/staining assays of exosomes derived from drug-resistant cells, may be secreted via a combined mechanism involving both the classical pathway and nonclassical plasma membrane budding, potentially modulating the extracellular microenvironment.

This study is the first to establish a bidirectional relationship between AKR1B1 expression and resistance development in HCC. Specifically, AKR1B1 upregulation drives drug resistance acquisition in tumor cells, whereas resistance emergence further amplifies AKR1B1 expression. This reciprocal relationship highlights AKR1B1 as not only a critical biomarker of resistance but also a promising therapeutic target for overcoming resistance. Encouragingly, epalrestat, an AKR1B1 inhibitor already approved for diabetic neuropathy treatment, effectively mitigated drug resistance in both in vitro and in vivo HCC models. In combination with lenvatinib, this therapeutic strategy significantly enhanced tumor suppression in vivo compared with lenvatinib monotherapy. Importantly, epalrestat was well tolerated in our in vivo studies. The treated mice maintained a stable body weight, showed no significant hematological or biochemical abnormalities, and exhibited no treatment-related mortality during the 2-week administration period. This favorable safety profile supports its potential for combination therapy in HCC. Notably, prior studies have demonstrated the therapeutic potential of epalrestat in nonalcoholic steatohepatitis (NASH). In NASH mouse models, epalrestat mitigated hepatic inflammation and improved pathological conditions by suppressing NLRP3 inflammasome activation.^64^ In addition to its hepatic benefits, epalrestat has protective effects on other systems. In ischemic stroke mouse models, it preserves blood‒brain barrier (BBB) integrity by maintaining endothelial barrier function in brain microvascular endothelial cells (BMVECs), which is mediated through the modulation of the AR/AKT/mTOR signaling pathway, which suppresses apoptosis and excessive autophagy in BMVECs.^65^ In db/db mouse models of type 2 diabetes and its complications, epalrestat has been shown to have protective effects against diabetic nephropathy (DN) through the inhibition of aldose reductase and the regulation of the polyol pathway in renal inflammatory cells, suggesting a potential therapeutic strategy for DN.^66^ These findings, together with the multisystem protective effects of epalrestat and its demonstration of HCC-specific chemosensitization, suggest a favorable therapeutic window for its repurposing in combination oncology strategies. Future investigations should explore the potential context-dependent metabolic effects of AKR1B1 inhibition in nonmalignant hepatic compartments during anti-HCC therapy.

In summary, this study confirmed the occurrence of MDR in drug-resistant cell models during systemic therapy. Compared with their parental counterparts, resistant cells presented significantly elevated energy metabolism and glucose‒lipid metabolic activity. Integrated multiomics analyses further revealed increased activity in the glucose‒lipid and glutathione metabolic pathways, thereby constructing a comprehensive metabolic reprogramming map of drug-resistant HCC cells. Importantly, this study highlights the clinical importance of AKR1B1. Through rigorous analysis of clinical samples, we validated the feasibility of AKR1B1 as both a serum biomarker and a tissue biomarker. Furthermore, we comprehensively elucidated its potential as a therapeutic target and proposed a viable clinical drug application strategy. These findings offer valuable insights with significant potential for advancing the diagnosis and treatment of HCC, providing a solid foundation for future therapeutic interventions.

## Materials and Methods

The experimental details, including methods, antibody information, gene intervention sequences, and probe information, are provided in the Supplementary Information File.

### Cell culture and clinical samples

HCC cell lines, including Huh-7, HepG2, and Hep3B, were cultured in complete DMEM (12100046, Gibco). The growth medium was supplemented with 10% (vol/vol) fetal bovine serum (04-001-1ACS, Biological Industries) and penicillin‒streptomycin (100 U/ml) (15140-122, Gibco) in a 5% CO_2_-humidified incubator at 37 °C. The cells were passaged every 2–3 days.

To establish drug-resistant HCC cell models, we employed a long-term dose-escalation method by culturing parental Huh-7 cells in media containing lenvatinib (HY-10981, MedChemExpress) or sorafenib (HY-10201A, MedChemExpress). Specifically, lenvatinib or sorafenib was dissolved in complete culture medium via ultrasonic agitation and vortexing. The initial drug concentration was set at 1 μM, and the medium was changed every two days. The drug concentration was increased incrementally by 0.5–1.0 μM per step when the cells exhibited stable growth without signs of cytotoxicity. The maximum concentrations were 20 μM for lenvatinib and 5 μM for sorafenib, with resistant cells maintained and passaged at these concentrations. If cell growth became unstable, the drug concentration was reduced, and escalation was resumed once stability was restored. The development of resistant cell lines required 10 months for lenvatinib-resistant cells and 6 months for sorafenib-resistant cells.

Tissues, serum, and organoids derived from clinical HCC patients in this study were obtained from the Biospecimen Bank of Beijing Tsinghua Changgung Hospital, with all patients providing informed consent. This study adheres to the Declaration of Helsinki. This study received approval from the Ethics Committee of Beijing Tsinghua Changgung Hospital (Approval Numbers 23587-0-01 and 25332-0-01).

### Cell viability assay

Cell viability in 2D cultures was evaluated via the Cell Counting Kit-8 (CCK-8) assay (C0038, Beyotime Biotechnology). Specifically, cell suspensions (5000 cells/100 μl per well) were seeded into 96-well plates and cultured for 24 h. Targeted or chemotherapeutic agents were applied to each well at various concentrations and cultured for 48 h. Subsequently, 10 μl of CCK-8 reagent diluted at a 1:10 ratio (CCK-8 reagent:complete culture medium) was added to each well, including background wells. The plates were incubated for an additional 2–4 h, and the absorbance at 450 nm was measured via a multifunctional microplate reader (Synergy H1, BioTek). Each concentration gradient was tested in triplicate to ensure reproducibility. Cell viability was calculated via the following formula: cell viability = (absorbance of experimental wells − background wells)/(absorbance of control wells − background wells).

### Immunofluorescence (IF) staining and immunohistochemical (IHC) staining

For IF staining, cells and spheroids were fixed in 4% PFA, permeabilized with 0.2% Triton X-100, blocked with 10% goat or donkey serum, and incubated with primary antibodies at 4 °C overnight. The samples were then incubated with secondary antibodies for 1 h in the dark at room temperature, followed by incubation with 4′,6-diamidino-2-phenylindole (DAPI) for nuclear staining. Images of cultured cells, spheroids, and organoids were captured via an Operetta high-content imaging system (PerkinElmer) equipped with a 20× Plan Fluor objective.

Multicolor immunofluorescence labeling experiments were performed on paraffin-embedded and frozen sections via the NEON-DendronFluor® Multicolor Fluorescent Labeling System Kit (Histova Biotechnology) following the manufacturer’s protocol. After multiple rounds of antigen retrieval, blocking, primary antibody incubation, and incubation with a fluorescent secondary antibody, the slides were mounted and scanned via the PhenoImager HT system (AKOYA).

For IHC, tumor tissues were fixed in 4% PFA, embedded in paraffin, and sectioned into 5 μm slices. Subsequent steps were performed via a vector kit (VECTASTAIN^®^ Elite^®^ ABC-HRP Kit_PK-6200, Avidin/Biotin Blocking Kit_SP-2001, Vector NovaRED^®^ Substrate Kit_SK-4800, Vector Laboratories) according to the manufacturer’s instructions. IHC images were captured via a digital slide scanner (3D Histech).

### Metabolomics and metabolic flux analysis

Metabolomic and metabolic flux analyses were conducted by LipidALL Technologies Co., Ltd. A brief description of the experimental methods is provided below.

Untargeted Metabolomics: Polar metabolites were extracted from cells and analyzed via LC‒MS via the same system as described above. Metabolites were separated via both reversed-phase liquid chromatography (RPLC) and hydrophilic interaction liquid chromatography (HILIC) columns, with specific scan ranges set for each. MS/MS analyses were performed in information-dependent acquisition mode, and the data were processed to annotate ion identities via MarkerView 1.3 and PeakView 2.2 software.

FFA analysis: FFAs were extracted via a modified Bligh and Dyer method and analyzed via high-performance liquid chromatography (HPLC) coupled with a triple quadrupole/ion trap mass spectrometer. Normal-phase HPLC was employed for lipid separation, and FFAs were quantified via internal standards for accurate measurement.

High-resolution metabolic flux analysis: Polar metabolites were extracted and analyzed for metabolic flux, with alpha-keto acids derivatized for enhanced detection. The analysis was conducted on an Agilent 1290 II UPLC coupled to a Sciex 5600+ quadrupole-TOF MS, with specific columns and MS parameters applied. Internal standard normalization was applied to correct for endogenous metabolites in the samples.

### RNA sequencing assay

Total RNA was extracted via TRIzol reagent. Two independent samples from each group (Huh-7 P, Huh-7 LR, and Huh-7 SR) were used for RNA sequencing, which was conducted by Biomarker Technologies (Beijing, China). Protein‒protein interaction (PPI) networks for differentially expressed genes were predicted via the Search Tool for the Retrieval of Interacting Genes (STRING; http://string-db.org). Cytoscape bioinformatics software was used to visualize the molecular interaction networks. The Molecular Complex Detection (MCODE) algorithm was applied to identify molecular complexes and densely connected regions within the PPI. Kyoto Encyclopedia of Genes and Genomes (KEGG) pathway analysis was conducted via OmicShare tools, an online bioinformatics platform (https://www.omicshare.com).

### AKR1B1-enzyme-linked immunosorbent assay kit (ELISA) detection

The levels of AKR1B1 protein in the serum of HCC patients and cell culture supernatants were measured via an ELISA kit (EH2164, FineTest). Briefly, reagents and samples were equilibrated to room temperature, and washing buffer was prepared. Biotin-labeled antibodies and streptavidin-avidin-biotin complex (SABC) working solutions were prepared. Standards and samples were added to the microplate, incubated at 37 °C, and washed. Biotin-labeled antibodies and SABC were subsequently added, followed by an additional washing step. Subsequently, 3,3′,5,5′-tetramethylbenzidine (TMB) substrate was added, and the mixture was incubated in the dark. A stop solution was added, and the absorbance at 450 nm was measured with a microplate reader. The concentration of the target protein in the samples was calculated on the basis of the standard curve.

## Supplementary information

Supporting information

Supplementary Data 1

Supplementary Data 2

Supplementary Data 3

Supplementary Data 4

Supplementary Data 5

Supplementary Data 6

Supplementary Data 7

Supplementary Data 8

Supplementary Data 9

Supplementary Data 10

Supplementary Data 11

Supplementary Data 12

Supplementary Data 13

Supplementary Data 14

Supplementary Data 15

## Data Availability

All data needed to evaluate the conclusions in the article are present in the article and the Supplementary Materials. The raw data for bulk RNA-seq have been deposited in the GSA-Human database under accession number HRA010999 (https://ngdc.cncb.ac.cn/gsa-human/browse/HRA010999). The raw data for single-cell RNA-seq have been deposited in the GSA-Human database under accession number HRA011003 (https://ngdc.cncb.ac.cn/gsa-human/browse/HRA011003). The raw data for metabolomics have been deposited in the OMIX database under accession number OMIX010653 (https://ngdc.cncb.ac.cn/omix/release/OMIX010653). The raw data for metabolic flux have been deposited in the OMIX database under accession number OMIX010678 (https://ngdc.cncb.ac.cn/omix/release/OMIX010678). All raw data mentioned above are openly accessible.
