# Supplementary material for: Targeting AKR1B1 inhibits metabolic reprogramming to reverse systemic therapy resistance in hepatocellular carcinoma
Source: Signal Transduct Target Ther. 2025 Aug 1;10:244. doi: 10.1038/s41392-025-02321-9 (PMC12317016; doi:10.1038/s41392-025-02321-9)
Supplement: Supplementary file 1 — Supporting information [file 41392_2025_2321_MOESM1_ESM.docx]

**Supplementary** **Materials for**

Targeting AKR1B1 inhibits metabolic reprogramming to reverse systemic therapy resistance in hepatocellular carcinoma

Qi Wang, Juan Liu, Ming Yang, Jun Zhou, Yaxuan Li, Jingjing Zheng, Hao Jia, Shuhua Yue, Yinpeng Le, Yuxin Su, Wenrui Ma, Ni An, Yunfang Wang, Jiahong Dong

**Content**

[MATERIALS AND METHODS 5](#_Toc201188951)

[*Cell culture, genetic intervention and clinical samples* 5](#_Toc201188952)

[*Cell viability assay* 6](#_Toc201188953)

[*Cell line-derived xenograft* 6](#_Toc201188954)

[*Immunofluorescence (IF) staining and Immunohistochemical (IHC) staining* 7](#_Toc201188955)

[*Metabolomics and metabolic flux analysis* 8](#_Toc201188956)

[*Sorbitol content detection* 10](#_Toc201188957)

[*Cysteine content detection* 10](#_Toc201188958)

[*RNA sequencing assay* 10](#_Toc201188959)

[*Cellular energy metabolism analysis* 11](#_Toc201188960)

[*Nile red, Glutathione (GSH) and Reactive Oxygen Species (ROS) staining* 11](#_Toc201188961)

[*Measurement of NAD(H) and NADP(H) concentrations* 11](#_Toc201188962)

[*Triglyceride detection* 12](#_Toc201188963)

[*Raman spectroscopy and stimulated Raman scattering (SRS) imaging* 12](#_Toc201188964)

[*Fluorescent probe tracing for fatty acid uptake* 12](#_Toc201188965)

[*Detection of fatty acid β-oxidation* 12](#_Toc201188966)

[*Single-cell RNA sequencing* 13](#_Toc201188967)

[*Western Blotting* 13](#_Toc201188968)

[*AKR1B1-Enzyme-Linked immunosorbent assay Kit (ELISA) detection* 14](#_Toc201188969)

[*Preparation of conditioned medium (CM)* 14](#_Toc201188970)

[*Exosome isolation and characterization of concentration and size distribution* 14](#_Toc201188971)

[*Cell probe tracking experiment* 15](#_Toc201188972)

[*Statistical methods* 15](#_Toc201188973)

[Supplementary Fig. 1. Construction and evaluation of drug-resistant cell models. 16](#_Toc201188974)

[Supplementary Fig. 2. Evaluation of multidrug sensitivity in drug-resistant cells. 17](#_Toc201188975)

[Supplementary Fig. 3. Evaluation of multidrug sensitivity in 3D drug-resistant microtissue models. 18](#_Toc201188976)

[Supplementary Fig. 4. Evaluation of combined-drug sensitivity in drug-resistant cell and 3D microtissue models. 19](#_Toc201188977)

[Supplementary Fig. 5. Evaluation of drug sensitivity in tumorigenic tissue of drug-resistant cells, along with assessment of cell proliferation and triglyceride content. 20](#_Toc201188978)

[Supplementary Fig. 6. RNA-seq analysis of drug-resistant cells. 21](#_Toc201188979)

[Supplementary Fig. 7. t-SNE plot of single-cell sequencing data distinguishing between drug-resistant cells and parental cells. 21](#_Toc201188980)

[Supplementary Fig. 8. Pseudotime analysis of drug-resistant cells and parental cells. 22](#_Toc201188981)

[Supplementary Fig. 9. CytoTRACE analysis of stemness in drug-resistant cells and parental cells. 23](#_Toc201188982)

[Supplementary Fig. 10. Transcription factor expression analysis of drug-resistant cells with the "on" state. 23](#_Toc201188983)

[Supplementary Fig. 11. Transcription factor expression analysis of parental cells with the "on" state. 24](#_Toc201188984)

[Supplementary Fig. 12. Cluster-based GSEA from single-cell sequencing data. 25](#_Toc201188985)

[Supplementary Fig. 13. Seahorse energy metabolism analysis of cellular glycolytic capacity and glycolytic reserve. Testing method: Unpaired Student’s t-test. 25](#_Toc201188986)

[Supplementary Fig. 14. Detection of key enzyme levels in cellular redox reactions. 26](#_Toc201188987)

[Supplementary Fig. 15. Lipid content assessment in drug-resistant cells. 27](#_Toc201188988)

[Supplementary Fig. 16. Raman spectroscopy detection and statistical analysis of lipid levels in drug-resistant cells. Scale bar = 20 μm. Testing method: Unpaired Student’s t-test. 28](#_Toc201188989)

[Supplementary Fig. 17. Stimulated Raman scattering (SRS) detection of cellular FFA uptake. 29](#_Toc201188990)

[Supplementary Fig. 18. Detection of FFAs uptake efficiency in drug-resistant cells using the FFA analog tracing probe. FFAs analog (green), nucleus (blue). Scale bar = 100 μm. 30](#_Toc201188991)

[Supplementary Fig. 19. Detection of FFA β-oxidation (FAO) levels in drug-resistant cells. Testing method: Unpaired Student’s t-test. 30](#_Toc201188992)

[Supplementary Fig. 20. Assessment of drug sensitivity and lipid droplet levels in stage-specific drug-resistant cells. 31](#_Toc201188993)

[Supplementary Fig. 21. Correlation analysis between intracellular lipid droplet levels and drug sensitivity in drug-resistant cells. 32](#_Toc201188994)

[Supplementary Fig. 22. Intracellular glycolysis metabolic flux detection. “M+number” indicates the number of additional ¹³C atoms in the metabolite molecule. “*” indicates a significant difference between the corresponding substance in Huh-7 LR/SR and Huh-7 P, with p < 0.05. 33](#_Toc201188995)

[Supplementary Fig. 23. Intracellular pentose phosphate pathway metabolic flux detection. “M+number” indicates the number of additional ¹³C atoms in the metabolite molecule. “*” indicates a significant difference between the corresponding substance in Huh-7 LR/SR and Huh-7 P, with p < 0.05. 34](#_Toc201188996)

[Supplementary Fig. 24. Intracellular TCA cycle metabolic flux detection. “M+number” indicates the number of additional ¹³C atoms in the metabolite molecule. “*” indicates a significant difference between the corresponding substance in Huh-7 LR/SR and Huh-7 P, with p < 0.05. 35](#_Toc201188997)

[Supplementary Fig. 25. The impact of key enzymes in polyol, fructose metabolic pathways and lipogenesis pathways on the drug sensitivity of resistant cells. 36](#_Toc201188998)

[Supplementary Fig. 26. Identification of AKR1B1 in drug-resistant cells. 37](#_Toc201188999)

[Supplementary Fig. 27. IF staining and quantification of AKR1B1 in HCC patients tumor tissues. 38](#_Toc201189000)

[Supplementary Fig. 28. Assessment of drug sensitivity in drug-resistant cells after intervention of AKR1B1. 39](#_Toc201189001)

[Supplementary Fig. 29. Analysis of drug sensitivity in Huh-7 SR after knockdown of AKR1B1. 40](#_Toc201189002)

[Supplementary Fig. 30. In vivo experiments following the intervention of AKR1B1 in drug-resistant cells. 41](#_Toc201189003)

[Supplementary Fig. 31. Intervention of AKR1B1 in drug-resistant cells affects metabolic reprogramming. 41](#_Toc201189004)

[Supplementary Fig. 32. The impact of AKR1B1 on the glutathione regulating pathway. 42](#_Toc201189005)

[Supplementary Fig. 33. Glutathione regulating pathway enzyme profiles in Lenvatinib-treated tumors post AKR1B1 knockdown. Testing method: Unpaired Student’s t-test. 42](#_Toc201189006)

[Supplementary Fig. 34. Predicting the transcription factors of AKR1B1 using public databases. 42](#_Toc201189007)

[Supplementary Fig. 35. Drug-resistant cells upregulate FOSL2 through the Wnt signaling pathway. 43](#_Toc201189008)

[Supplementary Fig. 36. Prediction of FOSL2 binding sites with AKR1B1 and functional analysis of downstream target genes. 44](#_Toc201189009)

[Supplementary Fig. 37. AKR1B1 IHC staining of tumor tissues from HCC patients with no drug use (N/A), partial response (PR), and disease progression (DP). Scale bar = 2 mm and 40 μm. 45](#_Toc201189010)

[Supplementary Fig. 38. Protein expression levels of AKR1B1 in tumor tissues versus adjacent normal tissues in HCC patients categorized by proteomic subclasses^10^. Testing method: Unpaired Student’s t-test. 46](#_Toc201189011)

[Supplementary Fig. 39. WB detection of AKR1B1 expression in parental cells after overexpression of AKR1B1. Testing method: Unpaired Student’s t-test. 46](#_Toc201189012)

[Supplementary Fig. 40. Metabolic detection of parental cells overexpressing AKR1B1. 47](#_Toc201189013)

[Supplementary Fig. 41. Mediation of drug resistance transfer by conditional medium. 48](#_Toc201189014)

[Supplementary Fig. 42. Cell tracing probe detection of material transfer from drug-resistant cells to parental cells. Cell tracing (green) — drug-resistant cells, cell membrane (red), nucleus (blue). Scale bar = 10 μm. 49](#_Toc201189015)

[Supplementary Fig. 43. Exosome and AKR1B1 colocalization detection. 50](#_Toc201189016)

[Supplementary Fig. 44. Conditioned medium from drug-resistant cells enhances drug resistance in multiple types of HCC cells. 51](#_Toc201189017)

[Supplementary Fig. 45. Cellular activity curve and IC_50_ values of drug-resistant cells and parental cells treated with Epalrestat. Testing method: Unpaired Student’s t-test. 51](#_Toc201189018)

[Supplementary Fig. 46. Inhibitory concentration detection of Epalrestat and targeted therapies as single agents in drug-resistant cell lines. 52](#_Toc201189019)

[Supplementary Fig. 47. Epalrestat toxicity testing in vivo. 53](#_Toc201189020)

[Supplementary Fig. 48. In vivo experiments to test the improvement of drug resistance by combination therapy. 54](#_Toc201189021)

[Supplementary Fig. 49. Identification of HCC PDOs. 55](#_Toc201189022)

[Supplementary Fig. 50. Live/dead probe staining for the statistical analysis of the survival rate of HCC PDOs after 48 h of drug treatment. Testing method: Unpaired Student’s t-test. 55](#_Toc201189023)

[Supplementary Table 1. Systemic therapy-related clinical and pathological information of HCC patients. 56](#_Toc201189024)

[Supplementary Table 2. Clinical and pathological information of HCC patients with or without application of systemic therapy. 59](#_Toc201189025)

[Supplementary Table 3. IC_50_ values of drug-resistant cells with AKR1B1 knockdown after treatment with drugs. 62](#_Toc201189026)

[Supplementary Table 4. IC_50_ values of drug-resistant cells with AKR1B1 overexpression after treatment with drugs. 63](#_Toc201189027)

[Supplementary Table 5. IC_50_ values for drug resistance transfer mediated by conditional medium.  63](#_Toc201189028)

[Supplementary Table 6. The List of gene intervention used in this study. 64](#_Toc201189029)

[Supplementary Table 7. The List of primary antibodies used in this study. 65](#_Toc201189030)

[Supplementary Table 8. The List of fluorescence probes used in this study. 66](#_Toc201189031)

[Supplementary Table 9. The List of assay kits used in this study. 67](#_Toc201189032)

[Supplementary Data List. 68](#_Toc201189033)

## MATERIALS AND METHODS

### *Cell culture, genetic intervention and clinical samples*

HCC cell lines, including Huh-7, HepG2, and Hep3B, were cultured in DMEM (12100046, Gibco) complete medium. The growth medium was supplemented with 10% (vol/vol) fetal bovine serum (04-001-1ACS, Biological Industries) and penicillin-streptomycin (100 U/ml) (15140-122, Gibco) in a 5% CO_2_-humidified incubator at 37 °C. Cells were passaged every 2–3 days.

To establish drug-resistant HCC cell models, we employed a long-term dose-escalation method by culturing parental Huh-7 cells in media containing Lenvatinib (HY-10981, MedChemExpress) or Sorafenib (HY-10201A, MedChemExpress). Specifically, Lenvatinib or Sorafenib was dissolved in complete culture medium using ultrasonic agitation and vortexing. The initial drug concentration was set at 1 μM, and the medium was refreshed every two days. The drug concentration was increased incrementally by 0.5–1.0 μM per step when cells exhibited stable growth without signs of cytotoxicity. Maximum concentrations were 20 μM for Lenvatinib and 5 μM for Sorafenib, with resistant cells maintained and passaged at these levels. If cell growth became unstable, the drug concentration was reduced, and escalation resumed once stability was restored. The development of resistant cell lines required 10 months for Lenvatinib-resistant cells and 6 months for sorafenib-resistant cells. While both resistant lines were characterized, the superior cross-resistance profile and metabolic plasticity of the LR model motivated its selection for single-cell sequencing and subsequent mechanistic validation, ensuring maximal relevance to clinical multidrug resistance patterns.

For 3D cell spheroid culture, we developed a novel method to generate and culture cell spheroids with high throughput. Briefly, a 0.8% agarose solution was prepared using agarose powder (50002, SeaKem) and double-distilled water, then heated in a microwave oven until fully dissolved. Next, the solution was rapidly poured into each well of the culture plate in a biosafety cabinet. After 30 min of ultraviolet light irradiation of the plates, a cell suspension containing 600 cells per well was added for spheroid culture. The composition of the culture medium and culture conditions were identical to those used for 2D cell cultures.

Information on lentivirus (Shanghai Genechem Co.,Ltd.) and small interfering RNA (Shanghai GenePharma Co.,Ltd.) sequences is provided in **Supplementary Table 6**. The lentivirus infection workflow is as follows (based on the operational manual from the virus construction provider):

(1) Pre-experimental phase: On Day 1, prepare a cell suspension with a density of 3~5×10⁴/ml, seed it into a 96-well plate, and culture at 37°C for 16~24 hours until the cell confluence reaches 20~30%. On Day 2, calculate the required virus volume using the formula “virus volume (μl) = (MOI × cell number) / virus titer (TU/ml)”, add the virus and infection-enhancing solution, gently mix, and continue culturing. Finally, detect infection efficiency via fluorescence microscopy or flow cytometry to determine the optimal MOI and infection-enhancing solution type.

(2) Formal experimental workflow: On Day 1, seed cells at the same density into culture plates and culture until 20~30% confluence. On Day 2, prepare the virus mixture based on the pre-determined MOI, remove the old medium, add the mixture, gently mix, and incubate at 37°C for 12-24 hours. On Day 3, replace the medium with fresh complete medium and continue culturing for 48-72 hours to observe infection effects.

(3) Stable cell line screening: At 48 hours post-infection, add 5 μg/ml Puromycin and include uninfected wild-type cells as a control. Screen for 3-5 days until the control group is completely eliminated.

Tissues, serum, and organoids derived from clinical HCC patients in this study were obtained from the Biospecimen Bank of Beijing Tsinghua Changgung Hospital, with all patients providing informed consent. This study adheres to the Declaration of Helsinki. This study received approval from the Ethics Committee of Beijing Tsinghua Changgung Hospital (Approval Number: 23587-0-01 and 25332-0-01).

### *Cell viability assay*

Cell viability in 2D cultures was evaluated using the Cell-Counting-Kit-8 (CCK-8) assay (C0038, Beyotime Biotechnology). Specifically, cell suspensions (5000 cells/100 μl per well) were seeded into 96-well plates and cultured for 24 h. Targeted or chemotherapeutic agents, including Gefitinib (HY-50895, MCE), Regorafenib (HY-10331, MCE), Lapatinib (HY-50898, MCE), Oxaliplatin (HY-17371, MCE), Irinotecan (HY-16562, MCE), and 5-fluorouracil (S1209, Selleck), were applied to each well at various concentrations and cultured for 48 h. Subsequently, 10 μl of CCK-8 reagent, diluted at a 1:10 ratio (CCK-8 reagent: complete culture medium), was added to each well, including background wells. The plates were incubated for an additional 2-4 h, and absorbance at 450 nm was measured using a multifunctional microplate reader (Synergy H1, BioTek). Each concentration gradient was tested in triplicate to ensure reproducibility. Cell viability was calculated using the formula: Cell viability = (absorbance of experimental wells − background wells) / (absorbance of control wells − background wells).

Cell spheroids and organoids were additionally stained using the LIVE/DEAD™ Viability/Cytotoxicity Kit (L3224, ThermoFisher Scientific) or the Resazurin assay kit (R7017, Sigma). The spheroids were washed and stained with Calcein AM and ethidium homodimer-1 (EthD-1), and confocal images were acquired using an Operetta High-Content Imaging System (PerkinElmer) equipped with a 20× Plan Fluor objective. A stack of 20 planes, separated by 5 μm, was acquired, beginning at the well bottom and covering the lower half of each spheroid. All individual images were saved and used for automated quantitative analysis with Harmony^®^ 4.1 High-Content Imaging and Analysis Software. The Resazurin assay evaluates organoid activity by detecting the culture supernatant, with fluorescent intensity measured at excitation/emission wavelengths of 530 nm/590 nm.

### *Cell line-derived xenograft*

Female BALB/c immunodeficient nude mice (4–5 weeks old, ~20 g) were purchased from Viton Lever Biotechnology Co., Ltd. (Beijing). The study was approved by the Animal Welfare and Ethics Committee (Approval Number: PA23062801). Animal care and experiments were conducted in strict accordance with the “Guide for the Care and Use of Laboratory Animals” and the “Principles for the Utilization and Care of Vertebrate Animals”. At the end of the experiment, euthanasia was performed using CO₂ inhalation (displacement rate of 30% chamber volume per minute), followed by cervical dislocation to confirm death. In line with the 3Rs principles (Replacement, Reduction, Refinement), statistical power analysis of the expected effect size, and experimental designs from prior subcutaneous hepatocellular carcinoma xenograft studies ^1-3^, five animals were included per group in this study.

Experiment 1: Drug Sensitivity Assay in Tumorigenic Models of Parental and Drug-Resistant Cell Lines. Cell suspensions were mixed 1:1 with cold Matrigel, and 7×10⁶ cells in 150 μl were subcutaneously injected into the dorsal region of each mouse (n = 5 per group). Once the subcutaneous tumor reached a diameter of 6 mm, the animals were randomly divided into four groups. The Huh-7 P-solvent group and Huh-7 LR-solvent group were administered 0.5% carboxymethyl cellulose sodium solution, while the Huh-7 P-drug group and Huh-7 LR-drug group were treated with lenvatinib (5 mg/kg/d) via oral gavage. Tumor volume and body weight were measured every other day, and tumor volume (mm³) was calculated as: 1/2 × longest tumor diameter × (shortest tumor diameter)². After two weeks of treatment, tumors were harvested, photographed, cryopreserved, and fixed for embedding.

Experiment 2: Drug Sensitivity Assay in AKR1B1 Knockdown Drug-Resistant Cell Models. The experimental conditions were the same as in Experiment 1. Animals were divided into six groups: blank-solvent group, sh-NC-solvent group, and sh-AKR1B1-solvent group were given 0.5% carboxymethyl cellulose sodium solution; blank-drug group, sh-NC-drug group, and sh-AKR1B1-drug group were treated with Lenvatinib (5 mg/kg/d) via oral gavage.

Experiment 3: Sensitivity Assay of Combination Therapy in Drug-Resistant Cell Models. The experimental conditions were the same as in Experiment 1. Animals were divided into four groups: solvent group (0.5% carboxymethyl cellulose sodium solution), Epalrestat group (50 mg/kg/d) (HY-66009, MedChemExpress), Lenvatinib group (5 mg/kg/d), and combination therapy group (Epalrestat 50 mg/kg/d + Lenvatinib 5 mg/kg/d), with all treatments administered via oral gavage.

### *Immunofluorescence (IF) staining and Immunohistochemical (IHC) staining*

For IF staining, cells and spheroids were fixed in 4% PFA, permeabilized with 0.2% Triton X-100, blocked with 10% goat or donkey serum, and incubated with primary antibodies at 4 °C overnight. They were then incubated with secondary antibodies for 1 h in the dark at room temperature, followed by 4′,6-diamidino-2-phenylindole (DAPI) incubation for nuclear staining. Images of cultured cells, spheroids, and organoids were captured using an Operetta High Content Imaging System (PerkinElmer) equipped with a 20× Plan Fluor objective.

Multicolor immunofluorescence labeling experiments were performed on paraffin-embedded and frozen sections using the NEON-DendronFluor^®^ Multicolor Fluorescent Labeling System Kit (Histova Biotechnology), following the manufacturer’s protocol. After multiple rounds of antigen retrieval, blocking, primary antibody incubation, and fluorescent secondary antibody incubation, the slides were mounted and scanned using the PhenoImager HT system (AKOYA).

For IHC, tumor tissues were fixed in 4% PFA, embedded in paraffin, and sectioned into 5 μm slices. Subsequent steps were performed using the Vector kit (VECTASTAIN^®^ Elite^®^ ABC-HRP Kit_PK-6200, Avidin/Biotin Blocking Kit_SP-2001, Vector NovaRED^®^ Substrate Kit_SK-4800, Vector Laboratories) according to the manufacturer’s instructions. IHC images were captured using a digital slide scanner (3D Histech). Information on antibodies is provided in **Supplementary Table 7**.

### *Metabolomics and metabolic flux analysis*

Metabolomics and metabolic flux analyses were conducted by LipidALL Technologies Co., Ltd. A brief description of the experimental methods is provided below.

Untargeted Metabolomics: Untargeted metabolomics was conducted at LipidALL Technologies. Polar metabolites were extracted from cells using 1mL of ice-cold methanol containing 0.224mM phenylhydrazine. Samples were incubated at 1500 rpm for 30 min at 4 ^o^C. At the end of the incubation, samples were kept at -20 ^o^C for 1 h for derivatisation of alpha-keto acids ^4^.Then, samples were centrifuged for 15 min at 12 000 rpm at 4 ^o^C. Clean supernatant was transferred to a new tube and dried in a SpeedVac under H_2_O mode. Total protein content was determined from the dried pellet using the Pierce^®^ BCA Protein Assay Kit according to the manufacturer’s protocol. The dried extract was reconstituted in 5% acetonitrile in water prior to LC-MS analysis on an Agilent 1290 II UPLC coupled to Sciex 5600+ quadrupole-TOF MS. For reverse phase liquid chromatography (RPLC), polar metabolites were separated on a Waters ACQUITY HSS-T3 column (3.0 × 100 mm, 1.8 μm), while a Waters ACQUITY BEH Amide column (2.1 × 100 mm, 1.7 μm) was utilized for hydrophilic interaction liquid chromatography (HILIC). MS parameters for detection were: ESI source voltage negative ion mode −4.5 kV; vaporizer temperature, 500 °C; drying gas (N_2_) pressure, 50 psi; nebulizer gas (N_2_) pressure, 50 psi; curtain gas (N_2_) pressure, 35 psi; The scan ranges were set at *m/z* 60-700 during RPLC, and *m/z* 70-850 during HILIC analysis, respectively ^5^. Information-dependent acquisition mode was used for MS/MS analyses of the metabolites. Collision energy was set at (-) 35 ± 15 eV. Data acquisition and processing were performed using Analyst^®^ TF 1.7.1 Software (AB Sciex, Concord, ON, Canada). All detected ions were extracted using MarkerView 1.3 (AB Sciex, Concord, ON, Canada) into Excel in the format of 2D matrix, including mass to charge ratio (m/z), retention time, and peak areas, and isotopic peaks were filtered. PeakView 2.2 (AB Sciex, Concord, ON, Canada) was applied to extract MS/MS data and perform comparisons with the Metabolites database (AB Sciex, Concord, ON, Canada), HMDB, and standard references to annotate ion identities ^6^. A cocktail of isotopically-labeled internal standards (IS) purchased from Cambridge Isotope Laboratories were spiked into the samples for metabolite quantitation, including L-Phenylalanine-d_8_, L-Tryptophan-d_8_, L-Isoleucine-d_10_, L-leucine-d_10_, L-Methionine-d_3_, L-Valine-d_8_, L-Proline-d_7_, L-Alanine-d_4_, DL-Serine-d_3_, L-Glutamine-d_5_, L-Aspartic acid-d_3_, L-Arginine-d_7_, L-Glutamate-d_5_, L-Lysine-d_9_, L-Histidine-D_5_, Taurine-^13^C_2_, Betaine-d_11_, Urea-(^13^C,^15^N_2_), L-lactate-d_3_, Trimethylamine N-oxide-d_9_, Choline-d_13_, Malic acid-d_3_, Citric acid-d_4_, Succinic acid-d_4_, Fumaric acid-d_2_, Hypoxanthine-d_3_, Xanthine-^15^N_2_, Thymidine (^13^C_10_,^15^N_2_), Inosine-^15^N_4_, Cytidine-^13^C_5_, Uridine-d_2_, Methylsuccinic acid-d_6_, Benzoic acid-d_5_, Creatine-d_3_, Creatinine-d_3_, Glutaric acid-d_4_, Hippuric acid-d_5_, Kynurenic acid-d_5_, L-Citrulline-d_4_, L-Threonine-(^13^C_4_, ^15^N), L-Tyrosine-d_7_, P-cresol sulfate-d_7_, Sarcosine-d_3_, Trans-4-hydroxy-L-proline-d_3_, Uric acid-(^13^C; ^15^N_3_), Carnitine-C16:0-d_3_, Carnitine-C12:0-d_9_, Carnitine-C14:0-d_9_, Glycodeoxycholate-d_4_, L-Carnitine trimethyl-d_9_, L-Asparagine-13C4. Peak areas of endogenous metabolites were normalized to the areas of their corresponding isotopically labeled structural analogues for quantitation. For endogenous metabolites without labeled structural analogues, an automated algorithm selects the optimal internal standard for quantitation based on the rule of minimal coefficients of variations (COVs) after normalization ^6^.

FFAs Analysis: FFA analysis was conducted at LipidALL Technologies as previously described ^7^. FFAs were extracted from human cells using a modified version of the Bligh and Dyer’s method as described previously ^7^. Briefly, cells were homogenized in 750 µL of chloroform: methanol 1:2 (v/v) with 10 % deionized water and incubated at 4 ℃ for 30 min. At the end of the incubation, 350 µL of deionized water and 250 µL of chloroform were added. The samples were then centrifuged and the lower organic phase containing lipids was extracted into a clean tube. Lipid extraction was carried out twice and the lipid extracts were pooled into a single tube and dried in the Speed Vac under OH mode. Samples were stored at -80 ℃ until further analysis. FFAs were analyzed using Shimadzu Nexera20AD-HPLC coupled with triple quadrupole/ion trap mass spectrometer (6500 Plus QTRAP; SCIEX) as described previously ^8^. Lipids were separated by normal phase (NP)-HPLC was carried out using a TUP-HB silica column (internal diameter 150 × 2.0 mm, 3 µm) with the following conditions: mobile phase A (chloroform: methanol: ammonium hydroxide, 89.5:10:0.5) and mobile phase B (chloroform: methanol: ammonium hydroxide: water, 55:39:0.5:5.5). Free fatty acids were quantitated using d31-16:0 (Sigma-Aldrich) and d8-20:4 (Cayman Chemicals) as internal standards.

High-Resolution Metabolic Flux Analysis: Untargeted metabolic flux analysis was conducted at LipidALL Technologies as described previously ^9^. Polar metabolites were extracted from cells using 1mL of ice-cold methanol: H_2_O (4:1/ v:v) containing 0.224 mM phenylhydrazine. Samples were incubated at 1500 rpm for 30 min at 4 ^o^C. Following the incubation, samples were kept at -20 ^o^C for 1h for derivatisation of alpha-keto acids. Then, samples were centrifuged for 10 min at 12 000 rpm at 4 ^o^C. Clean supernatant was transferred to a new tube and dried in a SpeedVac under H_2_O mode. Total protein content was determined from the dried pellet using the Pierce^®^ BCA Protein Assay Kit according to the manufacturer’s protocol. The dried extract was reconstituted in 5% acetonitrile in water prior to LC-MS analysis on an Agilent 1290 II UPLC coupled to Sciex 5600+ quadrupole-TOF MS. For reverse phase chromatography, polar metabolites were separated on a Waters ACQUITY HSS-T3 column (3.0 × 100 mm, 1.8 μm), while a Waters ACQUITY BEH Amide column (2.1 × 100 mm, 1.7 μm) was utilized for hydrophilic interaction liquid chromatography (HILIC). MS parameters for detection were: ESI source voltage negative ion mode −4.5 kV; vaporizer temperature, 500 °C; drying gas (N_2_) pressure, 50 psi; nebulizer gas (N_2_) pressure, 50 psi; curtain gas (N_2_) pressure, 35 psi; the scan range was m/z 60-800 ^6^. Information-dependent acquisition mode was used for MS/MS analyses of the metabolites. Collision energy was set at -20 eV and -35 eV separately. Data acquisition and processing were performed using Analyst^®^ TF 1.7.1 Software (AB Sciex, Concord, ON, Canada). All detected ions were extracted using MarkerView 1.3 (AB Sciex, Concord, ON, Canada) into Excel in the format of 2D matrix, including mass to charge ratio (m/z), retention time and peak areas. PeakView 2.2 (AB Sciex, Concord, ON, Canada) was applied to extract MS/MS data and perform comparisons with the Metabolites database (AB Sciex, Concord, ON, Canada), HMDB and standard references to annotate ion identities ^6^. L-leucine-d_10_ was used as the internal standard to correct the endogenous metabolites in the samples and normalized them according to the amount of protein.

### *Sorbitol content detection*

Determine sorbitol content with a kit (G0560W, Grace Biotechnology). Collect cells in a centrifuge tube, centrifuge, and discard the supernatant. Resuspend pelleted cells (about 5 million) in 1 mL distilled water. Sonicate on ice (200 W, 3 s on, 10 s off, 30 cycles) to lyse cells. Heat at 95 °C for 10 min, cool, then centrifuge at 4 °C and 12000 rpm for 10 min. Take the supernatant and keep it on ice. Mix the assay reagent and sample in an EP tube, shake to develop color for 15 min. Centrifuge at RT and 12000 rpm for 5 min. Transfer 200 µL of the supernatant to a 96 - well plate and measure absorbance at 655 nm. Calculate actual sorbitol content using the standard curve.

### *Cysteine content detection*

Cysteine content was determined using a cysteine assay kit (A126-1-1, Nanjing Jiancheng Bioengineering) according to the manufacturer's instructions. Cells were collected into a centrifuge tube, centrifuged, and the supernatant was discarded. Cells (1 × 10⁴) were resuspended in Reagent 1 (0.2 mL) and sonicated on ice (20% power or 200 W, 3 s on, 10 s off, 30 cycles). The mixture was centrifuged at 8000 g and 4 °C for 10 min, and the supernatant was collected and kept on ice. The detection reagent and sample were added to an EP tube in sequence, mixed well, and incubated at RT for 15 min. The absorbance of each tube was measured at 600 nm (double distilled water was used to zero the instrument). The cysteine content in the samples was calculated based on the standard.

### *RNA sequencing assay*

Total RNA was extracted using TRIzol reagent. Two independent samples from each group (Huh-7 P, Huh-7 LR, and Huh-7 SR) were used for RNA sequencing, conducted by Biomarker Technologies (Beijing, China). Protein-protein interaction (PPI) networks for differentially expressed genes were predicted using the Search Tool for the Retrieval of Interacting Genes (STRING; http://string-db.org). Cytoscape bioinformatics software was used to visualize molecular interaction networks. The Molecular Complex Detection (MCODE) algorithm was applied to identify molecular complexes and densely connected regions within the PPI. Kyoto Encyclopedia of Genes and Genomes (KEGG) pathway analysis was conducted using OmicShare tools, an online bioinformatics platform (https://www.omicshare.com). Genes with median FPKM <1.0 across comparison groups were excluded from statistical evaluations to prevent artifactual interpretations.

### *Cellular energy metabolism analysis*

Cellular energy metabolism analysis was performed using the Seahorse metabolic analysis system. Cell suspensions (10,000 cells/80 μl per well) were seeded into Seahorse assay-specific culture plates, with the four corner wells (background wells) containing only medium without cells. The plates were left at room temperature for 1 hour and then incubated in a cell culture incubator for 24 h. 6 h prior to the experiment, the Seahorse instrument (Seahorse XFe96, Agilent) was activated to stabilize the temperature, and probe plates and assay media were prepared according to the protocol. The cell culture medium was replaced using a multichannel pipette, ensuring that each well contained 175 μl of medium. Experimental reagents, including glucose (G7528, Sigma), oligomycin (ab141829, Abcam), and 2-deoxyglucose (2-DG) (D8375, Sigma), were loaded into the drug injection ports of the probe plate and preheated to 37 °C. After the assay program was set up and probe calibration completed, the cell plate was placed in the Seahorse analyzer to initiate the experiment. The experiment was completed within 2 h, after which the data were processed and analyzed.

### *Nile red, Glutathione (GSH) and Reactive Oxygen Species (ROS) staining*

Nile red staining experiments were conducted by seeding 3,000 cells per well into black glass-bottom confocal 96-well plates. After 48 h of incubation, a staining solution was prepared in serum-free DMEM, containing 1 μM Nile red (neutral lipid probe) (N1142, Invitrogen), 1× CellMask (cell membrane probe) (C10046, Invitrogen), and 10 μM Hoechst 33342 (nuclear probe) (C1028, Beyotime Biotechnology). The cells were incubated at 37 °C for 30 min and then washed with PBS. After retaining 200 μl of medium, imaging was performed using a high-content imaging system, with fluorescence detection in the Alexa 568, Alexa 647, and Hoechst 33342 channels.

The staining procedures for GSH and ROS were similar to those for Nile red. The CellROX probe (ROS marker) (C10422, Invitrogen) required pre-treatment of cells with the IC_50_ concentration of the corresponding drug for 4 h prior to application, with a working concentration of 5 μM (detection channel: Alexa 647). The Monochlorobimane probe (GSH marker) (M1381MP, Invitrogen) was used at a working concentration of 50 μM (fluorescence wavelengths: λex = 394 nm, λem = 490 nm). Information on fluorescence probes is provided in **Supplementary Table 8**.

### *Measurement of NAD(H) and NADP(H) concentrations*

The levels of NAD(H) and NADP(H) in cells were measured using assay kits (Coenzyme I (NAD^+^+NADH) Content Colorimetric Assay Kit_A114-1-1 and Coenzyme Ⅱ (NADP^+^+NADPH) Content Colorimetric Assay Kit_A115-1-1, Nanjing Jiancheng Bioengineering). Samples were extracted for NAD⁺/NADP⁺ and NADH/NADPH using acidic and alkaline extraction solutions. NADH/NADPH reduced phenazine methosulfate, converting oxidized thiazolyl blue (MTT) to formazan, and absorbance was measured at 570 nm. Meanwhile, NAD⁺/NADP⁺ were reduced to NADH/NADPH using alcohol dehydrogenase and subsequently quantified via the MTT reduction assay.

NAD⁺ and NADH concentrations were calculated as follows:
NAD⁺ content (nmol/10⁴ cells) = [(Absorbance_measured − Absorbance_control − 0.099) × 36.1] / (500 / V₁);
NADH content (nmol/10⁴ cells) = [(Absorbance_measured − Absorbance_control − 0.065) × 24.7] / (500 / V₁).
Notes: V₁ represents the volume of extraction solution added (2 mL), and 500 represents the total cell count (5 million).

For NADP⁺ and NADPH quantification:
NADP⁺ content (nmol/10⁴ cells) = [4.57 × (Absorbance_measured − Absorbance_control − 0.062) × V₁] / (500 × V₁ / V₂);
NADPH content (nmol/10⁴ cells) = [7.2 × (Absorbance_measured − Absorbance_control − 0.072) × V₁] / (500 × V₁ / V₂).
Notes: V₁ represents the volume of sample added to the reaction system (0.05 mL), V₂ represents the volume of extraction solution added (2 mL), and 500 represents the total cell count (5 million).

### *Triglyceride detection*

The triglyceride content in cells and tumor tissues was measured using the Triglyceride Single Reagent GPO-PAP Method Assay Kit (A110-1-1, Nanjing Jiancheng Bioengineering). Briefly, after sample preparation, protein concentration was determined using the Omni-Easy^TM^ Ready-to-Use BCA Protein Quantification Assay Kit (ZJ102, Epizyme Biotech). The working solution for triglyceride measurement was then added to the blank control wells, standard wells, and sample wells, followed by incubation at 37 °C for 10 min. Absorbance at 510 nm was measured with a multifunctional microplate reader.

The triglyceride content (mmol/g tissue) was calculated using the following formula:
Triglyceride content = [(Absorbance_sample − Absorbance_blank) / (Absorbance_standard − Absorbance_blank)] × standard concentration (2.26 mmol/L) / sample protein concentration (g protein/L).

### *Raman spectroscopy and stimulated Raman scattering (SRS) imaging*

Raman spectroscopy and SRS imaging were performed on cells after digestion and culture in confocal dishes at a density of 300,000 cells per dish for 48 h. The medium was replaced with PBS before the dishes were placed on the Raman confocal platform for spectral analysis. To assess the fatty acid uptake capacity of the cells, a culture medium containing 50 μM d_31_-palmitic acid (16497, Cayman) was prepared 24 h in advance, and SRS imaging was used for detection.

The specific detection parameters were as follows: Raman spectroscopy: Excitation at 707 nm, 70 mW; exposure time of 60 seconds. ps-SRS imaging: Pump power of 50 mW; Stokes power of 300 mW; pixel dwell time of 10 μs; detection at 2850 cm⁻¹ (CH_2_), 3010 cm⁻¹ (=CH), and 2107 cm⁻¹ (CD).

### *Fluorescent probe tracing for fatty acid uptake*

Fluorescent probe tracing for fatty acid uptake was performed by digesting the cells and culturing them in black glass-bottom confocal 96-well plates at a density of 5,000 cells per well for 48 h. Subsequently, BODIPY™ 500/510 C1, C12 (the FFAs tracking probe) (D3823, Invitrogen) was prepared in the culture medium. High-content imaging was performed at 15 min intervals for a total duration of 7.5 h. Information on fluorescence probes is provided in **Supplementary Table 8**.

### *Detection of fatty acid β-oxidation*

(1) Mitochondrial extraction: The cellular fatty acid β-oxidation assay begins with the isolation of cellular mitochondria using the Cell Mitochondria Isolation Kit (C3601, Beyotime Biotechnology). Specifically, 20 million cells were collected, washed with PBS, and centrifuged to discard the supernatant. Mitochondrial isolation reagent containing PMSF was added, and the mixture was incubated on ice for 15 min. A Dounce homogenizer was used to homogenize the sample for 10 passes, and trypan blue staining was performed to evaluate the homogenate until the percentage of viable cells decreased to less than 50%. The homogenate was centrifuged at 600 g for 10 min at 4 °C, and the supernatant was transferred for subsequent centrifugation at 11,000 g for 10 min to isolate the mitochondria. The mitochondria were resuspended in mitochondrial storage solution and maintained on ice. The mitochondrial protein concentration was subsequently measured using the Omni-Easy™ Ready-to-Use BCA Protein Quantification Assay Kit (ZJ102, Epizyme Biotech).

(2) Mitochondrial β-Oxidation Rate Assay: The mitochondrial β-oxidation rate was quantified using the GENMED^®^ Fatty Acid β-Oxidation Rate Colorimetric Assay Kit (GMS50679, Genmed B.V.). The kit evaluates the rate of β-oxidation by detecting the reduction rate of ferricyanide, which captures electrons generated during the oxidation of the substrate palmitoyl carnitine. The reduction rate was assessed by measuring the time-dependent decrease in absorbance at a wavelength of 420 nm. Strict adherence to the reagent addition sequence and incubation conditions specified in the manufacturer's instructions is required. The formula for calculating the oxidation rate is as follows: [(Sample well value−Back ground well value)×0.2 (System volume in ml)]/0.5 (Sample protein amount in mg)×105 (Molar absorptivity)×5 (Reaction time in min) μmol ferricyanide reduced/min/mg.

### *Single-cell RNA sequencing*

Single-cell transcriptome sequencing was performed using Seekgene Biotechnology's SeekOne^®^ DD single-cell full-length transcriptome sequencing solution. The detection steps included cell preparation, nucleus preparation, single-cell RNA-seq library construction and sequencing, single-nuclei RNA sequencing library construction and sequencing, sequencing data quality control, single-cell RNA sequencing data processing, and clustering and visualization. Single-cell transcriptome analysis was conducted using the online analysis platform Seeksoul (www.seeksoul.online). Specifically, cell stemness analysis was performed using the CytoTRACE program, transcription factor analysis was conducted with the SCENIC program, pseudotime analysis was performed using the Monocle2 program, and enrichment analysis was carried out using GO gene sets.

### *Western Blotting*

The cells were lysed in lysis buffer (P0013B, Beyotime Biotechnology) supplemented with protease inhibitors (ST505, Beyotime Biotechnology) and phosphatase inhibitors (P1081, Beyotime Biotechnology). The lysates were sonicated for 30 seconds and centrifuged at 12,000 rpm for 10 min at 4 °C. Proteins were separated by 6–10% sodium dodecyl sulfate-polyacrylamide gel electrophoresis (SDS-PAGE), transferred to polyvinylidene difluoride (PVDF) membranes, and probed with primary antibodies and horseradish peroxidase (HRP)-conjugated anti-rabbit IgG antibodies (A0208 and A0216, Beyotime Biotechnology). Target proteins were visualized using enhanced chemiluminescence (ECL) HRP substrate (WBKLS0100, Millipore). Information on antibodies is provided in **Supplementary Table 7**.

### *AKR1B1-Enzyme-Linked immunosorbent assay Kit (ELISA) detection*

The levels of AKR1B1 protein in the serum of HCC patients and cell culture supernatants were measured using an ELISA kit (EH2164, FineTest). Briefly, reagents and samples were equilibrated to room temperature, and washing buffer was prepared. Biotin-labeled antibodies and Streptavidin-Avidin-Biotin Complex (SABC) working solution were prepared. Standards and samples were added to the microplate, incubated at 37 °C, and washed. Biotin-labeled antibodies and SABC were subsequently added, followed by an additional washing step. Subsequently, 3,3',5,5'-Tetramethylbenzidine (TMB) substrate was added, and the mixture was incubated in the dark. A stop solution was added, and absorbance at 450 nm was measured with a microplate reader. The concentration of the target protein in the samples was calculated based on the standard curve.

### *Preparation of conditioned medium (CM)*

DMEM complete medium containing 10% FBS and 1% penicillin-streptomycin was applied to cells grown to 60% confluency in culture dishes and incubated for 48 h. The cell culture supernatant was then collected and centrifuged (600 rpm, RT, 5 min) to remove dead cell debris. The subsequent lyophilization protocol was performed as follows: Pre-lyophilization preparation involved adding 1.25% (w/v) mannitol as a lyoprotectant, followed by rapid freezing at -80 °C for 12 h. The frozen samples were transferred to a vacuum freeze-dryer for primary drying: temperature was gradually increased from -40 °C to -20 °C over 24 h under 100 mTorr pressure. Secondary drying was conducted by raising the temperature from -20 °C to 25 °C while maintaining 100 mTorr pressure. Process endpoint was determined when the lyophilization curve indicated residual moisture content <3%. The lyophilized powder was hermetically sealed and stored at -80 °C. For use, the powder was reconstituted with DMEM medium at the original pre-lyophilization volume, followed by filtration through a 0.22 μm membrane. Strict aseptic techniques were maintained throughout all procedures. Further subject the above-mentioned conditioned medium (CM) to ultracentrifugation at 120,000×g (4 °C) for 70 min, and collect the supernatant as the exosome-depleted conditioned medium.

### *Exosome isolation and characterization of concentration and size distribution*

Exosome enrichment was performed using the EXODUS H-600 exosome purification device and the SA03 exosome separation and enrichment nano-chip from Shenzhen Huixin Bio. Technology Co., Ltd., while size distribution and concentration were analyzed using the NanoCoulter G nanoparticle analyzer and GX03 detection chip from Resuntech (Shenzhen) Co., Ltd. The experimental procedure involved centrifugation of cell supernatant samples at 12,000 rpm for 30 minutes, followed by filtration through a 0.22 μm membrane and transfer to a 50 mL centrifuge tube for preparation. The EXODUS chip was inserted into the designated slot, and the sample was loaded. The program parameters, including sample number and volume, were configured on the device interface, and the SA03 chip along with the cell culture medium separation protocol (Cell Culture Medium-SA03-Strong) was selected to initiate the automated exosome separation and purification process. Upon completion, exosomes retained in the chip were resuspended in 200 μL of PBS buffer under sterile conditions and transferred to an EP tube for subsequent size distribution and concentration analysis.

### *Cell probe tracking experiment*

The cell tracking probe CFDA SE (1 μM) (16231, Lumiprobe) and CellTracker^тм^ CM-Dil (5 μM) (C7000, Invitrogen) were used to label the resistant cells by incubating them for 30 min. After washing, the cells were digested into a suspension and co-cultured with unstained primary cells in confocal plates. After 48 h of incubation, exosomes were visualized using confocal microscopy. Additionally, IF staining of the cells and nuclear staining were performed as complementary analyses. Information on fluorescence probes is provided in **Supplementary Table 8**.

### *Statistical methods*

All experiments were conducted in triplicate unless otherwise specified. Error bars indicate standard deviations. Data are presented as the mean ± standard deviation (SD) from three independent measurements. Statistical analyses were conducted using GraphPad Prism 8.4.0 or Origin 9.0 software. The results of IF and cell or cell spheroid viability assays were analyzed using an unpaired independent sample t-test. The combination index (CI) values were used to quantify the interaction between two drugs (CI < 1, synergism; CI = 1, additive effect; CI > 1, antagonism). Quantitative resistant index calculated as IC_50_ (resistant) / IC_50_ (parental) ± SEM. A p-value of < 0.05 was considered statistically significant. *p < 0.05, **p < 0.01, ***p < 0.001, ****p < 0.0001.

**
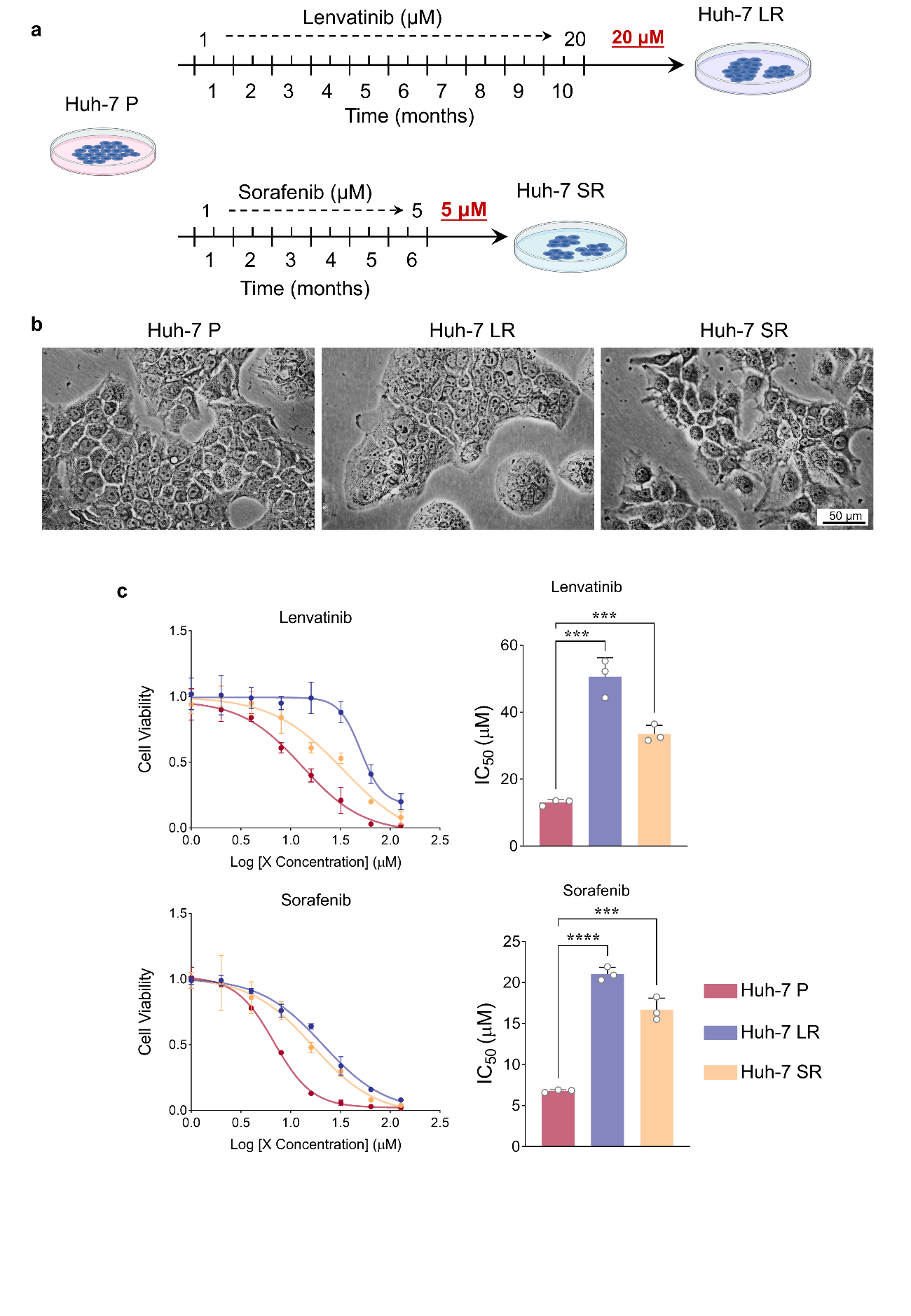
**

**Supplementary Fig. 1.** Construction and evaluation of drug-resistant cell models.

(a) Schematic representation of the drug-resistant cell model construction process. Schematic ﬁgures were generated with BioRender (https://app.biorender.com/). (b) The morphological changes of parental cell and drug-resistant cell models. Scale bar = 50 μm. (c) Cell viability assessment and IC_50_ values of parental and drug-resistant cells after treatment with Lenvatinib and Sorafenib respectively. Testing method: Unpaired Student’s t-test.

**
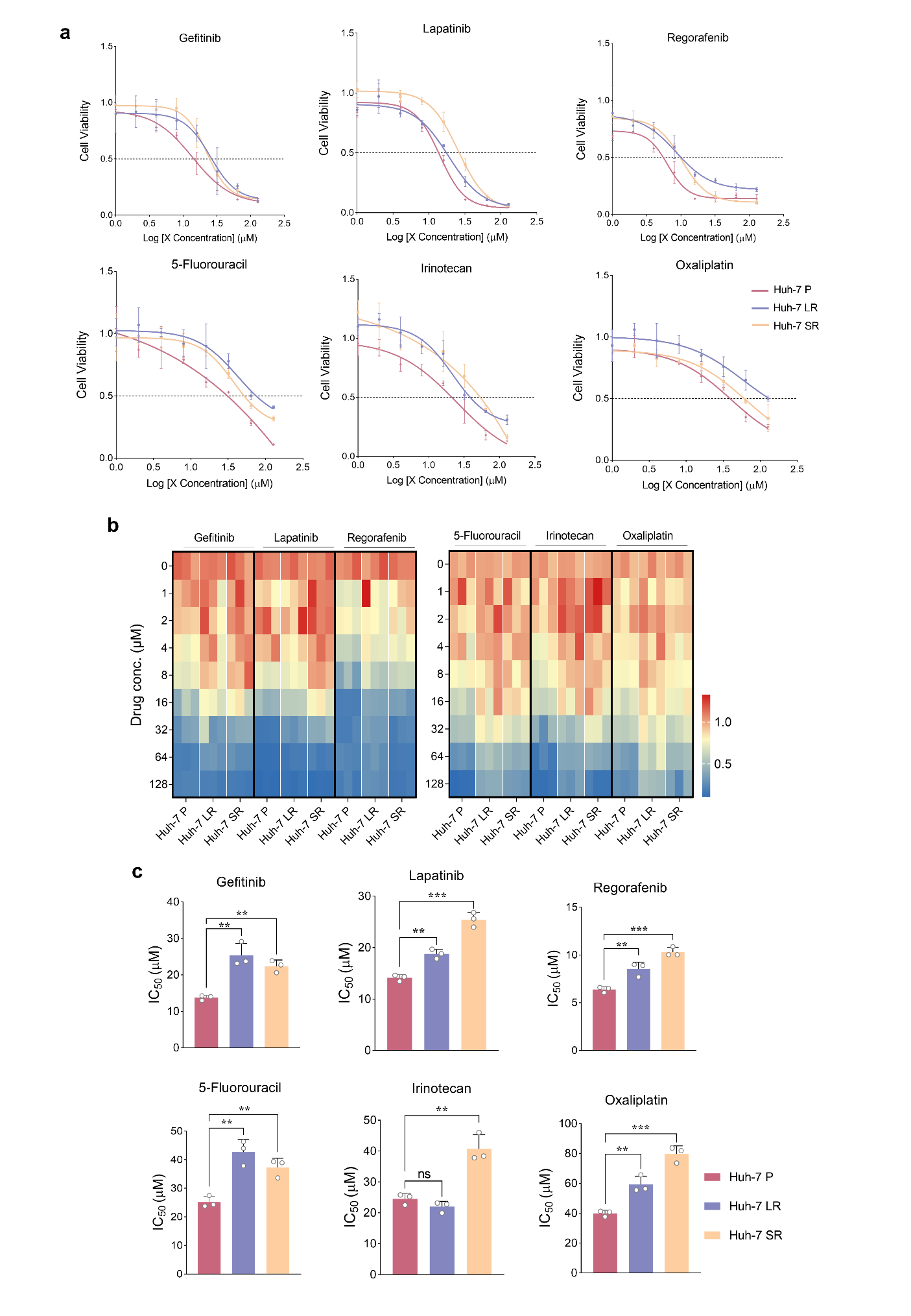
**

**Supplementary Fig. 2.** Evaluation of multidrug sensitivity in drug-resistant cells.

(a) Cell viability assessment of parental and drug-resistant cells after treatment with Gefitinib, Lapatinib, Regorafenib, 5-Fluorouracil, Irinotecan and Oxaliplatin respectively. (b) Heatmap of cell viability from (a). (c) IC_50_ values from (a). Testing method: Unpaired Student’s t-test.

**
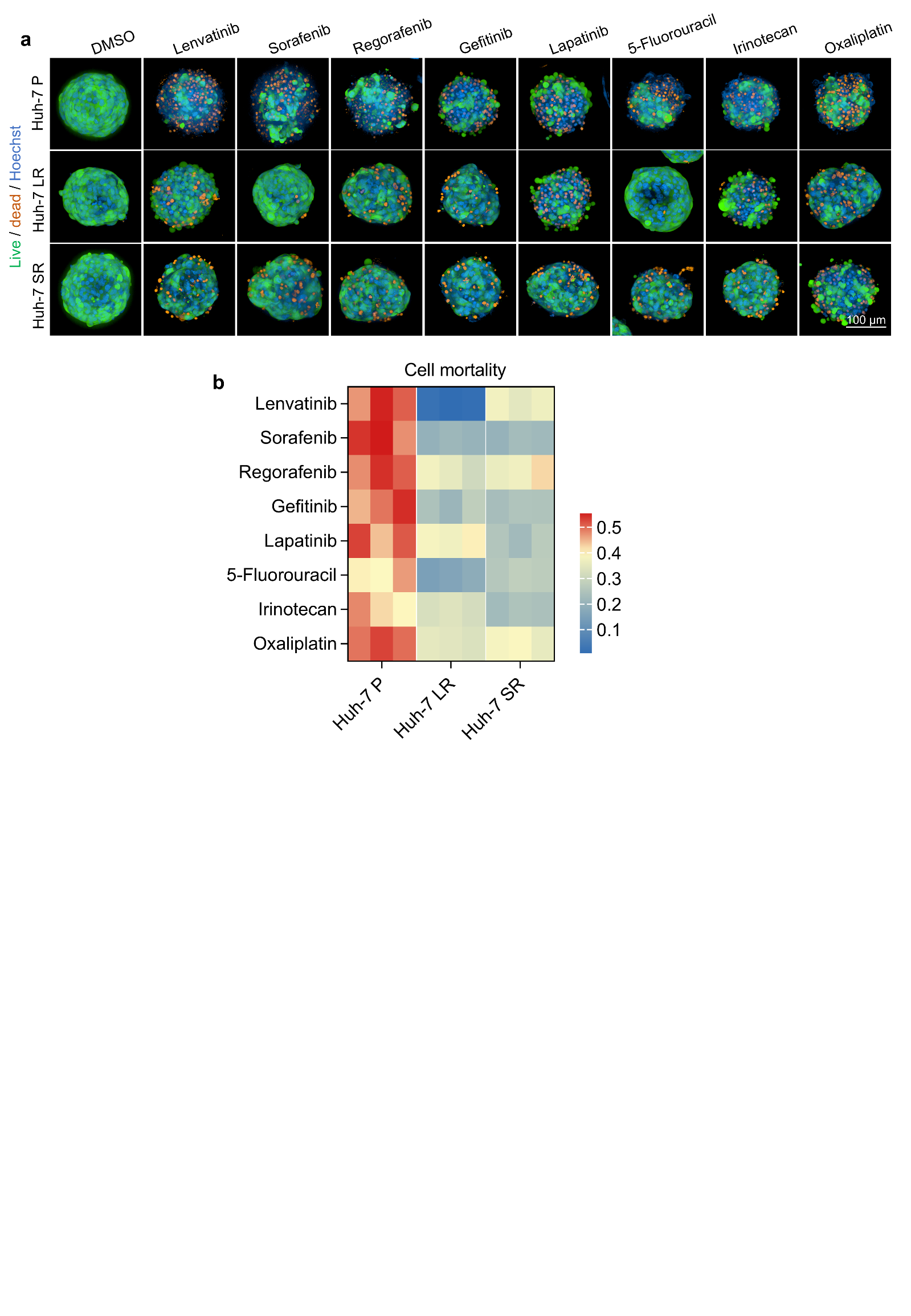
**

**Supplementary Fig. 3.** Evaluation of multidrug sensitivity in 3D drug-resistant microtissue models.

(a) Live/dead fluorescent probe assay for multidrug sensitivity in 3D microtissue models. Living cells (green), Dead dells (orange), DAPI (blue). Scale bar = 100 μm. (b) Heatmap of 3D microtissue models mortality statistics. The drug concentrations applied were at the IC_50_ values of the respective drugs for the parental cells.

**
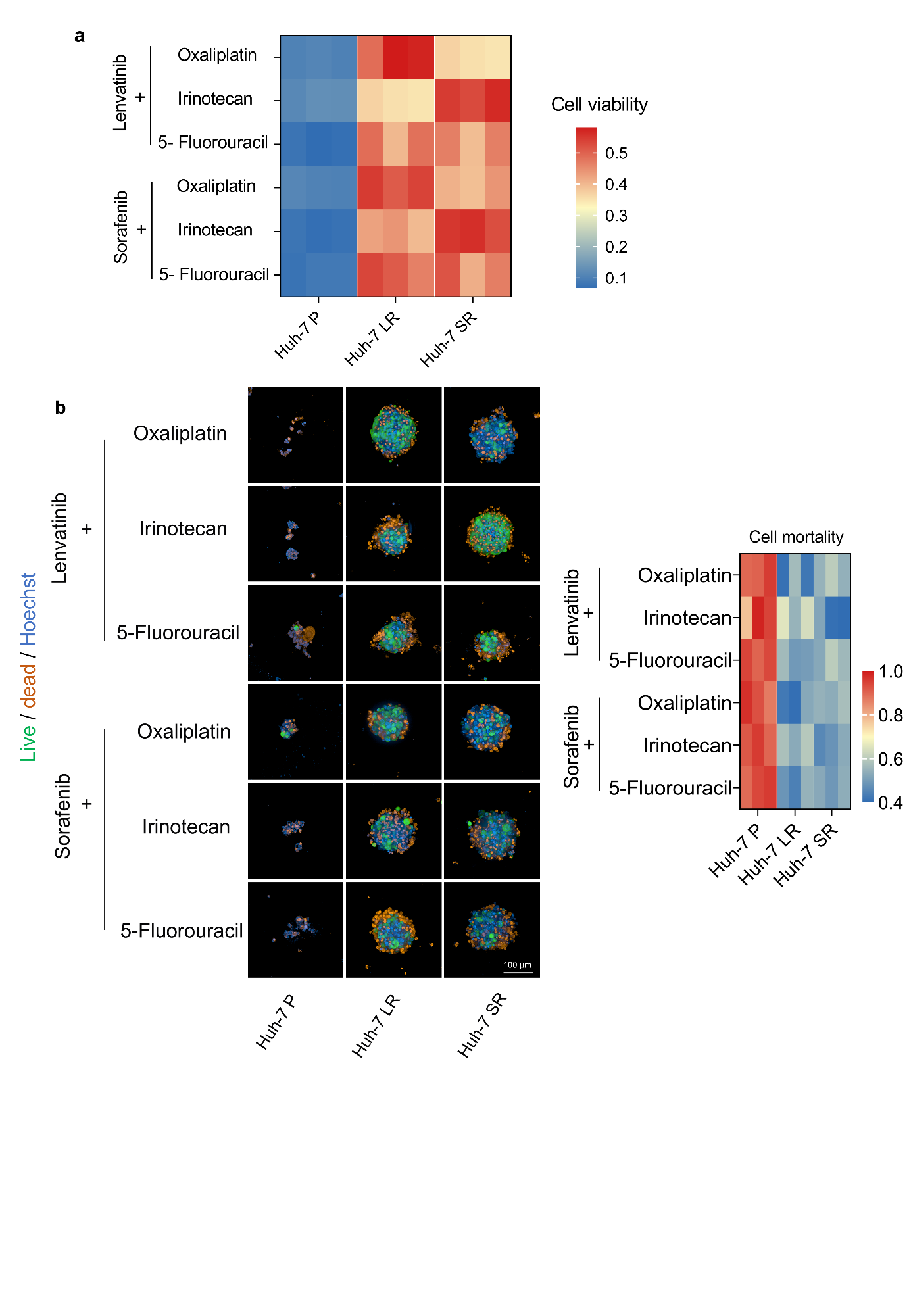
**

**Supplementary Fig. 4.** Evaluation of combined-drug sensitivity in drug-resistant cell and 3D microtissue models.

(a) Heatmap of combined-drug sensitivity in 2D cell lines. (b) Live/dead fluorescent probe assay for combined-drug sensitivity in 3D microtissue models and mortality statistics. Living cells (green), Dead dells (orange), DAPI (blue). Scale bar = 100 μm. The drug concentrations applied were at the IC_50_ values of the respective drugs for the parental cells.

**
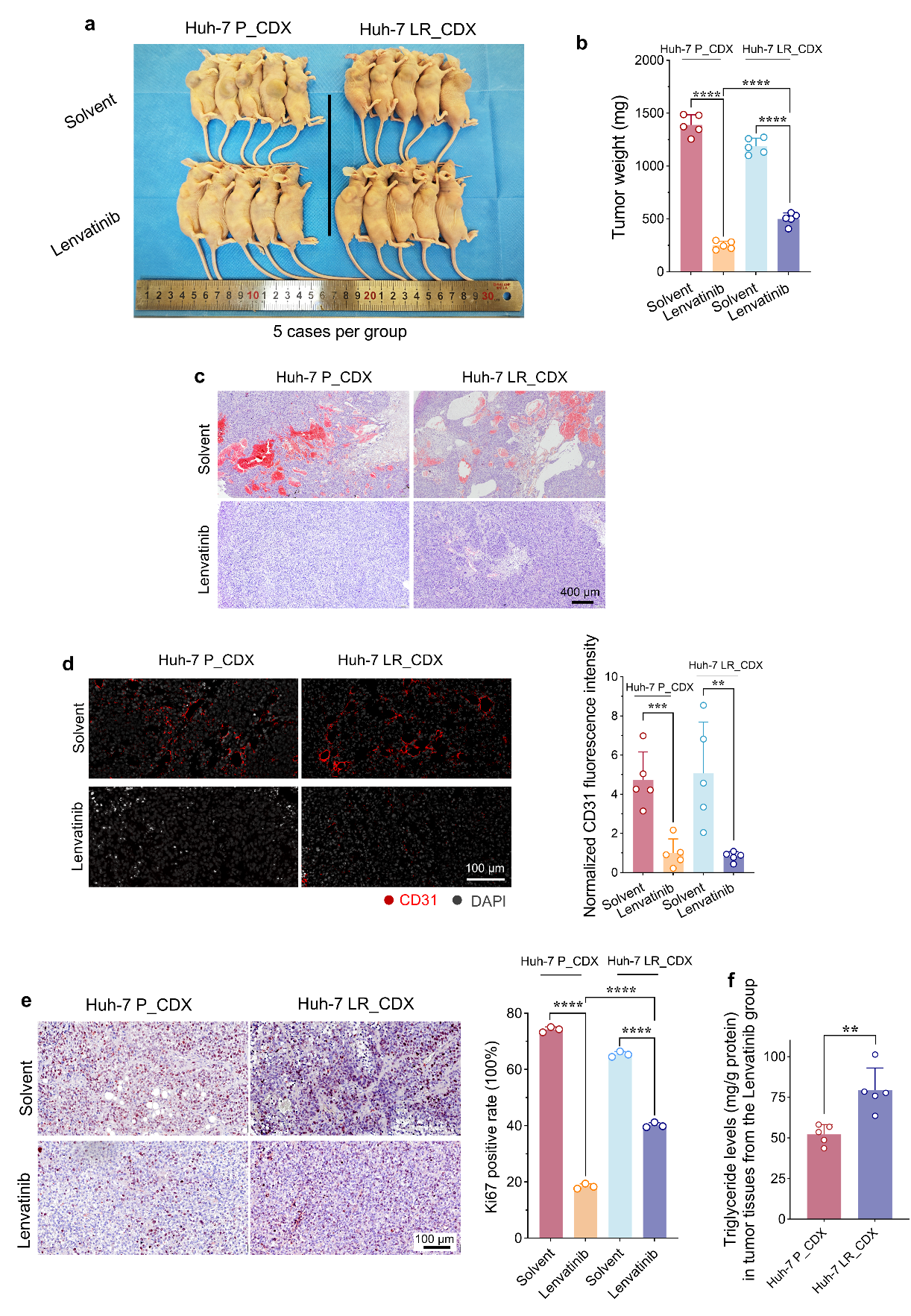
**

**Supplementary Fig. 5.** Evaluation of drug sensitivity in tumorigenic tissue of drug-resistant cells, along with assessment of cell proliferation and triglyceride content.

(a) Appearance of tumorigenic tissue from drug-resistant cells in nude mice (n=5/group). (b) Tumor weight statistics of tumorigenic tissue from drug-resistant cells. Testing method: Unpaired Student’s t-test. (c) H&E staining of tumorigenic tissue from drug-resistant cells. Scale bar = 400 μm. (d) IF staining image and statistics of CD31 (a vascular endothelium marker) in tumor tissues from CDX models of drug-resistant and parental cells. Scale bar = 100 μm. Testing method: Unpaired Student’s t-test. (e) Ki-67 IHC staining image and statistics of tumorigenic tissue from drug-resistant cells. Scale bar = 100 μm. Testing method: Unpaired Student’s t-test. (f) Triglyceride levels in tumor tissues from Lenvatinib-treated xenografts (n=5/group). Testing method: Unpaired Student’s t-test.

**
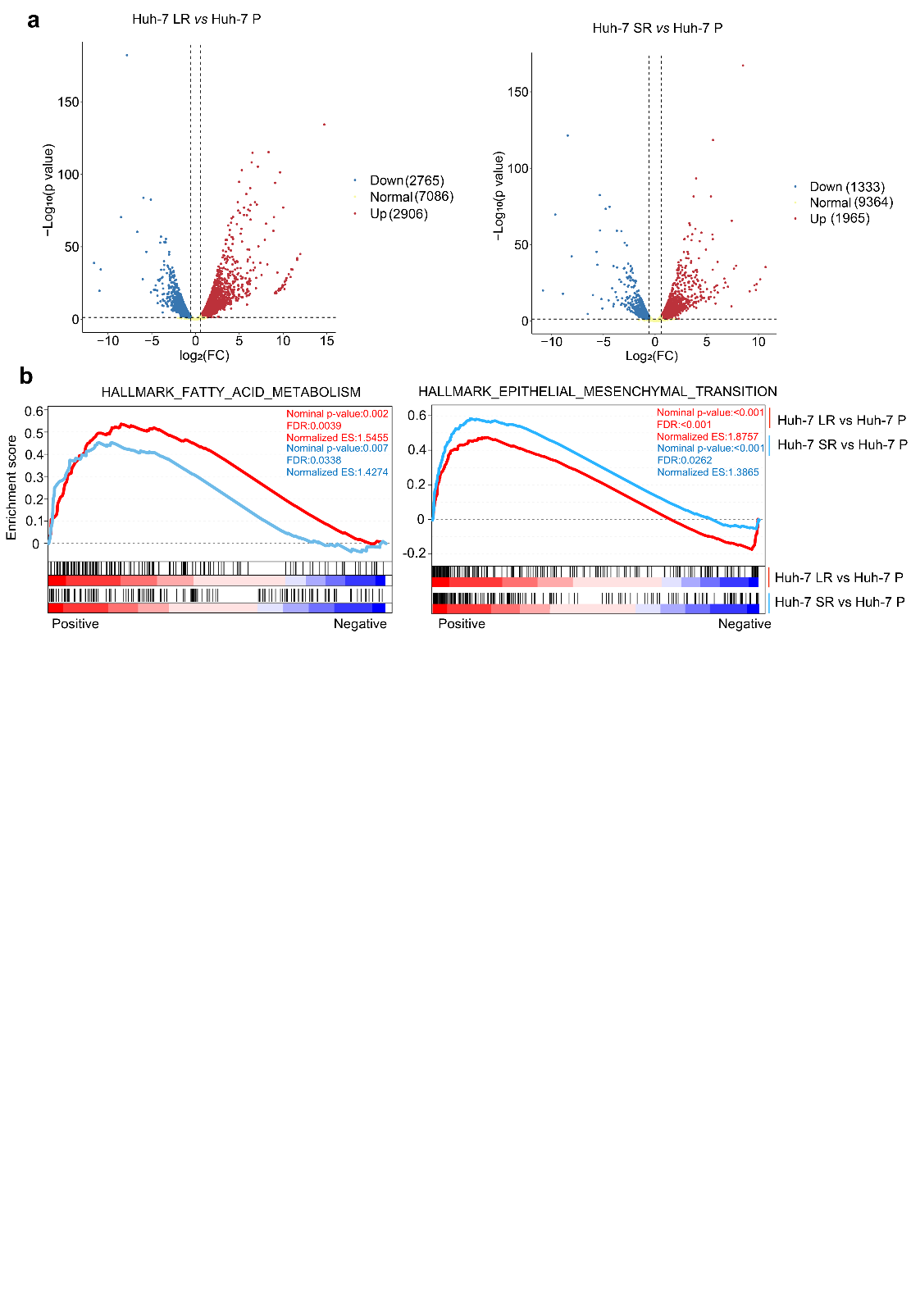
**

**Supplementary Fig. 6.** RNA-seq analysis of drug-resistant cells.

(a) Differential gene expression volcano plot of drug-resistant cells vs. parental cells. (b) GSEA focusing on fatty acid metabolism and EMT between drug-resistant cells and parental cells.

**
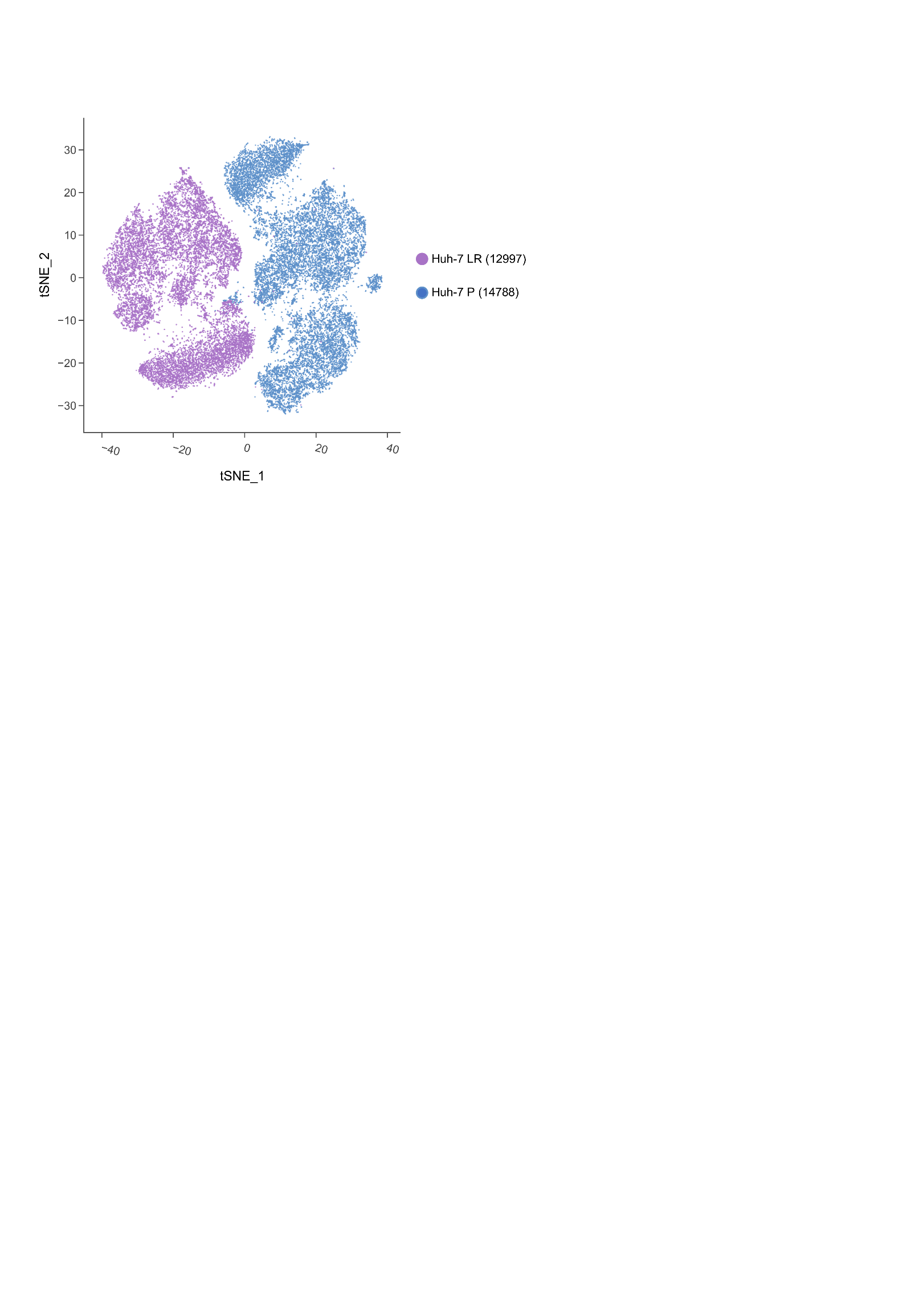
**

**Supplementary Fig. 7.** t-SNE plot of single-cell sequencing data distinguishing between drug-resistant cells and parental cells.

**
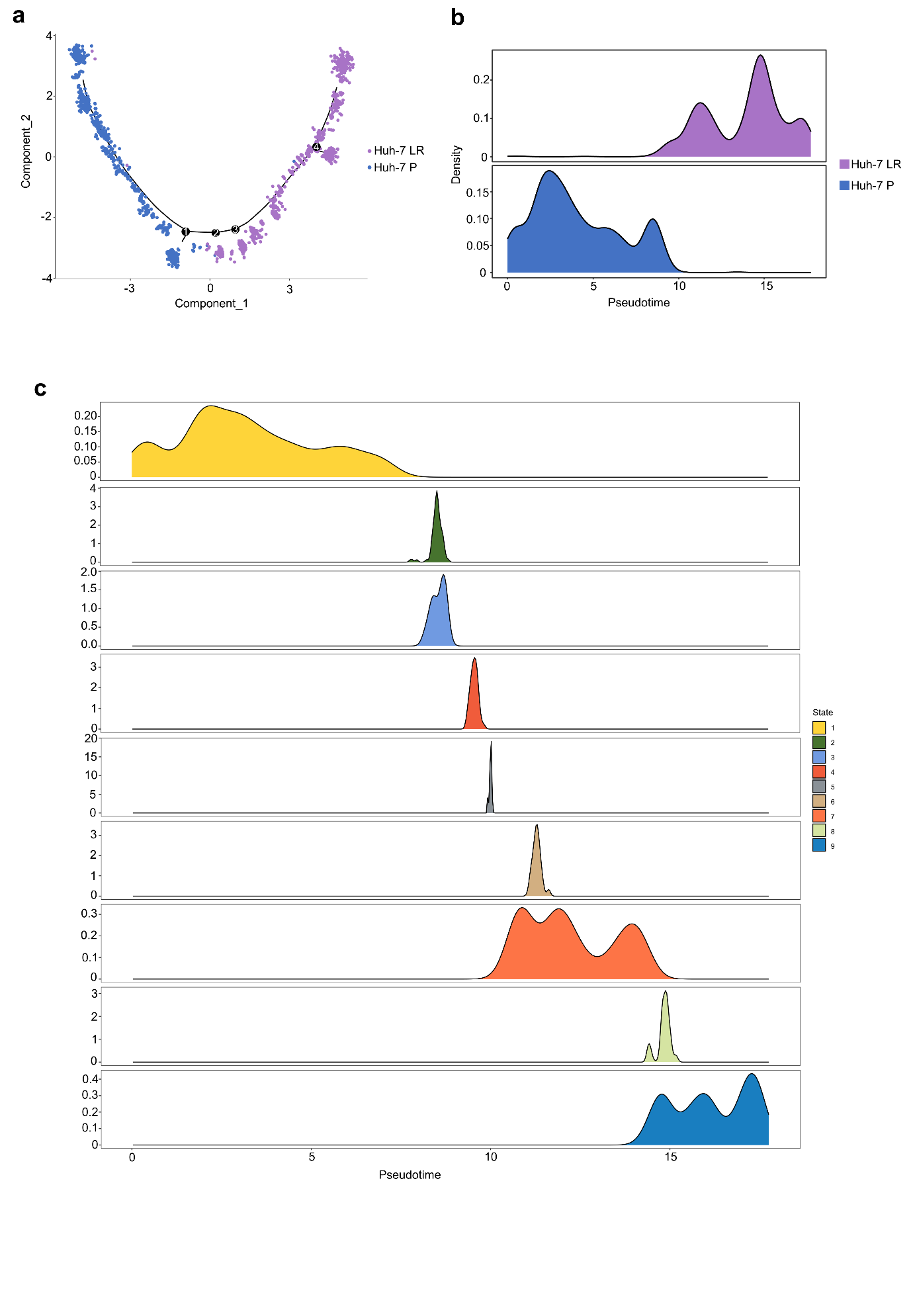
**

**Supplementary Fig. 8.** Pseudotime analysis of drug-resistant cells and parental cells.

(a) Pseudotime analysis with cell clustering by cell type. (b) Pseudotime analysis comparing parental cells and drug-resistant cells. (c) Pseudotime analysis of parental cells and drug-resistant cells after state assignment.

**
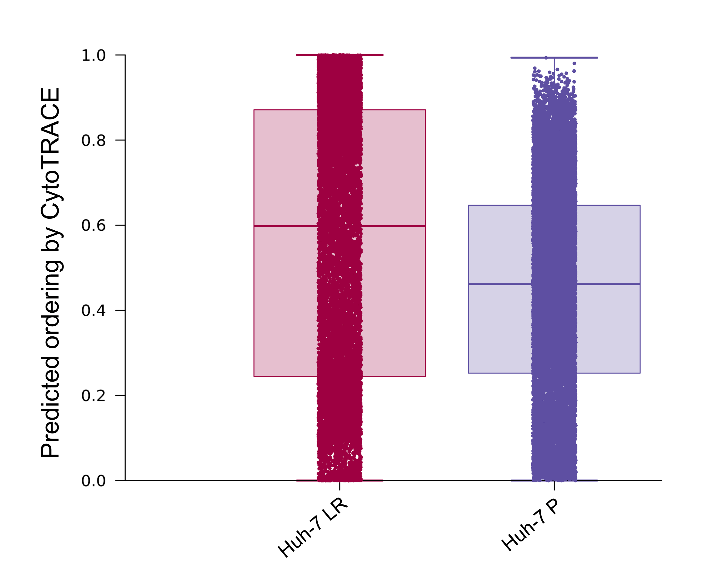
**

**Supplementary Fig. 9.** CytoTRACE analysis of stemness in drug-resistant cells and parental cells.

**
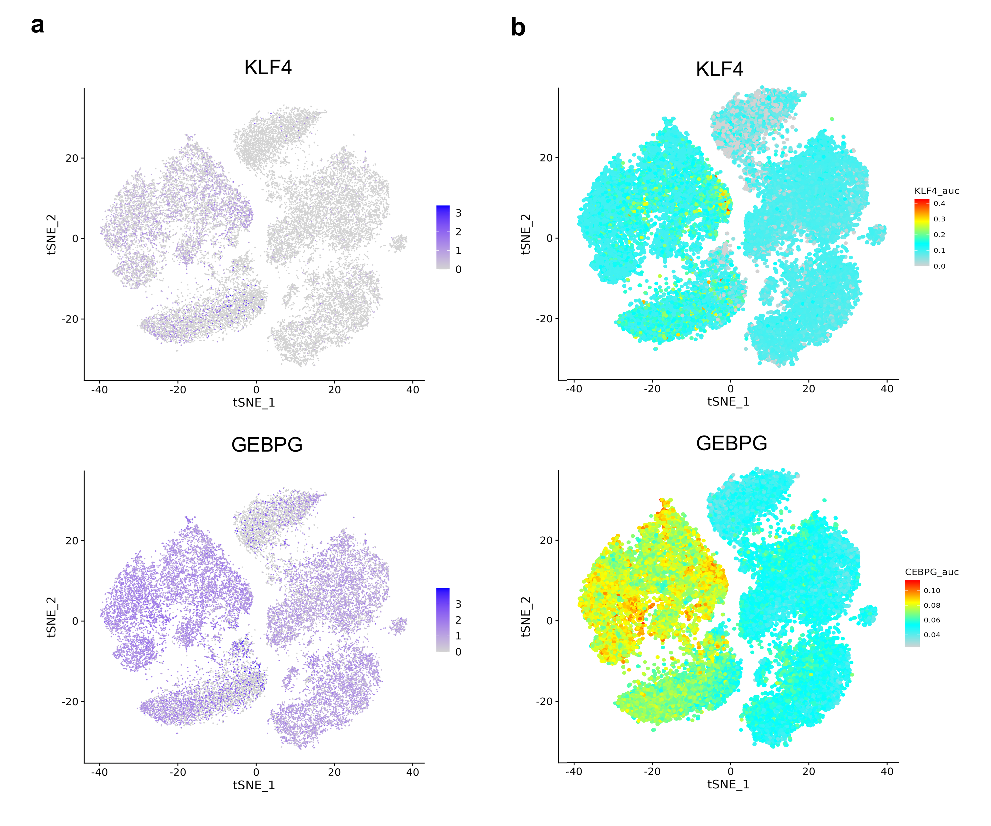
**

**Supplementary Fig. 10.** Transcription factor expression analysis of drug-resistant cells with the "on" state.

(a) Featureplot showing the expression of KLF4 and CEBPG regulons across drug-resistant cell and parental cell clusters. (b) Residual sum of squares calculated and AUC values determined.

**
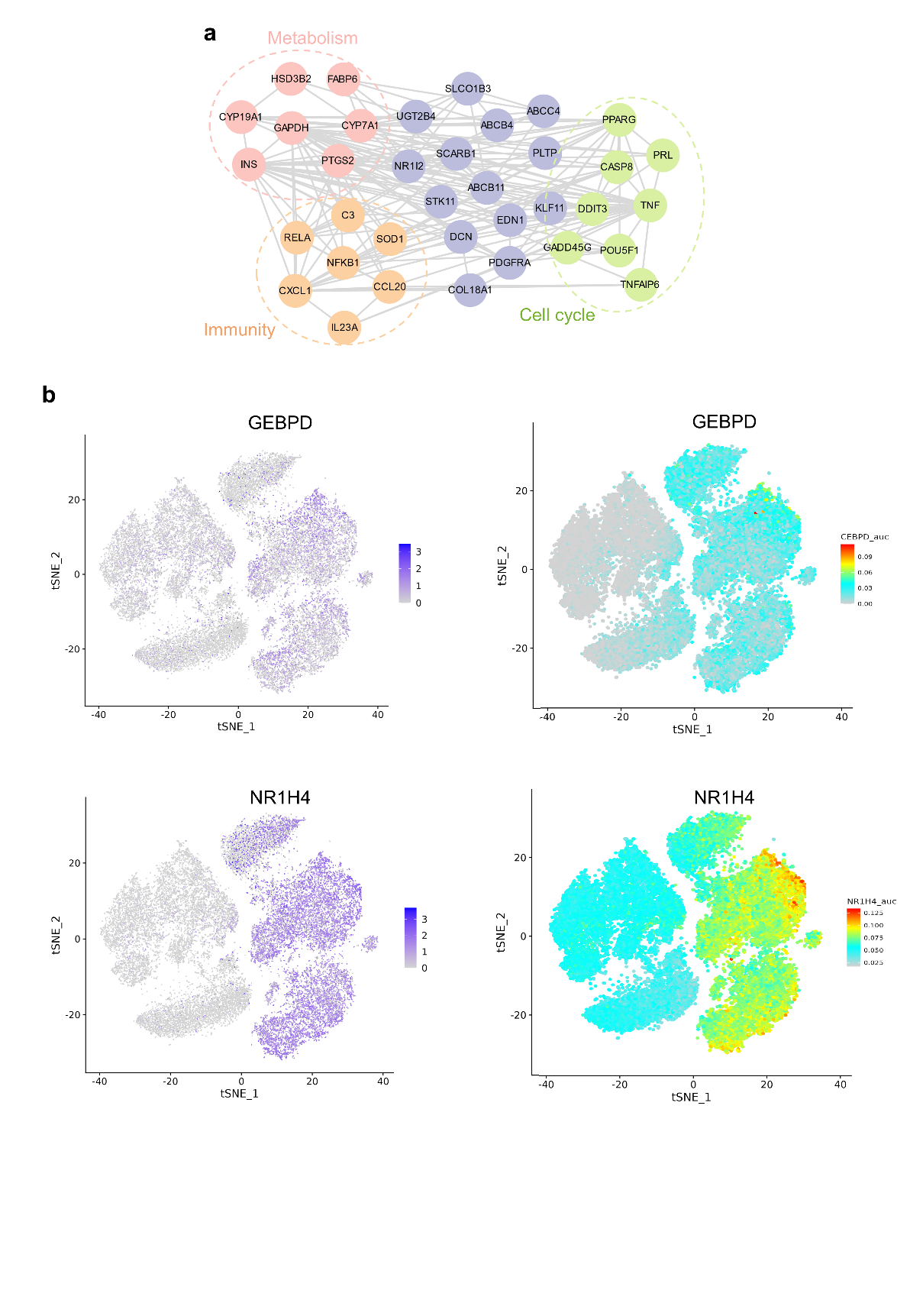
**

**Supplementary Fig. 11.** Transcription factor expression analysis of parental cells with the "on" state.

(a) PPI network analysis of downstream target gene clusters of transcription factors CEBPD and NR1H4. (b) Featureplot showing the expression of CEBPD and NR1H4 regulons across drug-resistant cell and parental cell clusters, with their residual sum of squares calculated and AUC values determined.

**
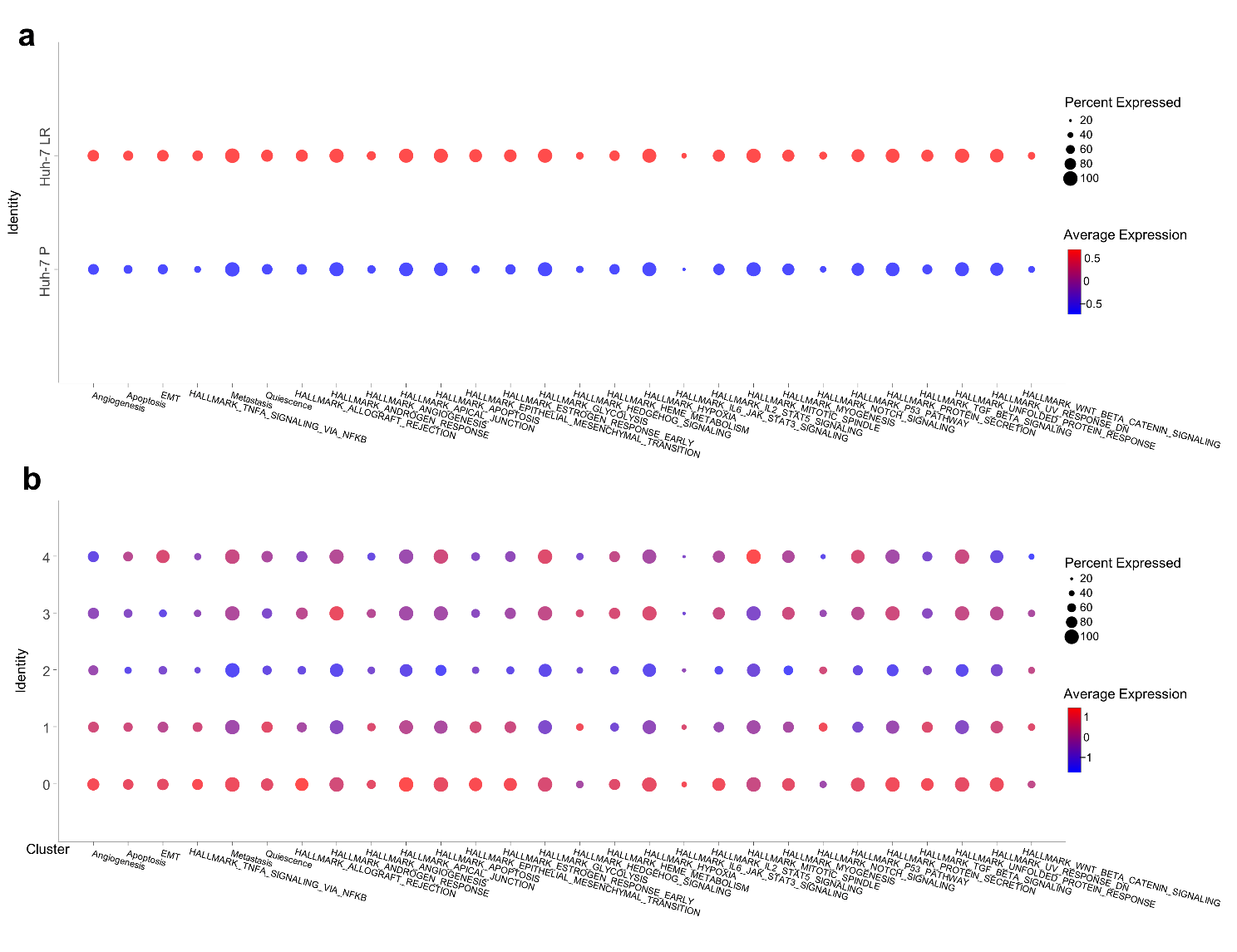
**

**Supplementary Fig. 12.** Cluster-based GSEA from single-cell sequencing data.

(a) GSEA of parental cells and drug-resistant cells. (b) GSEA of clustered cells.

**
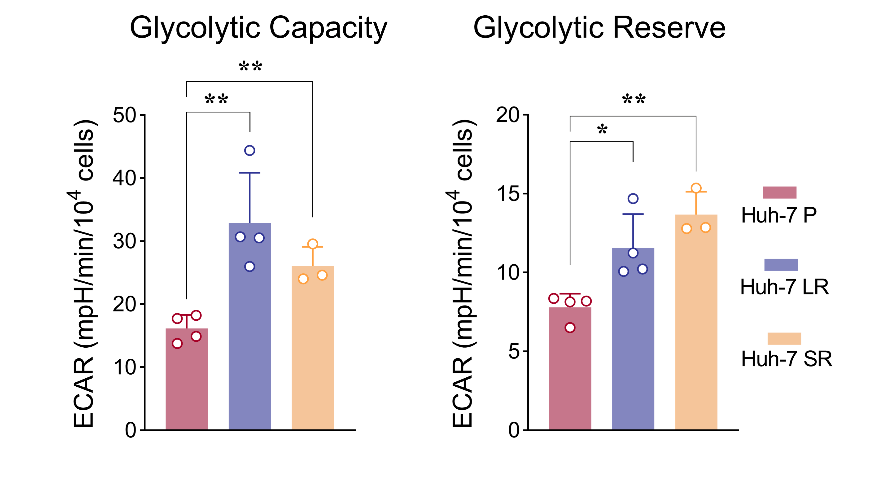
**

**Supplementary Fig. 13.** Seahorse energy metabolism analysis of cellular glycolytic capacity and glycolytic reserve. Testing method: Unpaired Student’s t-test.

**
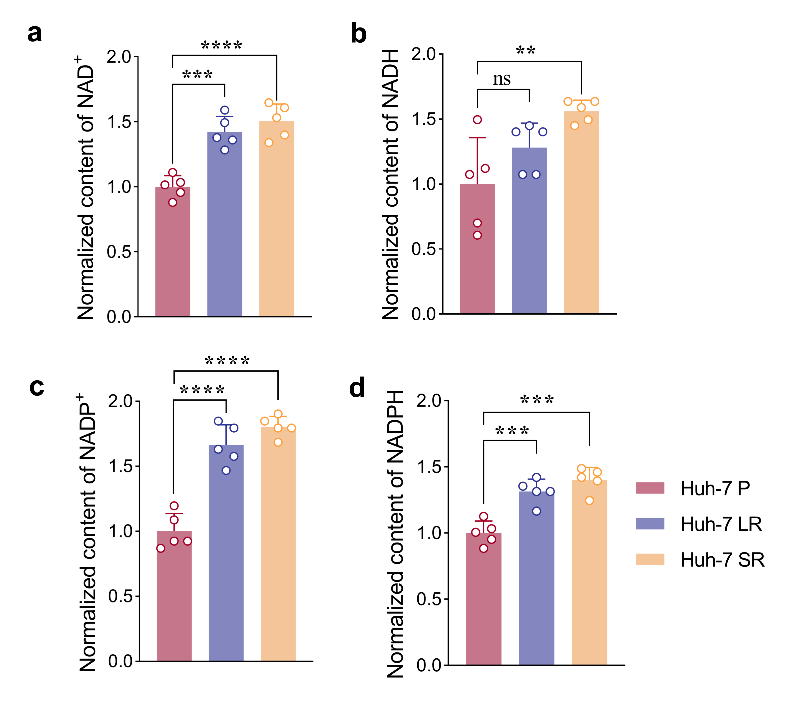
**

**Supplementary Fig. 14.** Detection of key enzyme levels in cellular redox reactions.

(a-b) Detection of intracellular NAD^+^ (a) and NADH (b) levels. (c-d) Detection of intracellular NADP^+^ (c) and NADPH (d) levels. Testing method: Unpaired Student’s t-test.

**
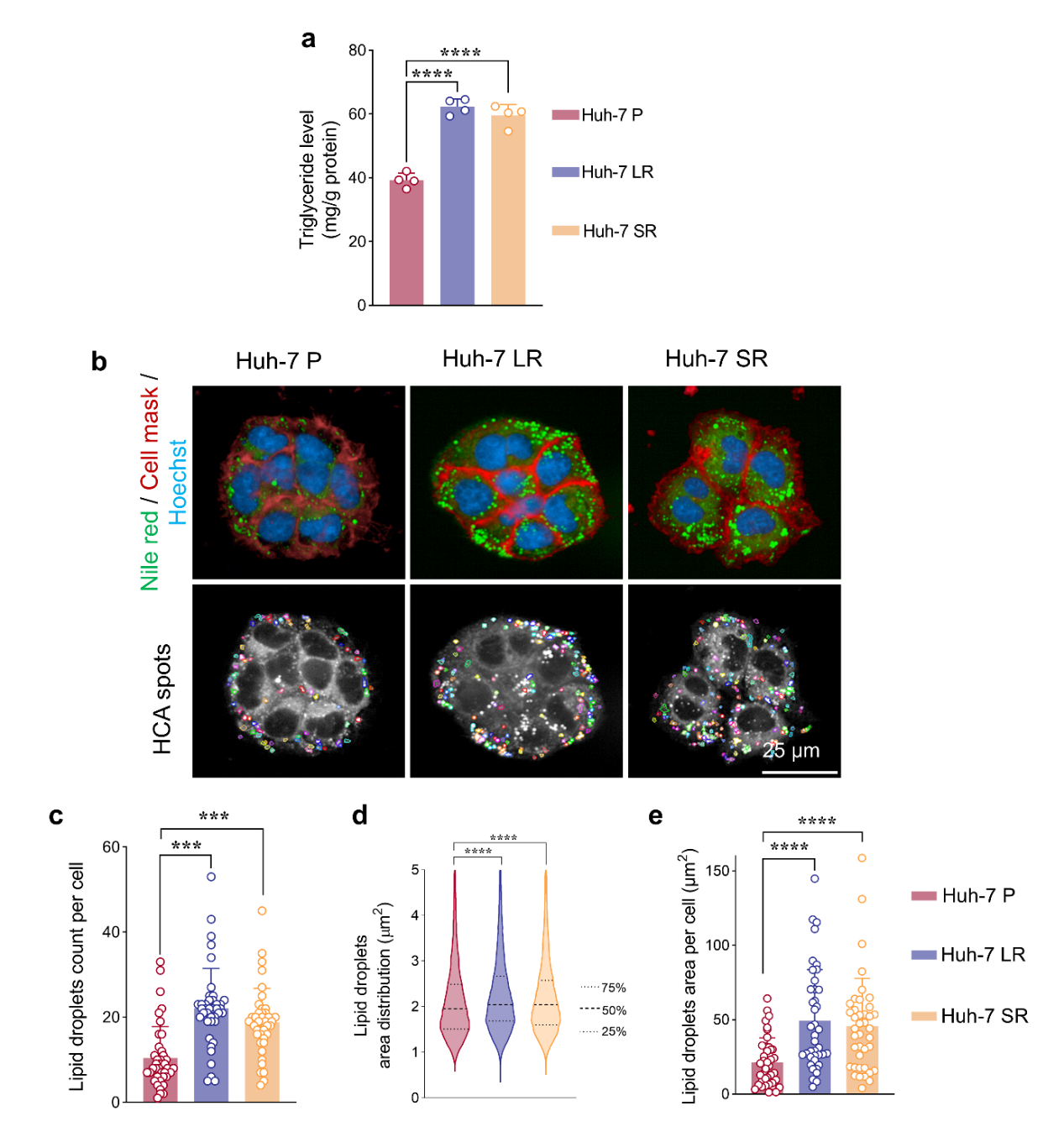
**

**Supplementary Fig. 15.** Lipid content assessment in drug-resistant cells.

(a) Measurement of cellular triglyceride levels. Testing method: Unpaired Student’s t-test. (b) Neutral lipid probe Nile Red detection of intracellular lipid droplet levels. Lipid droplets (green), cell membrane (red), nucleus (blue). High-content analysis (HCA) automatically recognizes lipid droplet content and distribution. Scale bar = 25 μm. (c) Quantification of individual cell lipid droplets count. Testing method: Unpaired Student’s t-test. (d) Statistical analysis of lipid droplet size distribution. Testing method: Unpaired Student’s t-test. (e) Quantification of individual cell lipid droplets area. Testing method: Unpaired Student’s t-test.

**
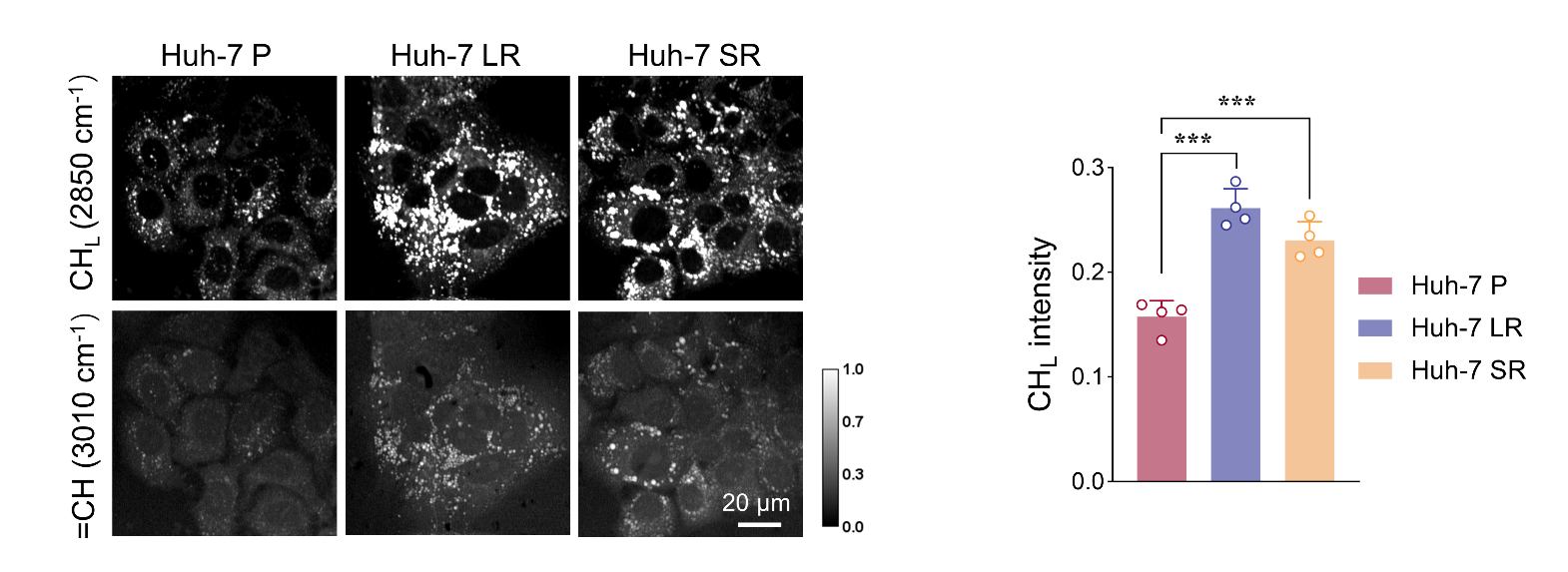
**

**Supplementary Fig. 16.** Raman spectroscopy detection and statistical analysis of lipid levels in drug-resistant cells. Scale bar = 20 μm. Testing method: Unpaired Student’s t-test.

**
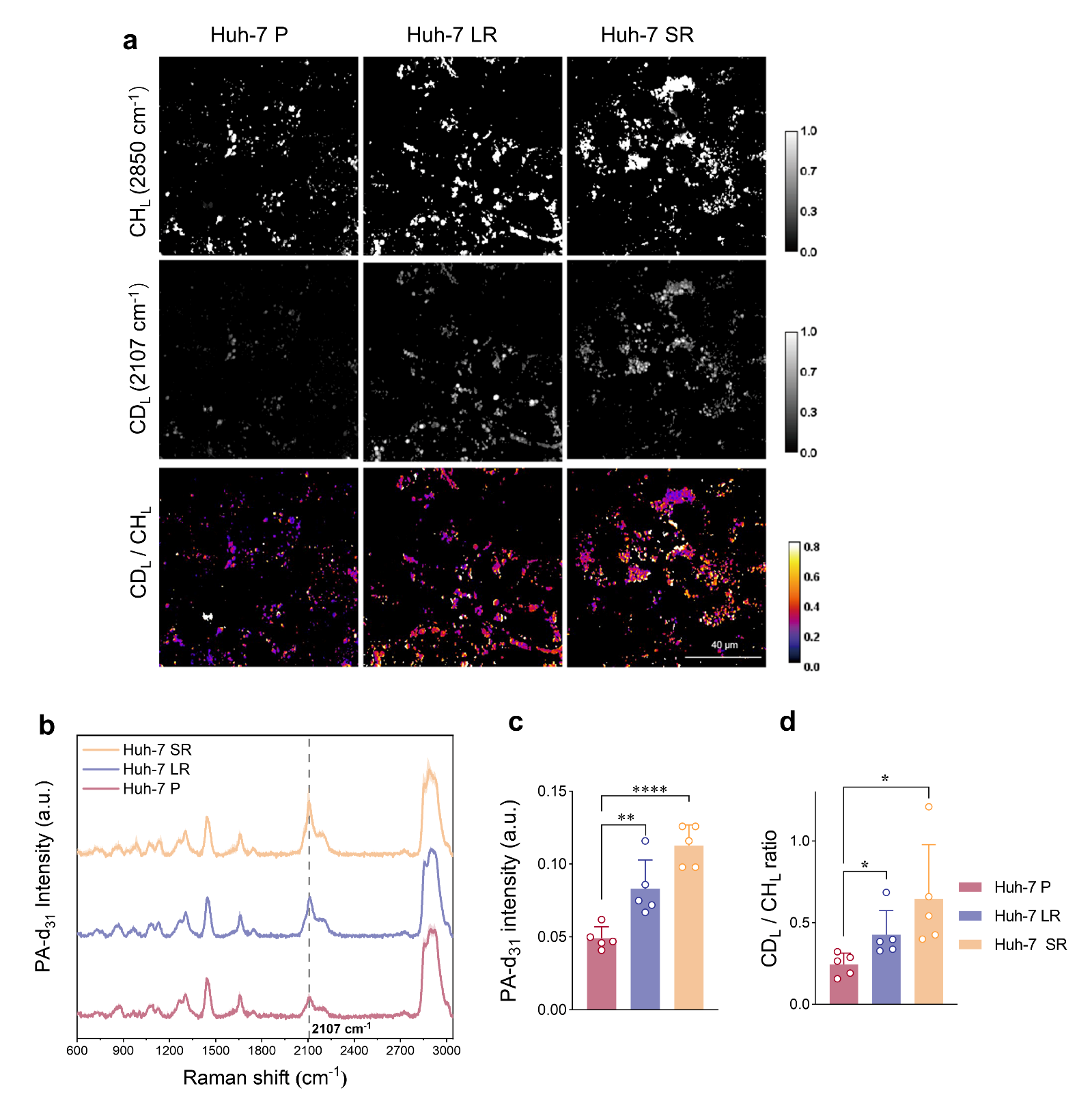
**

**Supplementary Fig. 17.** Stimulated Raman scattering (SRS) detection of cellular FFA uptake.

(a) SRS detection of palmitic acid (PA)-d_31_ uptake. Scale bar = 40 μm. (b) Intracellular PA-d_31_ intensity curve. (c) Statistical analysis of PA-d_31_ intensity at the 2107 cm^-1^ Raman shift in cells. Testing method: Unpaired Student’s t-test. (d) Statistical analysis of the ratio of taken-up PA-d_31_ to total lipids. Testing method: Unpaired Student’s t-test.

**
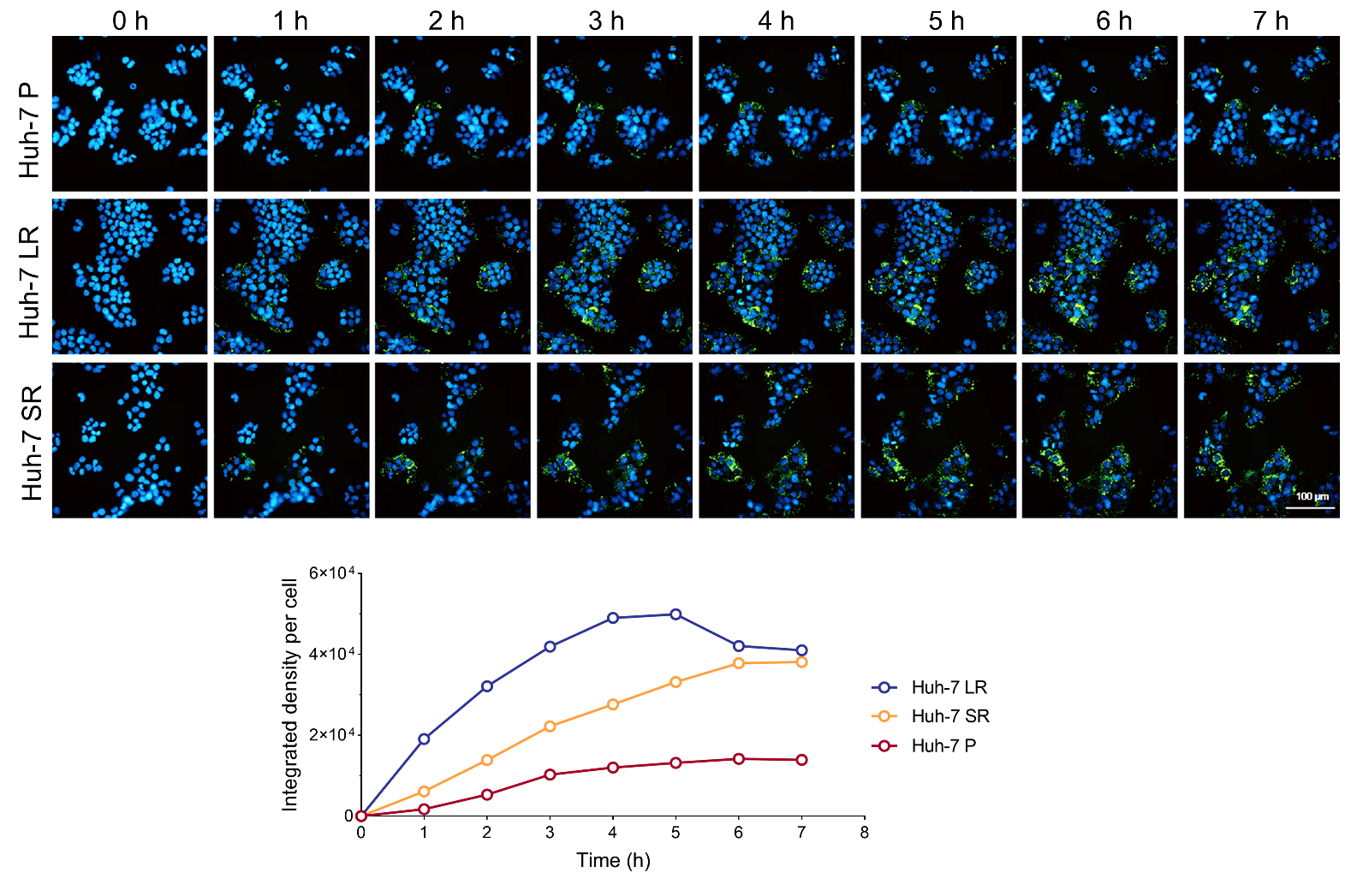
**

**Supplementary Fig. 18.** Detection of FFAs uptake efficiency in drug-resistant cells using the FFA analog tracing probe. FFAs analog (green), nucleus (blue). Scale bar = 100 μm.

**
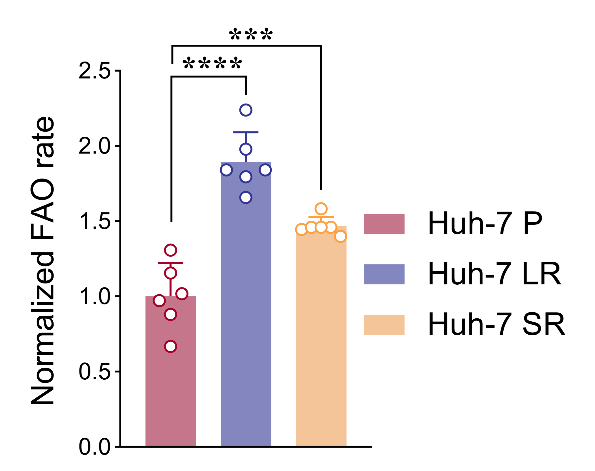
**

**Supplementary Fig. 19.** Detection of FFA β-oxidation (FAO) levels in drug-resistant cells. Testing method: Unpaired Student’s t-test.

**
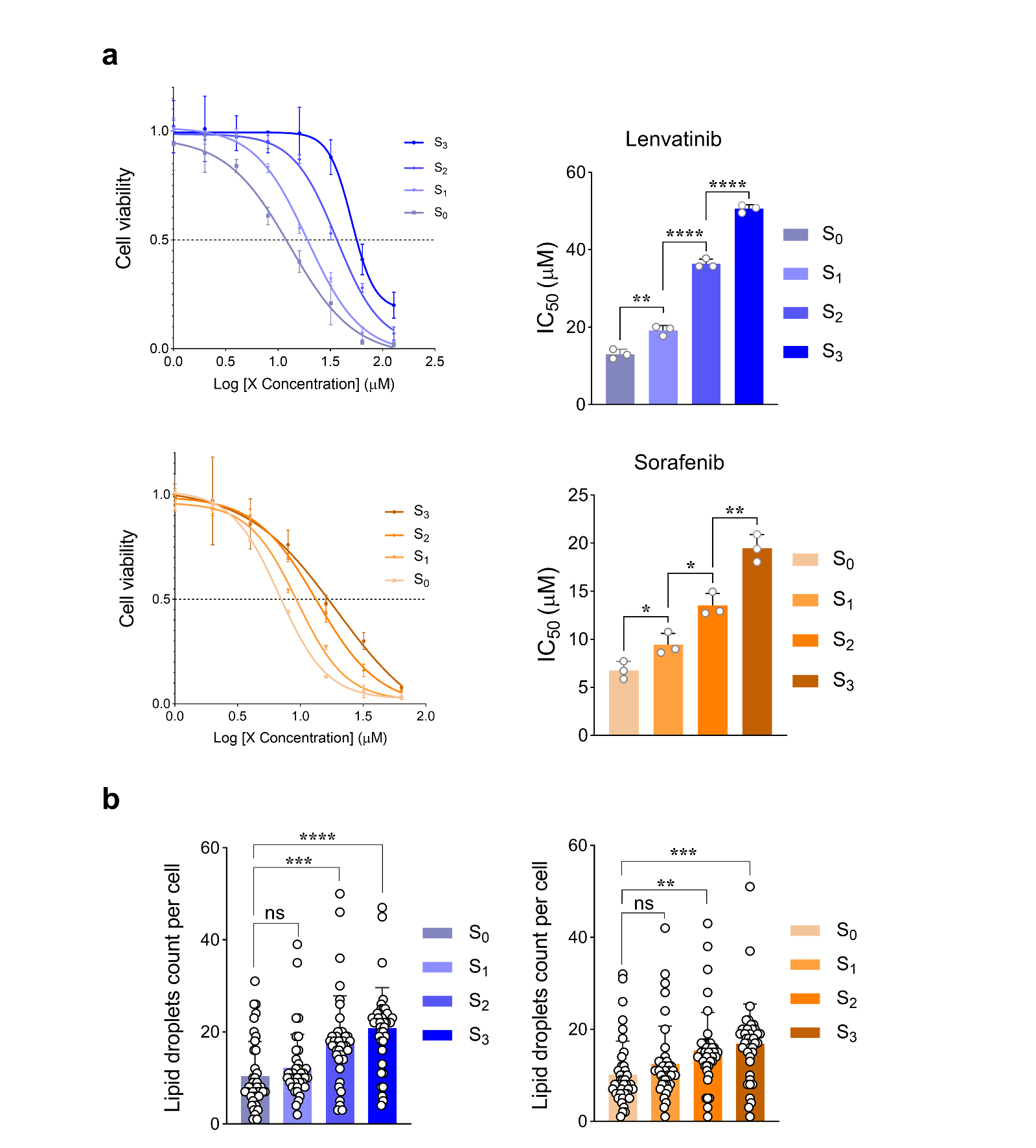
**

**Supplementary Fig. 20.** Assessment of drug sensitivity and lipid droplet levels in stage-specific drug-resistant cells.

(a) Detection of cellular activity and IC_50_ values of drug-resistant cells at various stages of construction. Testing method: Unpaired Student’s t-test. (b) Statistical analysis of lipid droplet content in stage-specific drug-resistant cells. Testing method: Unpaired Student’s t-test. (**p<0.01; ***p<0.001; ****p<0.0001).

**
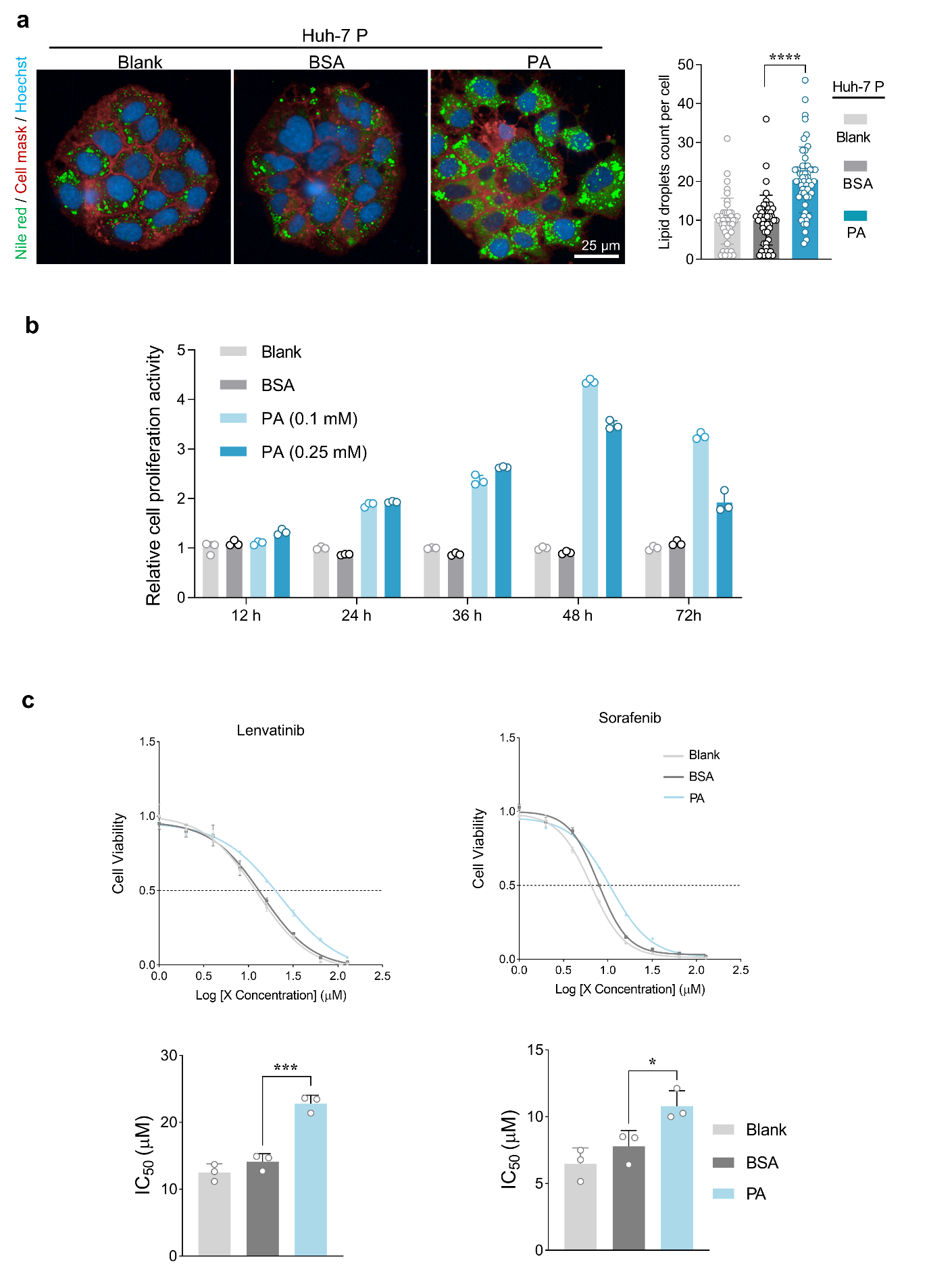
**

**Supplementary Fig. 21.** Correlation analysis between intracellular lipid droplet levels and drug sensitivity in drug-resistant cells.

(a) Nile red probe staining and quantification of artificially increased lipid droplets in parental cells. Lipid droplets (green), cell membrane (red), nucleus (blue). Scale bar = 25 μm. Testing method: Unpaired Student’s t-test. (b) The effect of Palmitic acid (PA) on the proliferation activity of parental cells. (c) Cellular activity curve of parental cells in response to PA (0.1 mM) and the corresponding IC_50_ value. Testing method: Unpaired Student’s t-test.

**
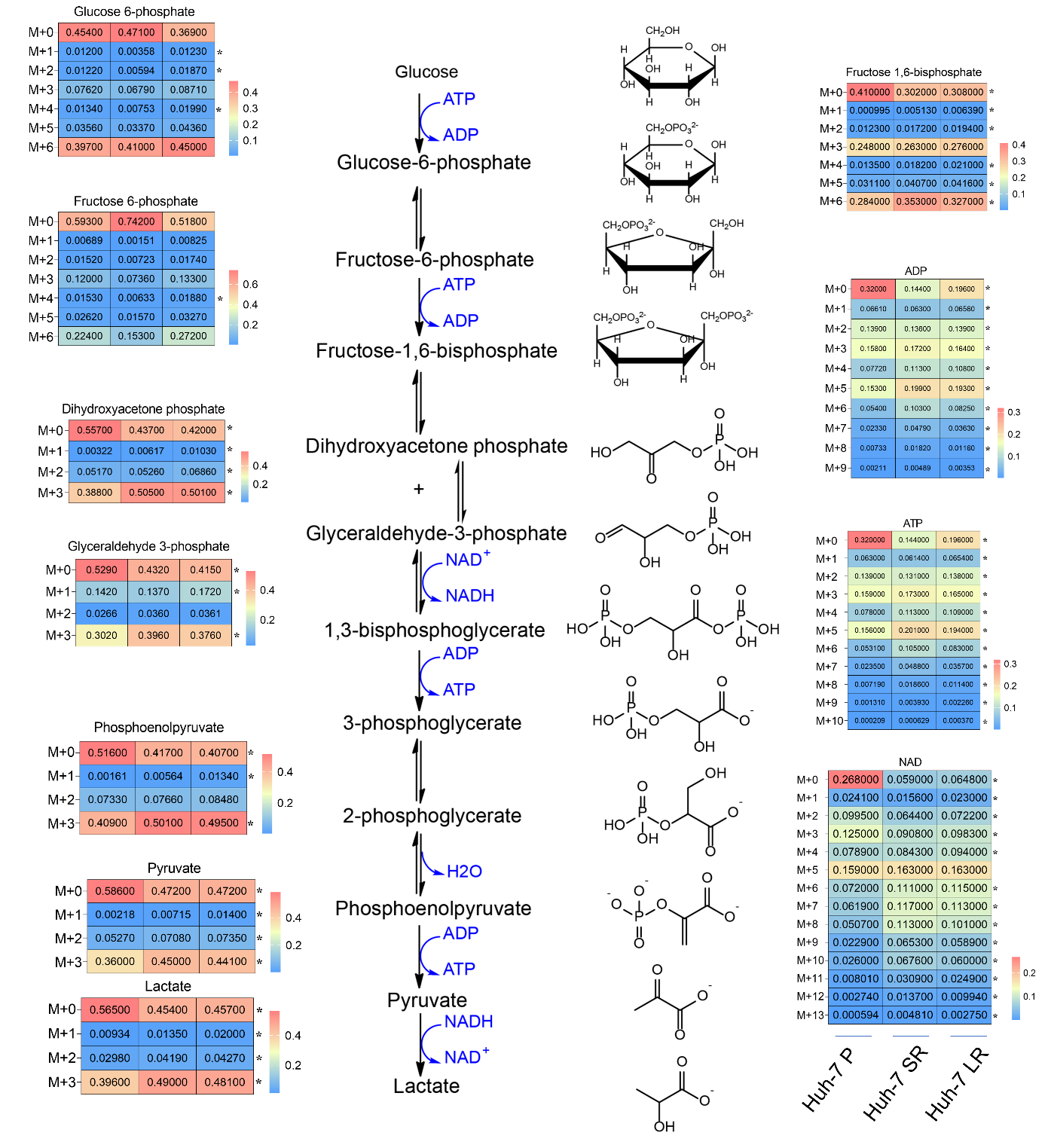
**

**Supplementary Fig. 22.** Intracellular glycolysis metabolic flux detection. “M+number” indicates the number of additional ¹³C atoms in the metabolite molecule. “*” indicates a significant difference between the corresponding substance in Huh-7 LR/SR and Huh-7 P, with p < 0.05.

**
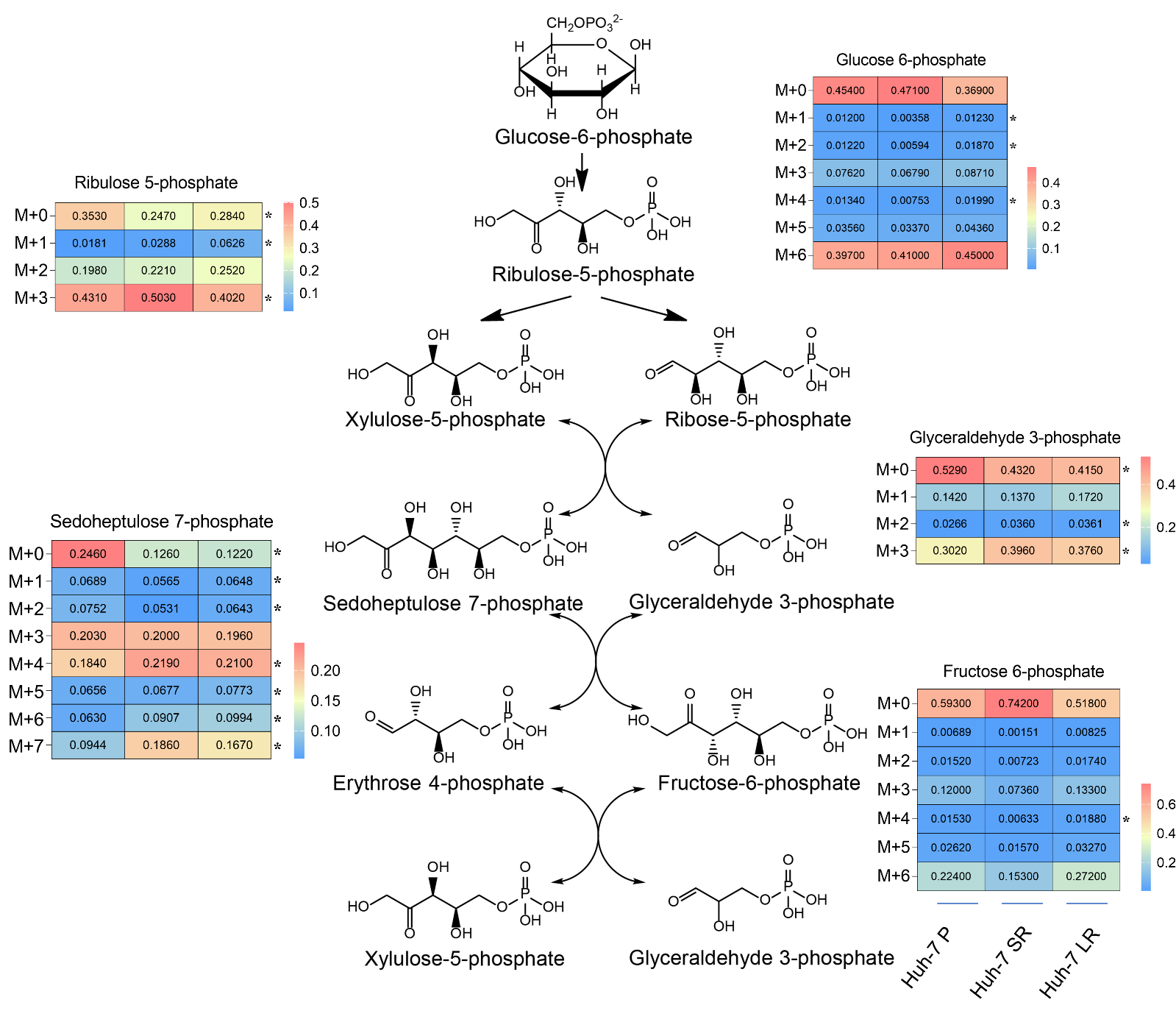
**

**Supplementary Fig. 23.** Intracellular pentose phosphate pathway metabolic flux detection. “M+number” indicates the number of additional ¹³C atoms in the metabolite molecule. “*” indicates a significant difference between the corresponding substance in Huh-7 LR/SR and Huh-7 P, with p < 0.05.

**
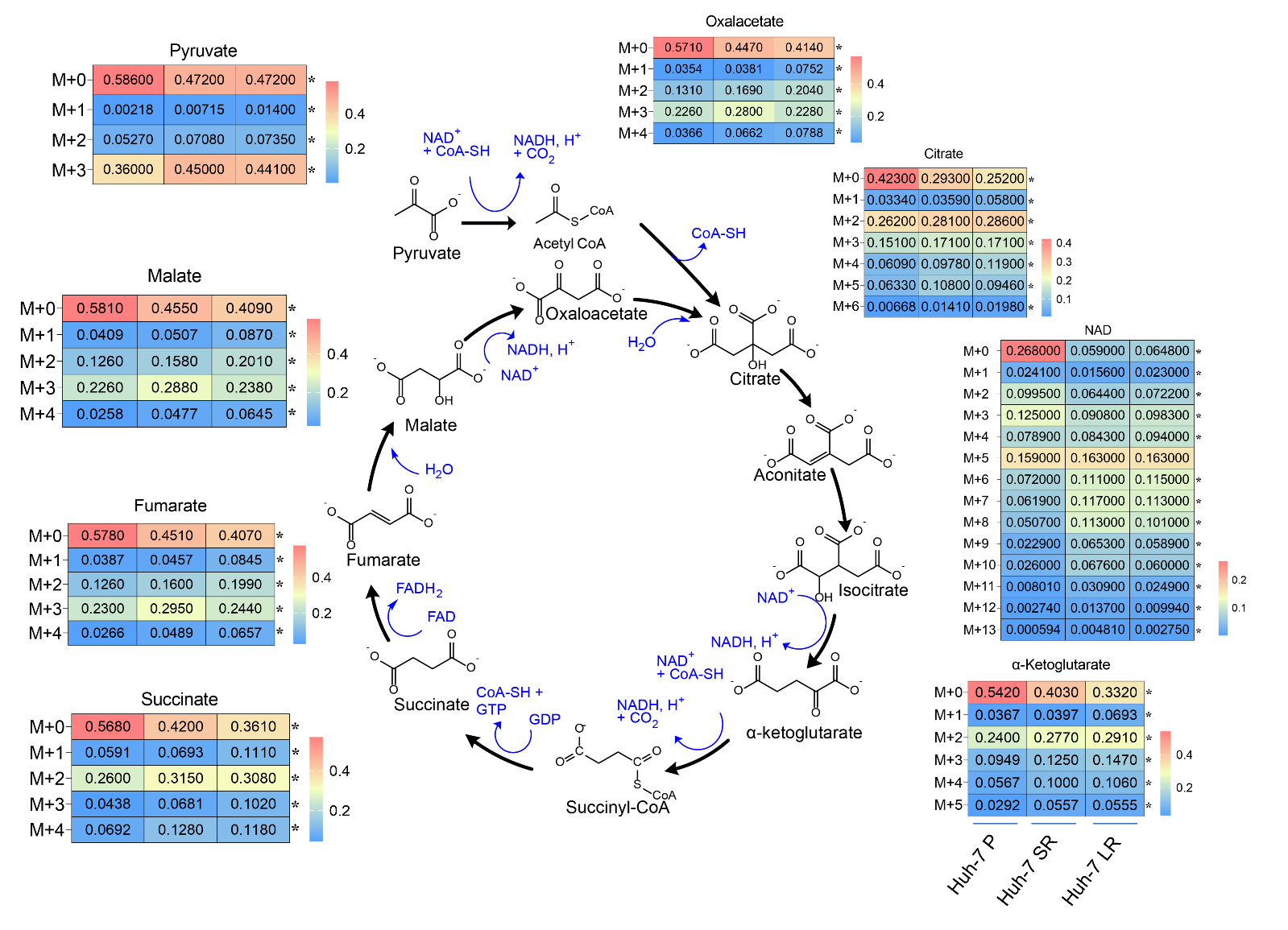
**

**Supplementary Fig. 24.** Intracellular TCA cycle metabolic flux detection. “M+number” indicates the number of additional ¹³C atoms in the metabolite molecule. “*” indicates a significant difference between the corresponding substance in Huh-7 LR/SR and Huh-7 P, with p < 0.05.

**
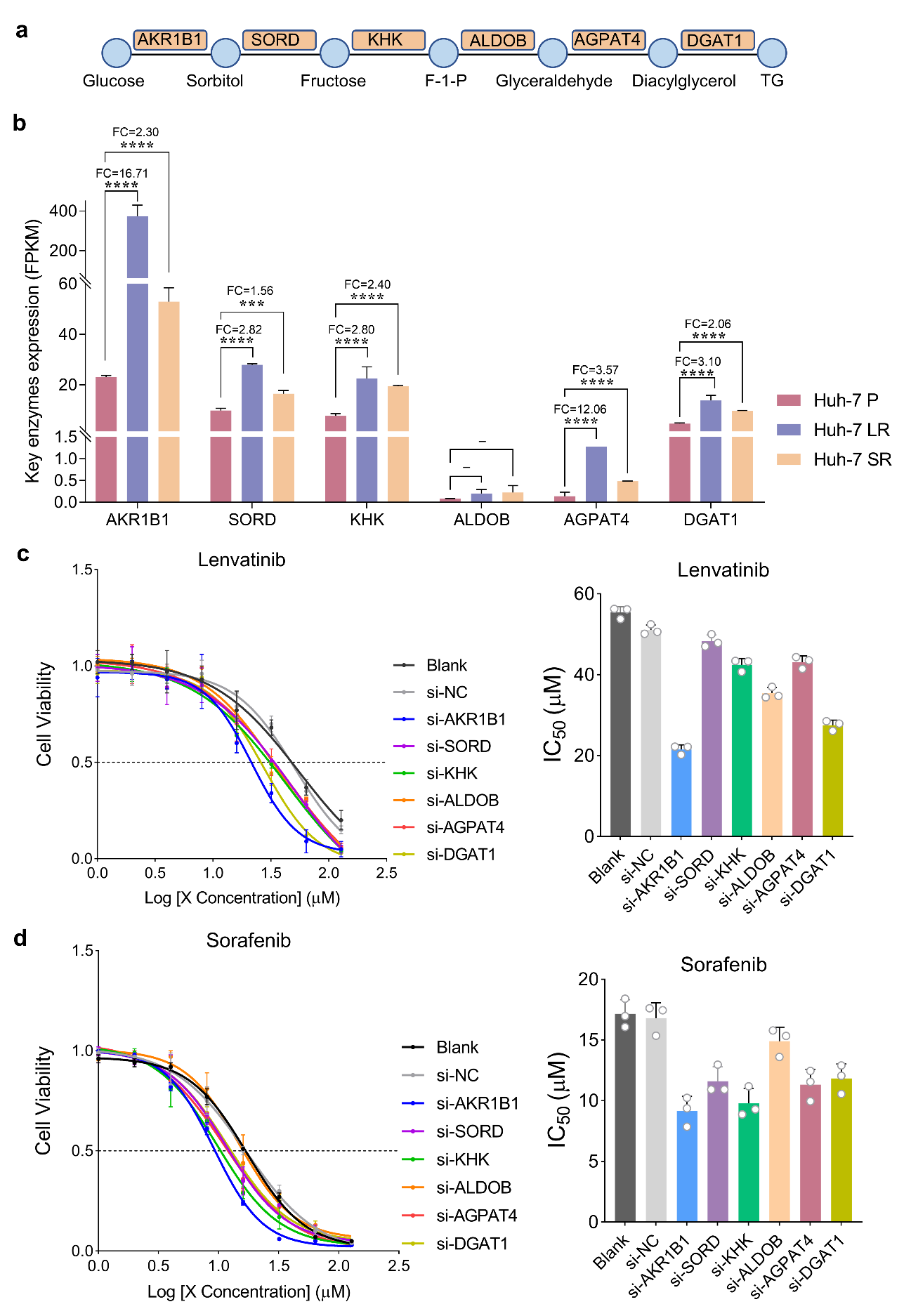
**

**Supplementary Fig. 25.** The impact of key enzymes in polyol, fructose metabolic pathways and lipogenesis pathways on the drug sensitivity of resistant cells.

(a) The key enzymes and metabolites in polyol, fructose metabolic pathways and lipogenesis pathways. (b) The expression levels of key enzymes (FPKM) and fold changes from RNA-seq data. Differential expression analysis was omitted ('-' notation) for ALDOB due to sub-threshold FPKM values (<1.0). (c) Detection of Lenvatinib sensitivity after intervention of multiple key enzymes in Huh-7 LR cell activity curves and IC_50_ values. (d) Detection of Sorafenib sensitivity after intervention of multiple key enzymes in Huh-7 SR cell activity curves and IC_50_ values.

**
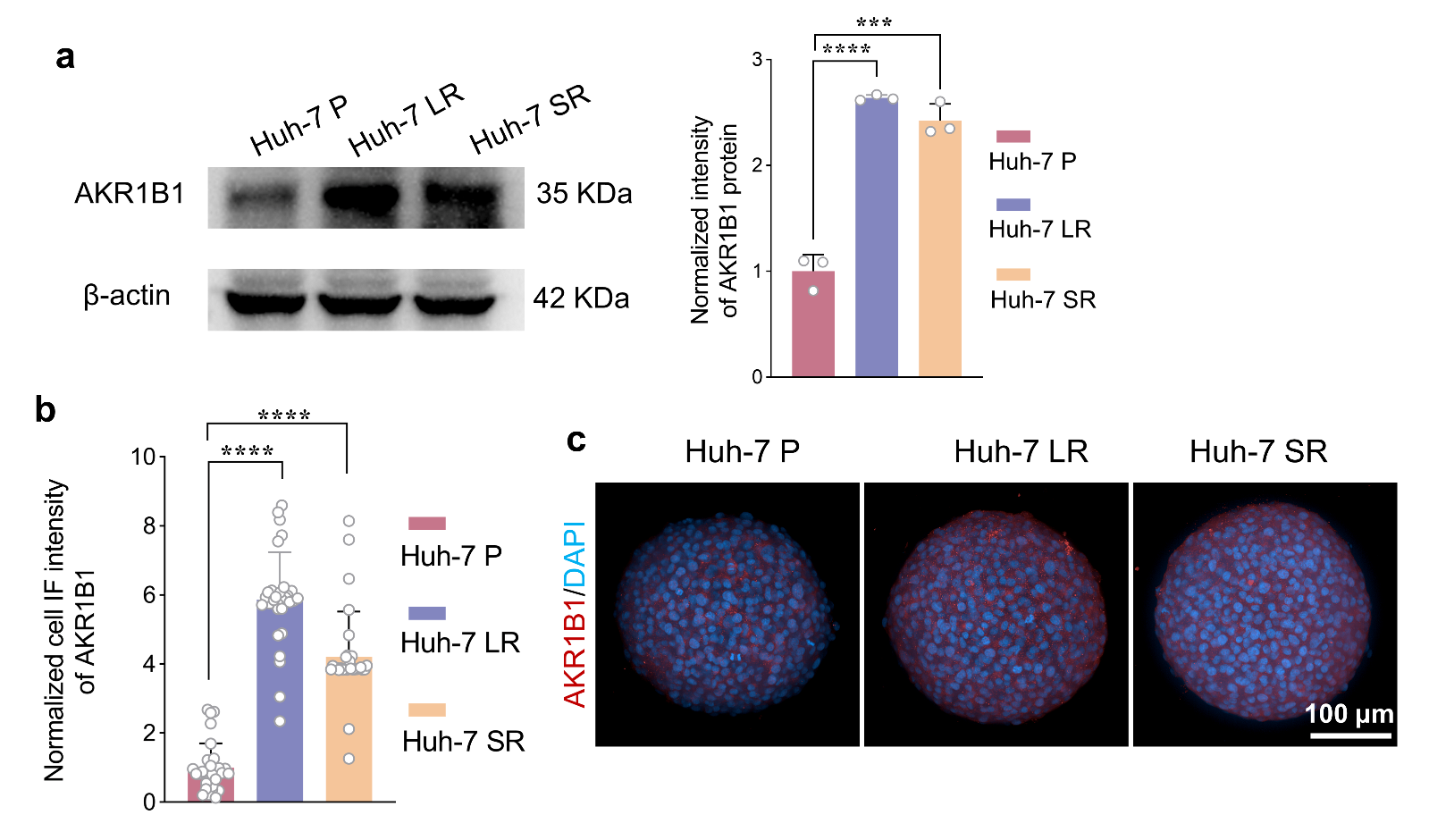
**

**Supplementary Fig. 26.** Identification of AKR1B1 in drug-resistant cells.

(a) WB confirmation of AKR1B1 expression levels in drug-resistant cells. Testing method: Unpaired Student’s t-test. (b) Statistical analysis of AKR1B1 expression levels in drug-resistant cells as determined by immunofluorescence. Testing method: Unpaired Student’s t-test. (c) 3D culture assessment of AKR1B1 expression levels in drug-resistant microtissues. AKR1B1 (red), DAPI (blue). Scale bar = 100 μm.

**
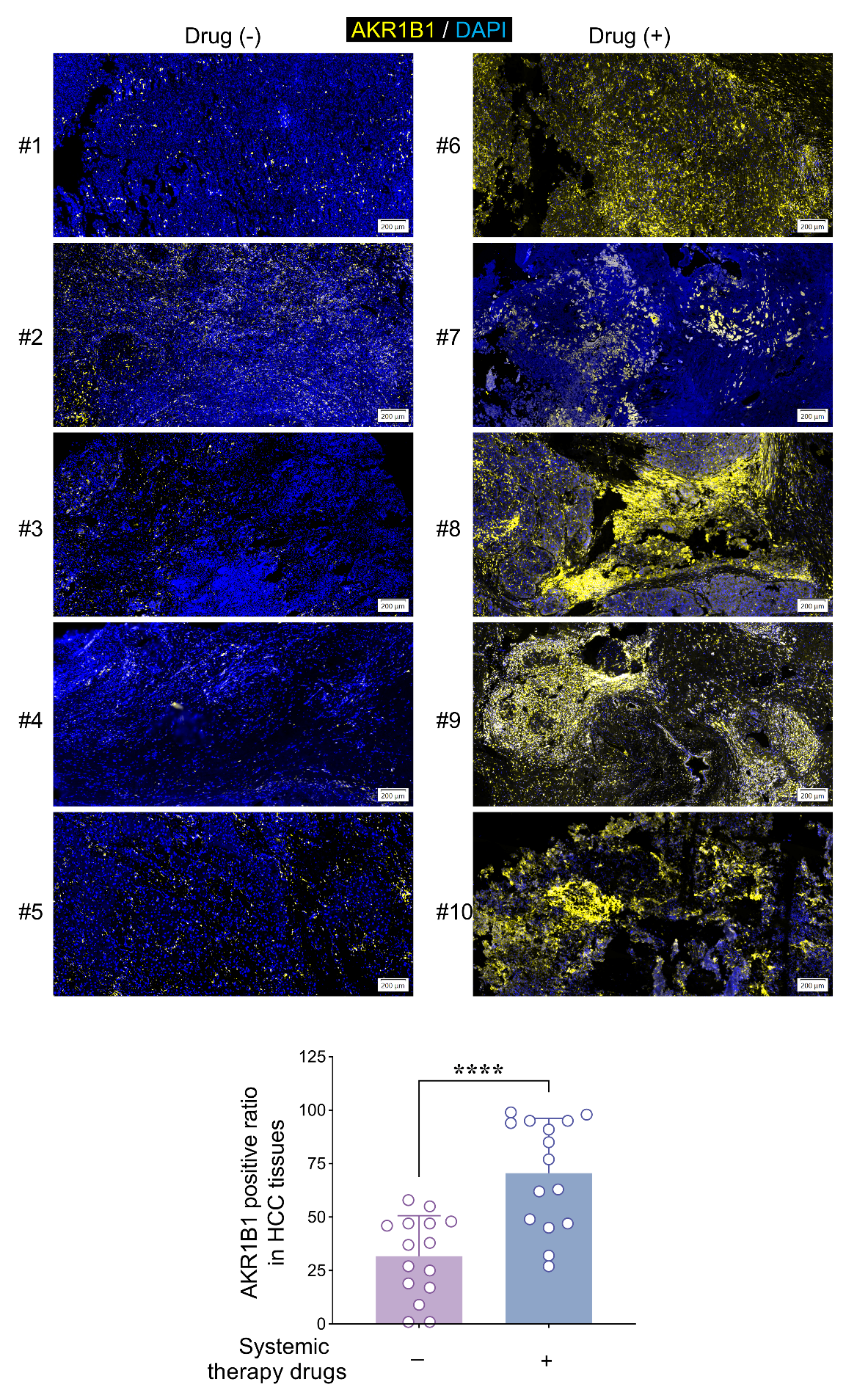
**

**Supplementary Fig. 27.** IF staining and quantification of AKR1B1 in HCC patients tumor tissues.

AKR1B1 (yellow), DAPI (blue). Scale bar = 200 μm. Testing method: Unpaired Student’s t-test.

**
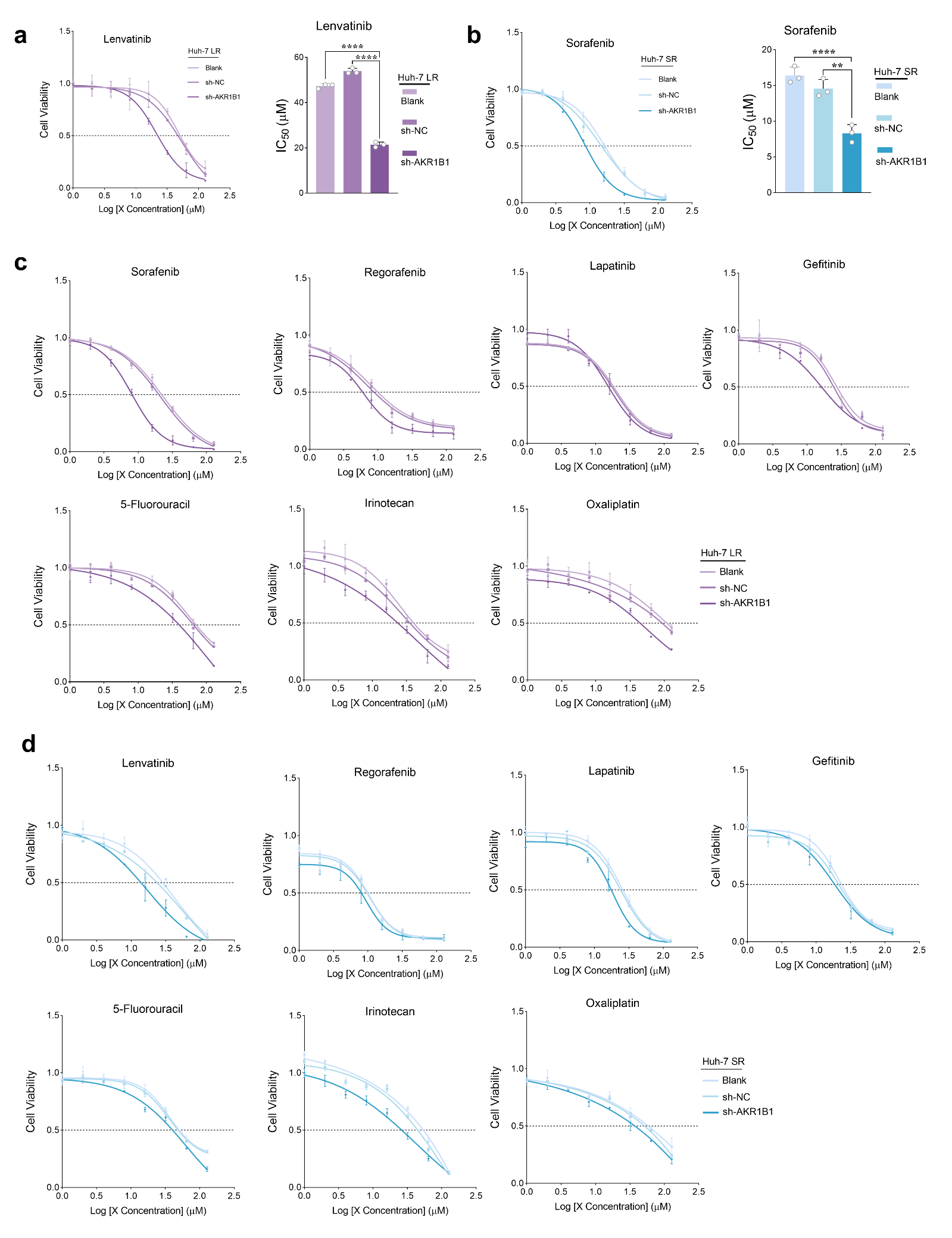
**

**Supplementary Fig. 28.** Assessment of drug sensitivity in drug-resistant cells after intervention of AKR1B1.

(a) Lenvatinib sensitivity testing after AKR1B1 intervention in Huh-7 LR cells cellular activity curve and IC_50_ value. Testing method: Unpaired Student’s t-test. (b) Sorafenib sensitivity testing after AKR1B1 intervention in Huh-7 SR cells cellular activity curve and IC_50_ values. Testing method: Unpaired Student’s t-test. (c) Cellular activity curves for multiple drugs after AKR1B1 intervention in Huh-7 LR cells. (d) Cellular activity curves for multiple drugs after AKR1B1 intervention in Huh-7 SR cells.

**
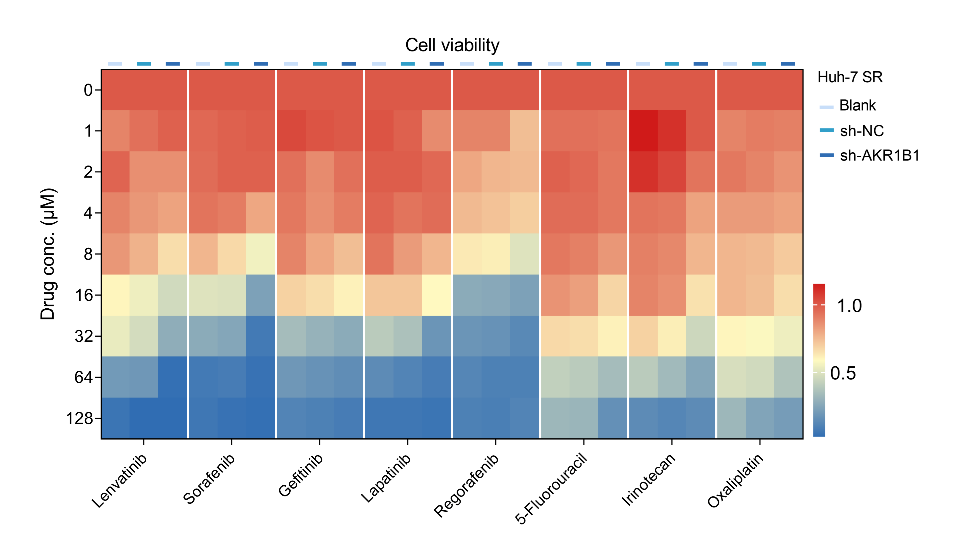
**

**Supplementary Fig. 29.** Analysis of drug sensitivity in Huh-7 SR after knockdown of AKR1B1.

**
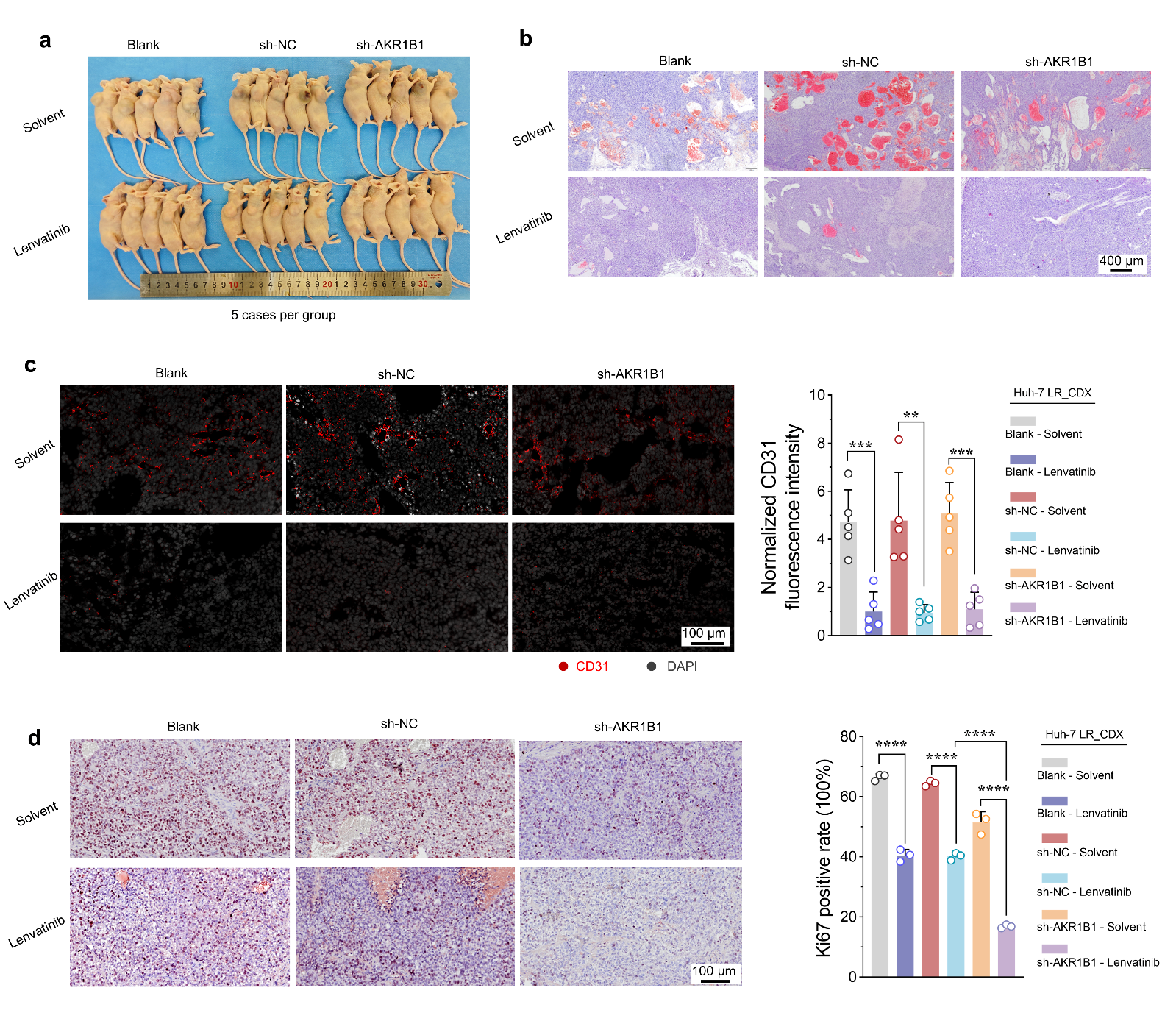
**

**Supplementary Fig. 30.** In vivo experiments following the intervention of AKR1B1 in drug-resistant cells.

(a) Drug sensitivity testing of tumor tissues from nude mice after AKR1B1 intervention in drug-resistant cells (n=5/group). (b) H&E staining of tumor tissues from nude mice after AKR1B1 intervention. Scale bar = 400 μm. (c) IF staining image and statistics of CD31 (a vascular endothelium marker) in tumor tissues from CDX models of drug-resistant and parental cells. Scale bar = 100 μm. Testing method: Unpaired Student’s t-test. (d) Ki-67 IHC staining of tumor tissues from nude mice after AKR1B1 intervention. Scale bar = 100 μm. Testing method: Unpaired Student’s t-test.

**
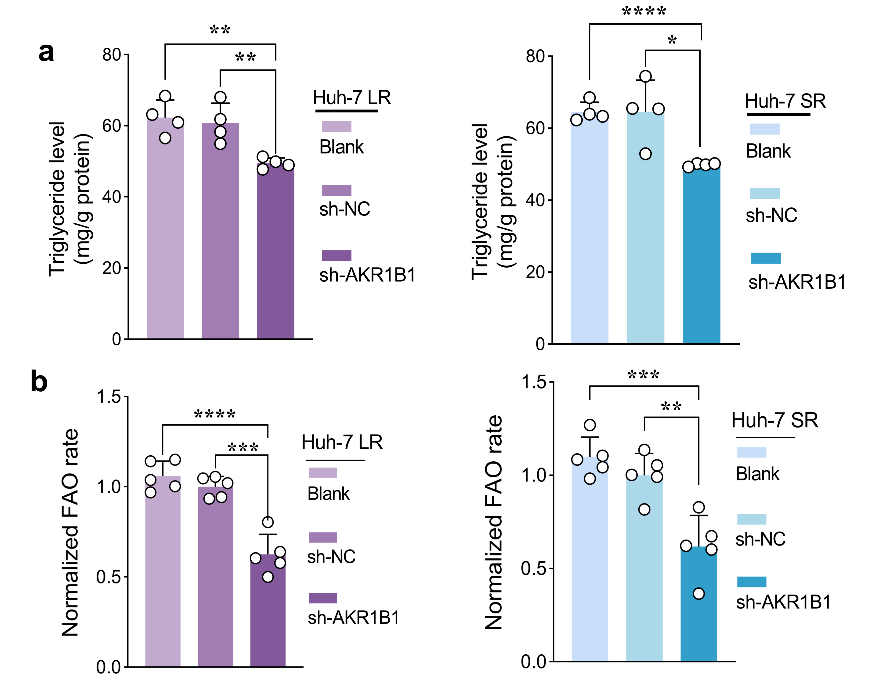
**

**Supplementary Fig. 31.** Intervention of AKR1B1 in drug-resistant cells affects metabolic reprogramming.

(a) Detection of triglyceride levels after knockdown of AKR1B1 in drug-resistant cells. Testing method: Unpaired Student’s t-test. (b) Detection of FAO levels after knockdown of AKR1B1 in drug-resistant cells. Testing method: Unpaired Student’s t-test.

**
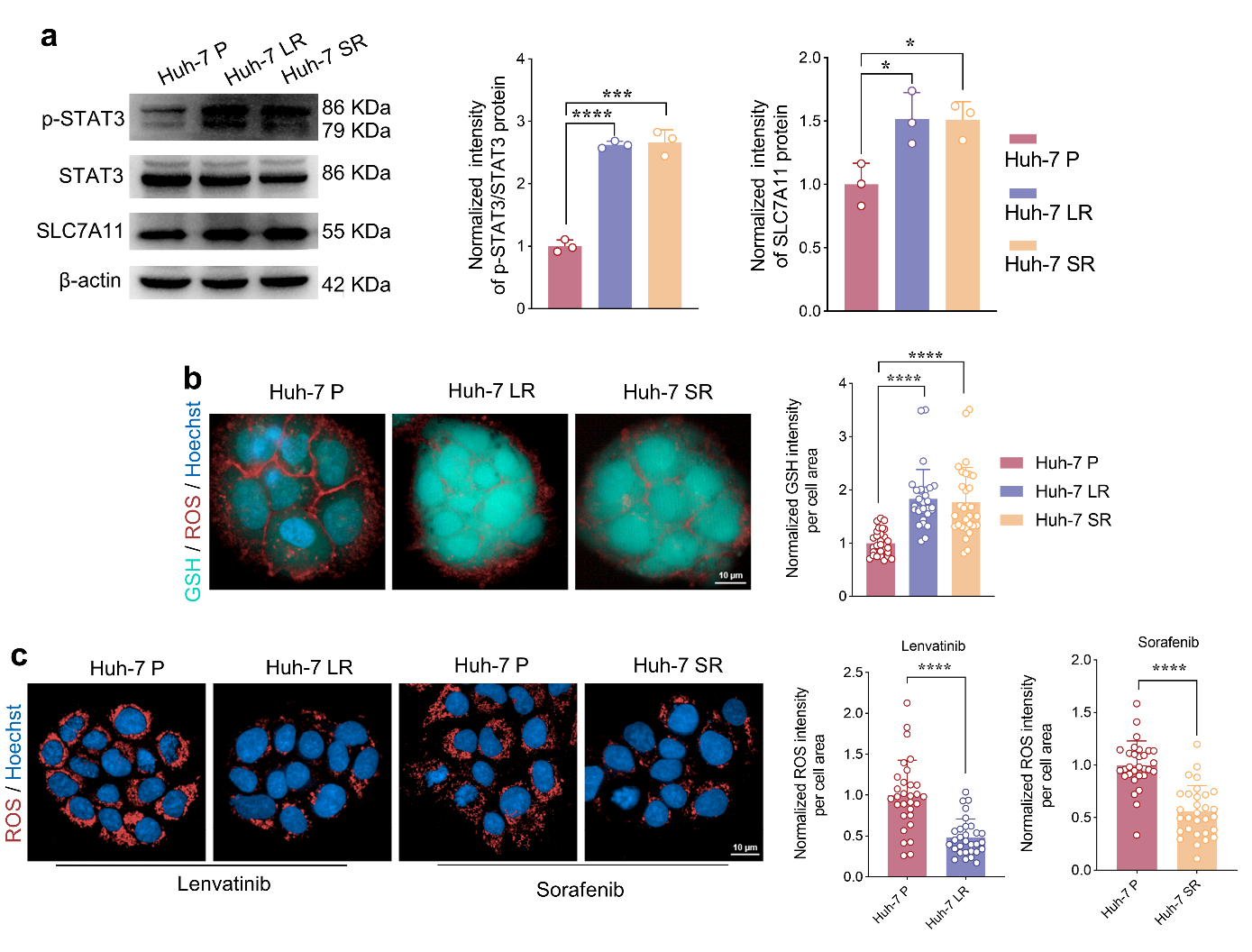
**

**Supplementary Fig. 32.** The impact of AKR1B1 on the glutathione regulating pathway.

(a) WB detection of the expression of key enzymes in the glutathione regulating pathway in drug-resistant cells. Testing method: Unpaired Student’s t-test. (b) Detection of GSH levels in drug-resistant cells. GSH (teal green), cell membrane (red), nucleus (blue). Scale bar = 10 μm. Testing method: Unpaired Student’s t-test. (c) Detection of ROS levels in drug-resistant cells. ROS (red), nucleus (blue). Scale bar = 25 μm. Testing method: Unpaired Student’s t-test.

**
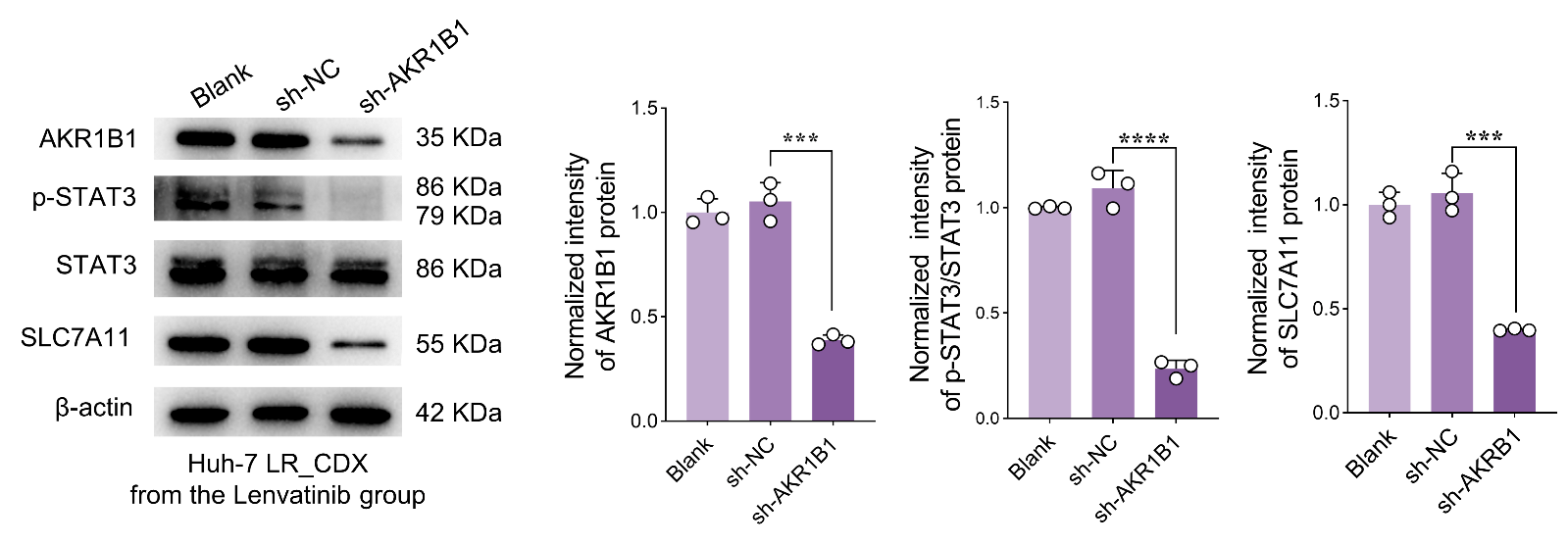
**

**Supplementary Fig. 33.** Glutathione regulating pathway enzyme profiles in Lenvatinib-treated tumors post AKR1B1 knockdown. Testing method: Unpaired Student’s t-test.


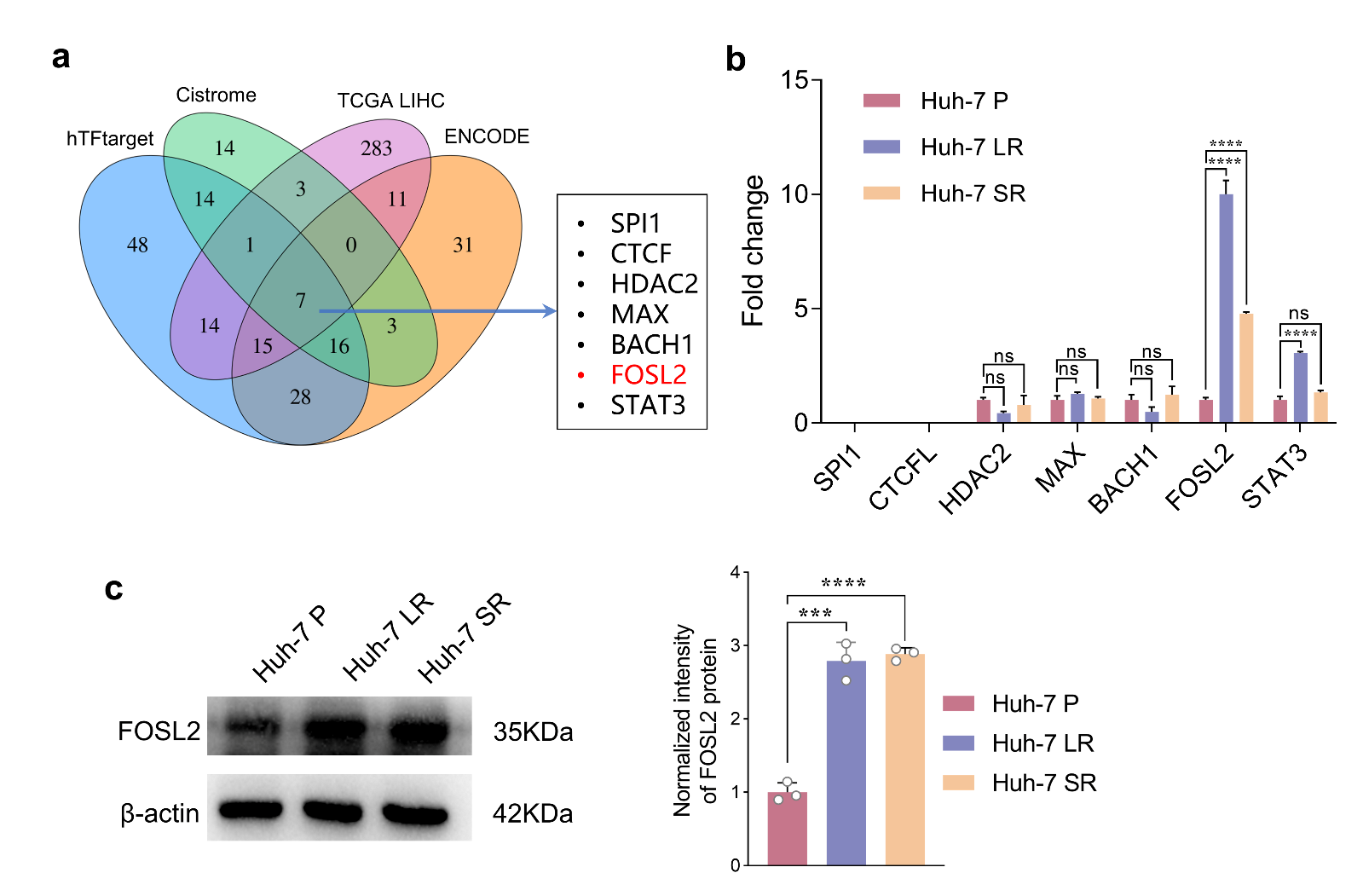


**Supplementary Fig. 34.** Predicting the transcription factors of AKR1B1 using public databases.

(a) Venn diagram of transcription factors predicted for AKR1B1 by public databases (Cistrome, TCGA_LIHC, hTFtarget, ENCODE). (b) Fold changes of the predicted transcription factors in transcriptome sequencing. Testing method: DESeq2. (c) Expression levels of FOSL2 in drug-resistant cell lines compared to parental cell lines. Testing method: Unpaired Student’s t-test.


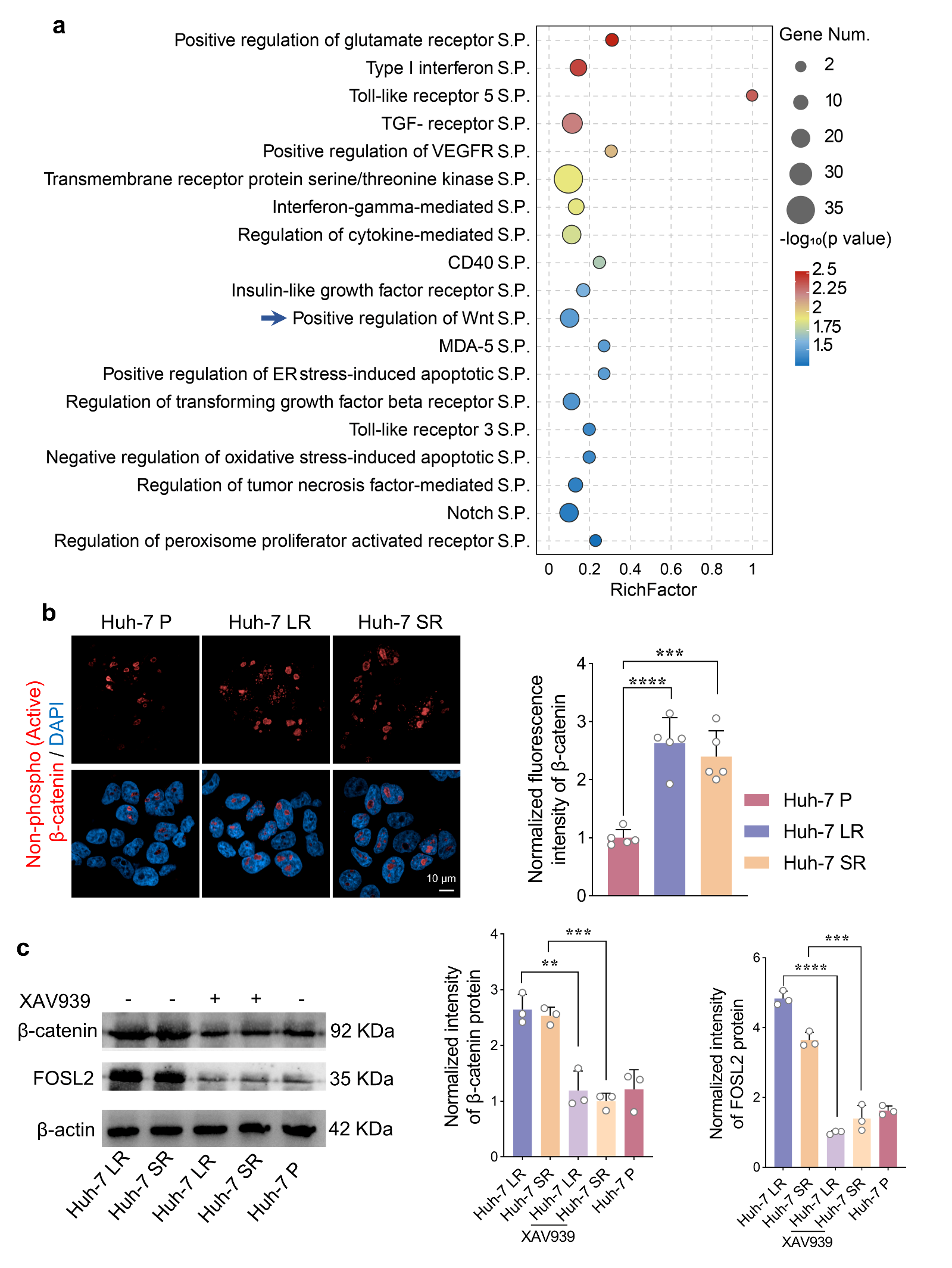


**Supplementary Fig. 35.** Drug-resistant cells upregulate FOSL2 through the Wnt signaling pathway.

(a) GO enrichment analysis of signaling pathways for genes upregulated in drug-resistant cells compared to parental cells. S.P.: signaling pathway. (b) IF staining and fluorescence intensity analysis of non-phospho (active) β-catenin. Non-phospho (active) β-catenin (red), DAPI (blue, nucleus). Scale bar = 10 μm. Testing method: Unpaired Student’s t-test. (c) WB analysis of the effects of β-catenin inhibitor (XAV939, 10 µM, 24h) on the expression of β-catenin and FOLS2. Testing method: Unpaired Student’s t-test.


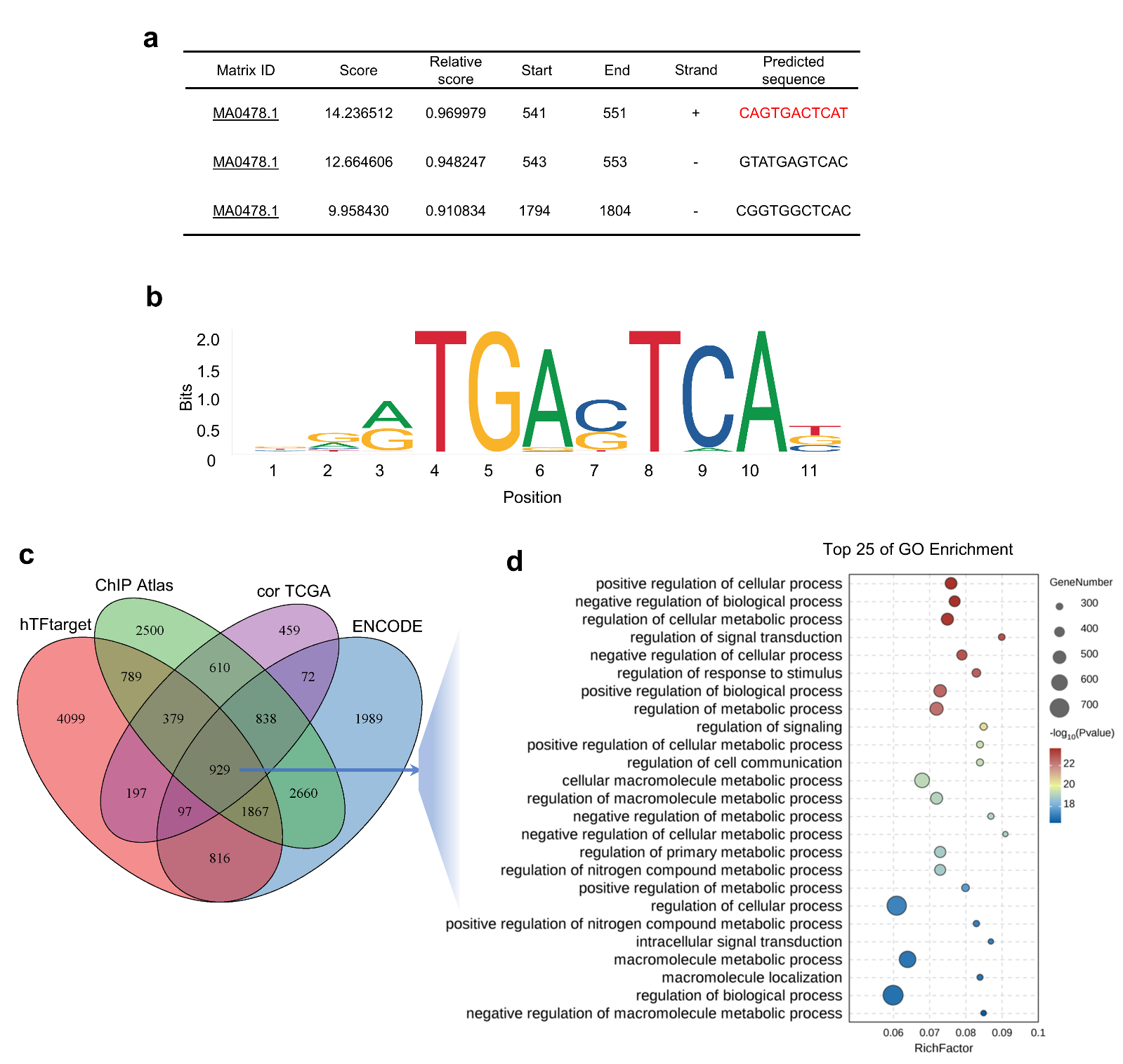


**Supplementary Fig. 36.** Prediction of FOSL2 binding sites with AKR1B1 and functional analysis of downstream target genes.

(a) JASPAR matrix models predict the binding sites of AKR1B1 with the transcription factor FOSL2. (b) Sequence logo for #MA0478.1 (CAGTGACTCAT). (c) Venn diagram of downstream target genes of FOSL2 predicted by public databases (hTFtarget, ChIP Atlas, cor TCGA, ENCODE). (d) GO enrichment analysis of downstream target genes of FOSL2.

**
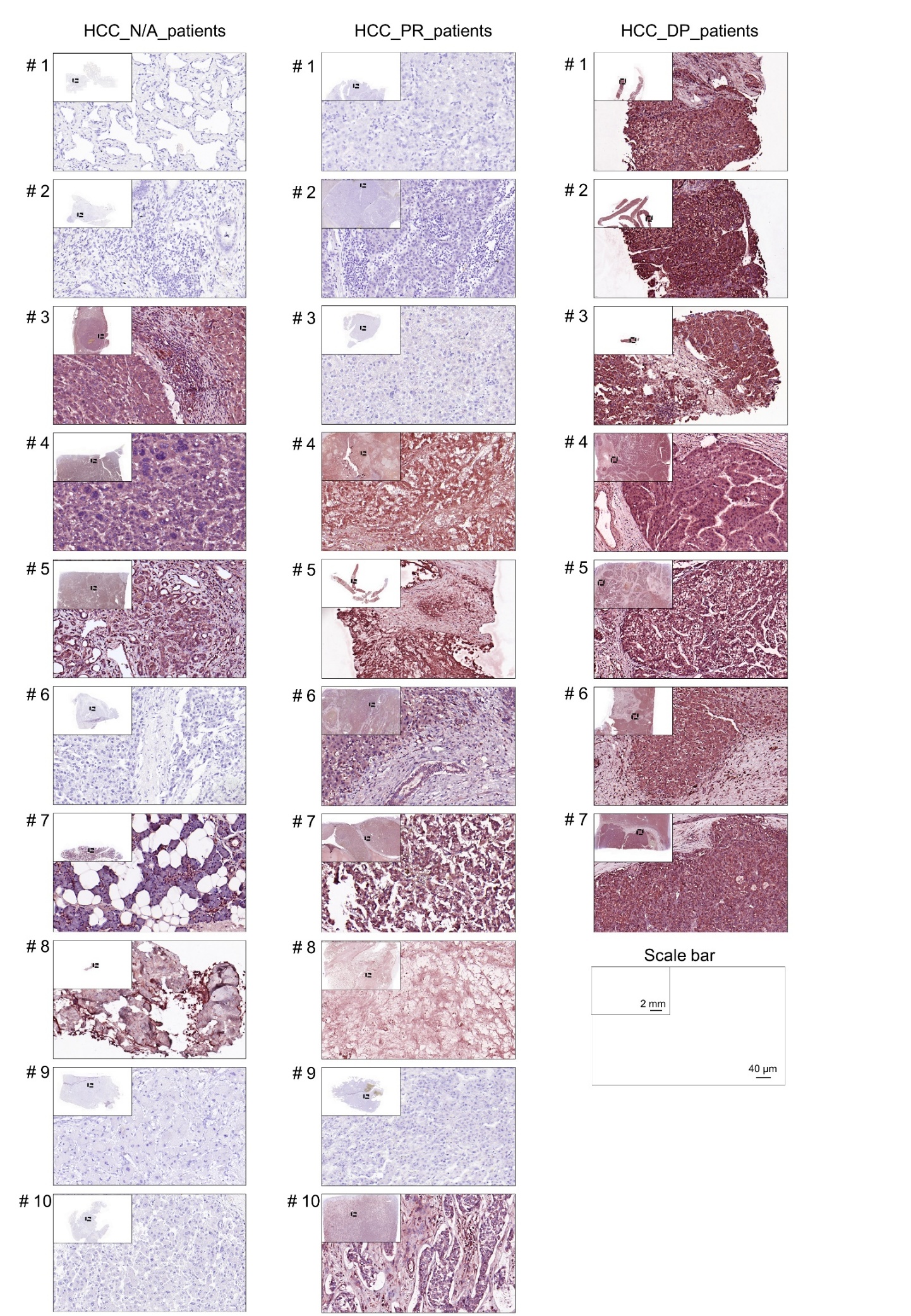
**

**Supplementary Fig. 37.** AKR1B1 IHC staining of tumor tissues from HCC patients with no drug use (N/A), partial response (PR), and disease progression (DP). Scale bar = 2 mm and 40 μm.


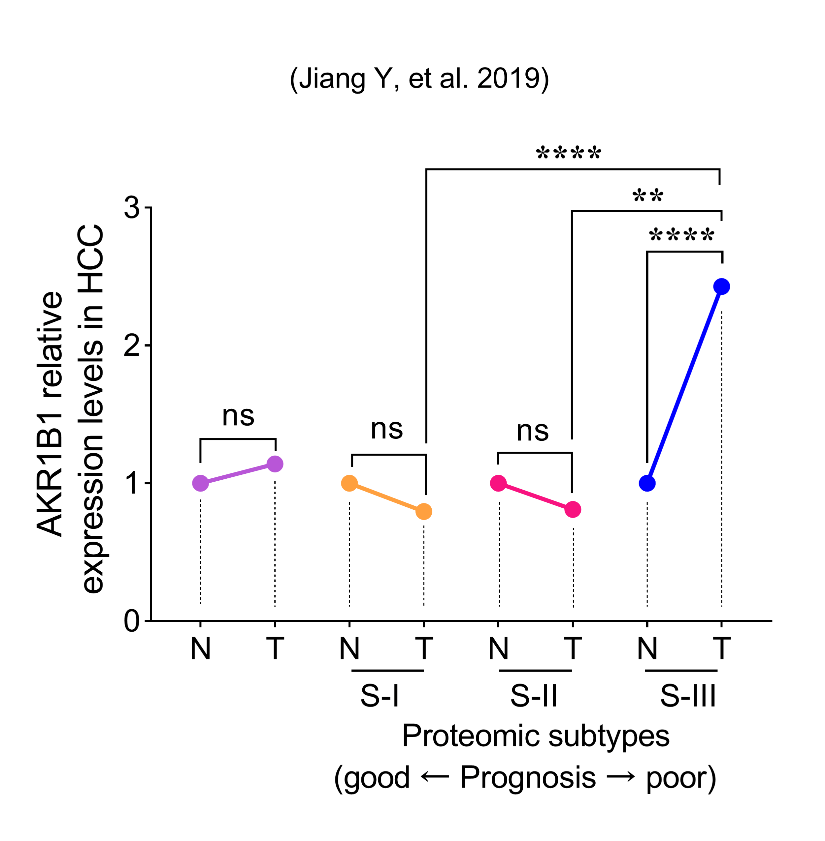


**Supplementary Fig. 38.** Protein expression levels of AKR1B1 in tumor tissues versus adjacent normal tissues in HCC patients categorized by proteomic subclasses^10^. Testing method: Unpaired Student’s t-test.


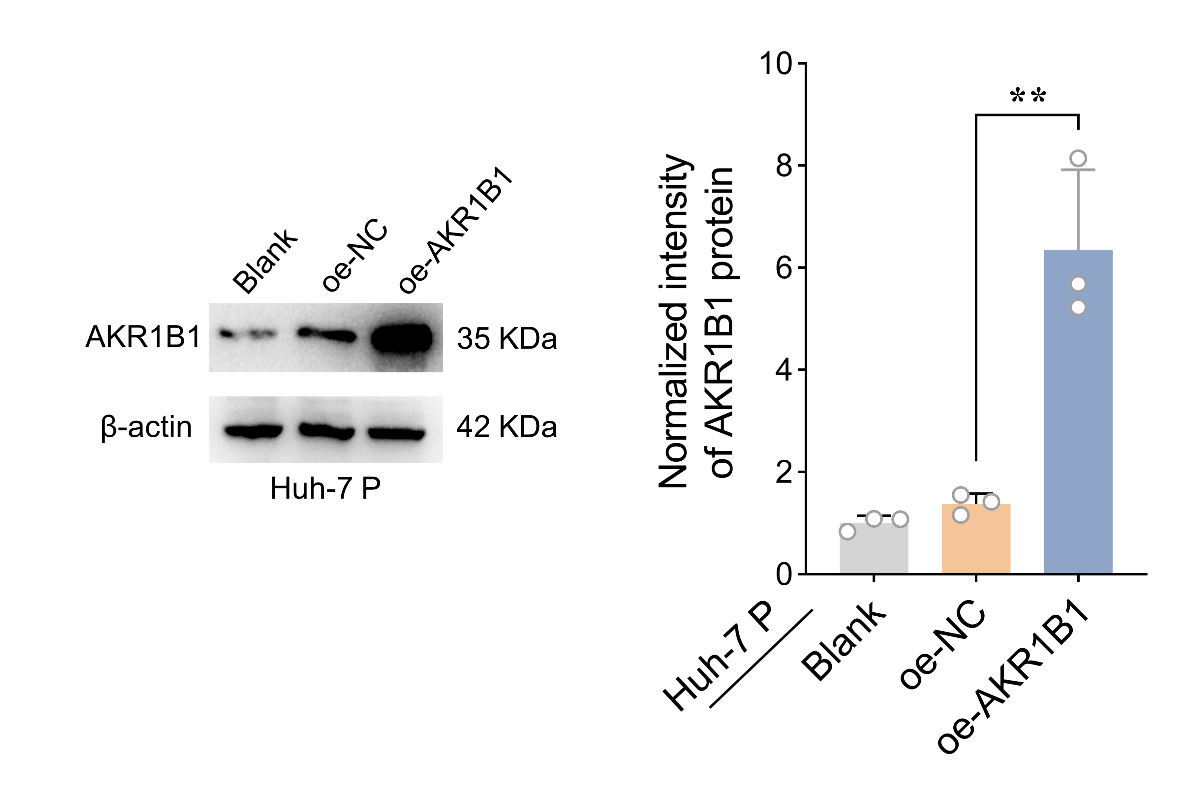


**Supplementary Fig. 39.** WB detection of AKR1B1 expression in parental cells after overexpression of AKR1B1. Testing method: Unpaired Student’s t-test.


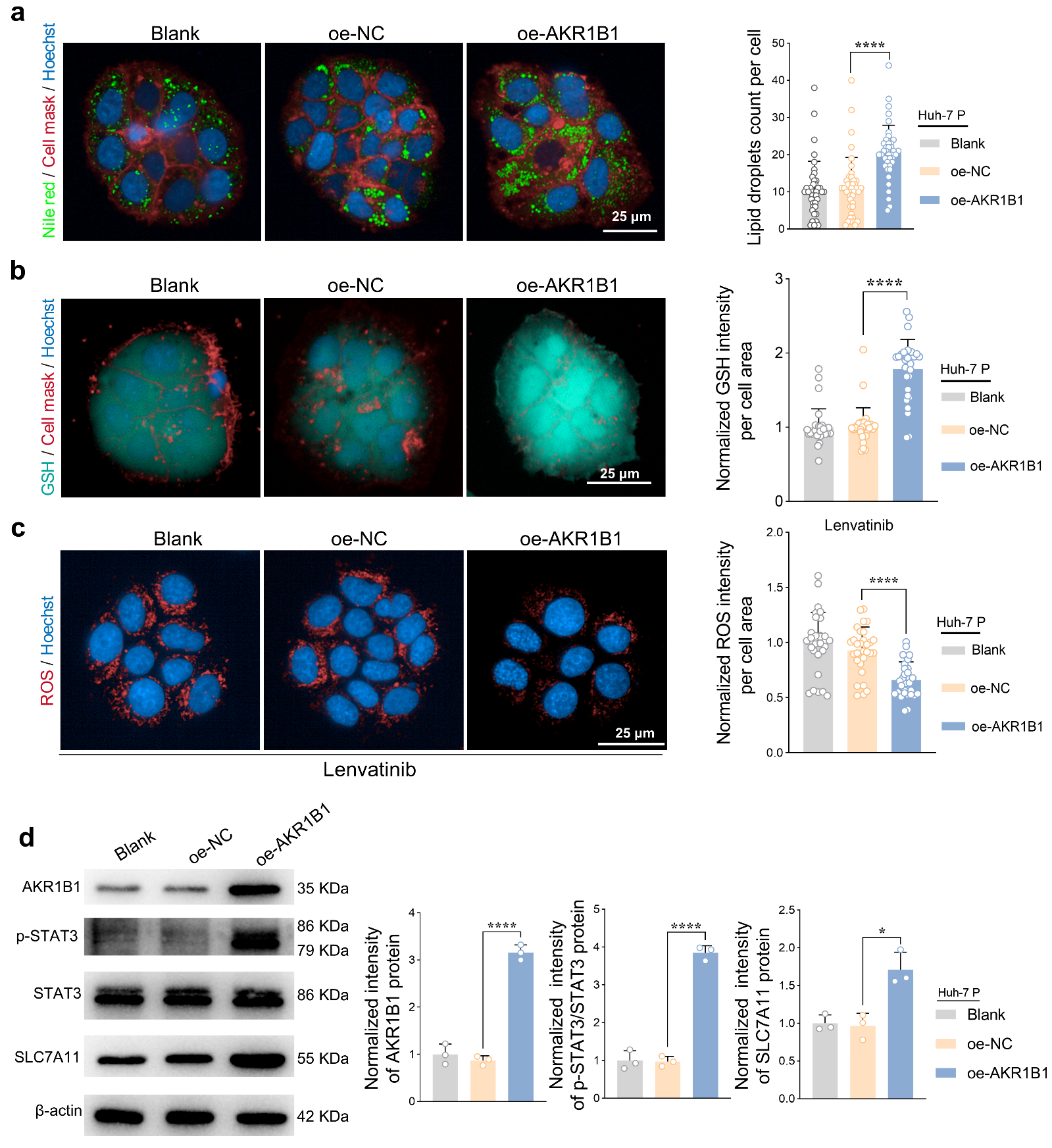


**Supplementary Fig. 40.** Metabolic detection of parental cells overexpressing AKR1B1.

(a) Detection of intracellular lipid droplet content after overexpression of AKR1B1 in parental cells. Lipid droplets (green), cell membrane (red), nucleus (blue). Scale bar = 25 μm. Testing method: Unpaired Student’s t-test. (b) Detection of GSH levels after overexpression of AKR1B1 in parental cells. GSH (teal green), cell membrane (red), nucleus (blue). Scale bar = 25 μm. Testing method: Unpaired Student’s t-test. (c) Detection of ROS levels after overexpression of AKR1B1 in parental cells. ROS (red), nucleus (blue). Scale bar = 25 μm. Testing method: Unpaired Student’s t-test. (d)WB detection of the expression of key enzymes in the GSH metabolism pathway following overexpression of AKR1B1 in parental cells. Testing method: Unpaired Student’s t-test.


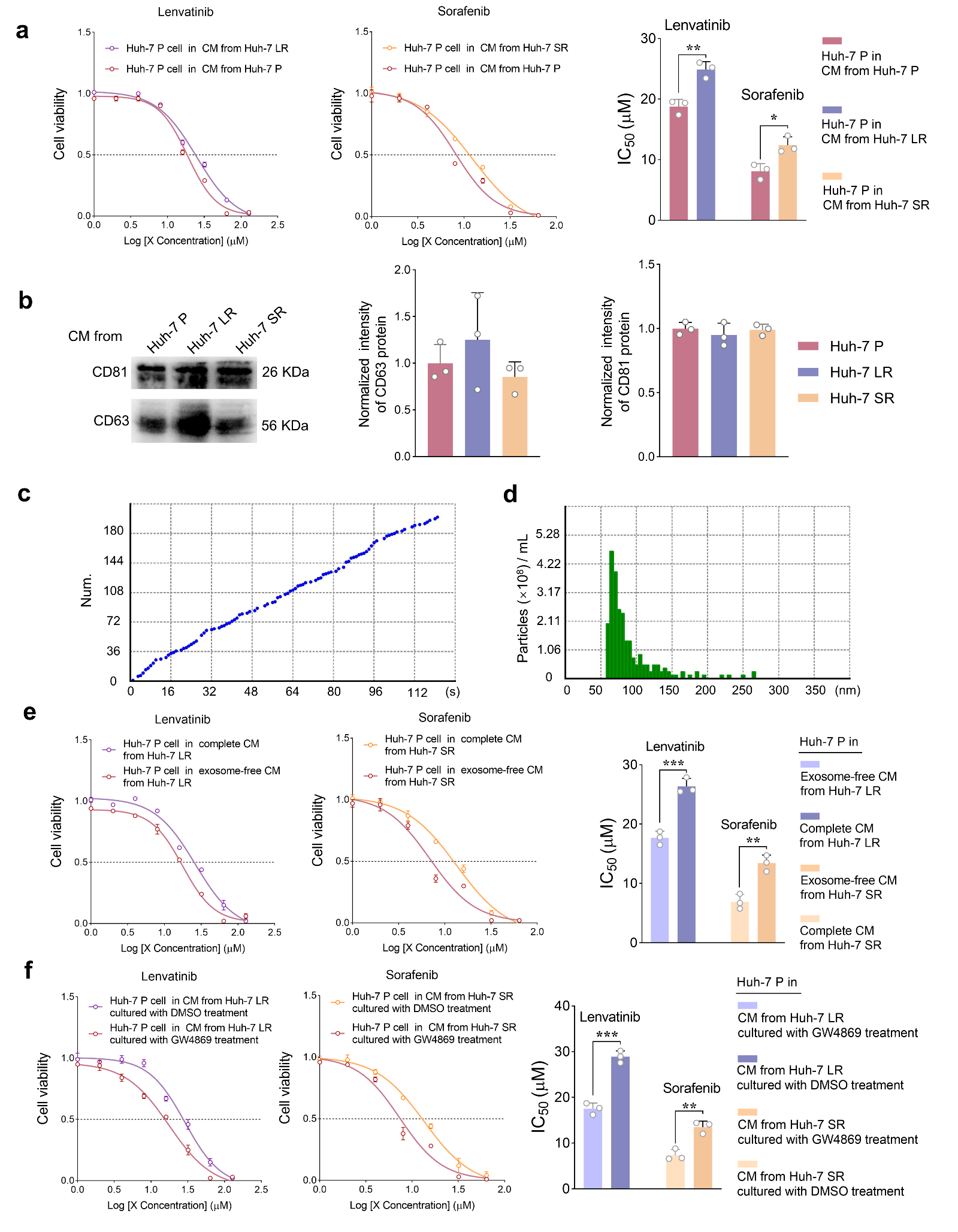


**Supplementary Fig. 41.** Mediation of drug resistance transfer by conditional medium.

(a) Cytotoxicity assay to detect the cellular activity curve and drug IC_50_ values for drug resistance transfer mediated by conditional medium. Testing method: Unpaired Student’s t-test. (b) Identification of exosomal protein CD81 and CD63 expression levels. Testing method: Unpaired Student’s t-test. (c) The trend of detected exosome particle count over detection duration. (d) The size distribution frequency of the tested exosomes. (e) The effect of exosome-free conditioned medium on drug resistance transfer. Testing method: Unpaired Student’s t-test. (f) The impact of conditioned medium inhibiting exosome secretion on drug - resistance transfer. (GW4869, 10 μM, 24 h). Testing method: Unpaired Student’s t-test.


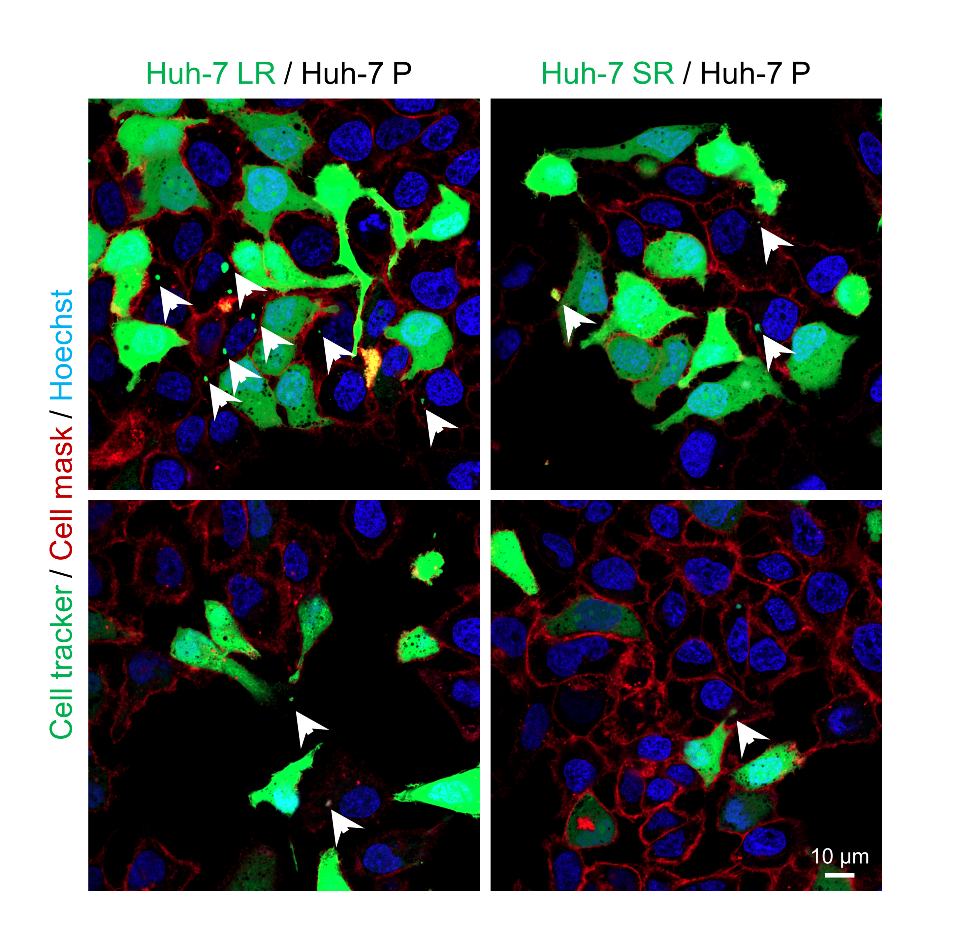


**Supplementary Fig. 42.** Cell tracing probe detection of material transfer from drug-resistant cells to parental cells. Cell tracing (green) — drug-resistant cells, cell membrane (red), nucleus (blue). Scale bar = 10 μm.


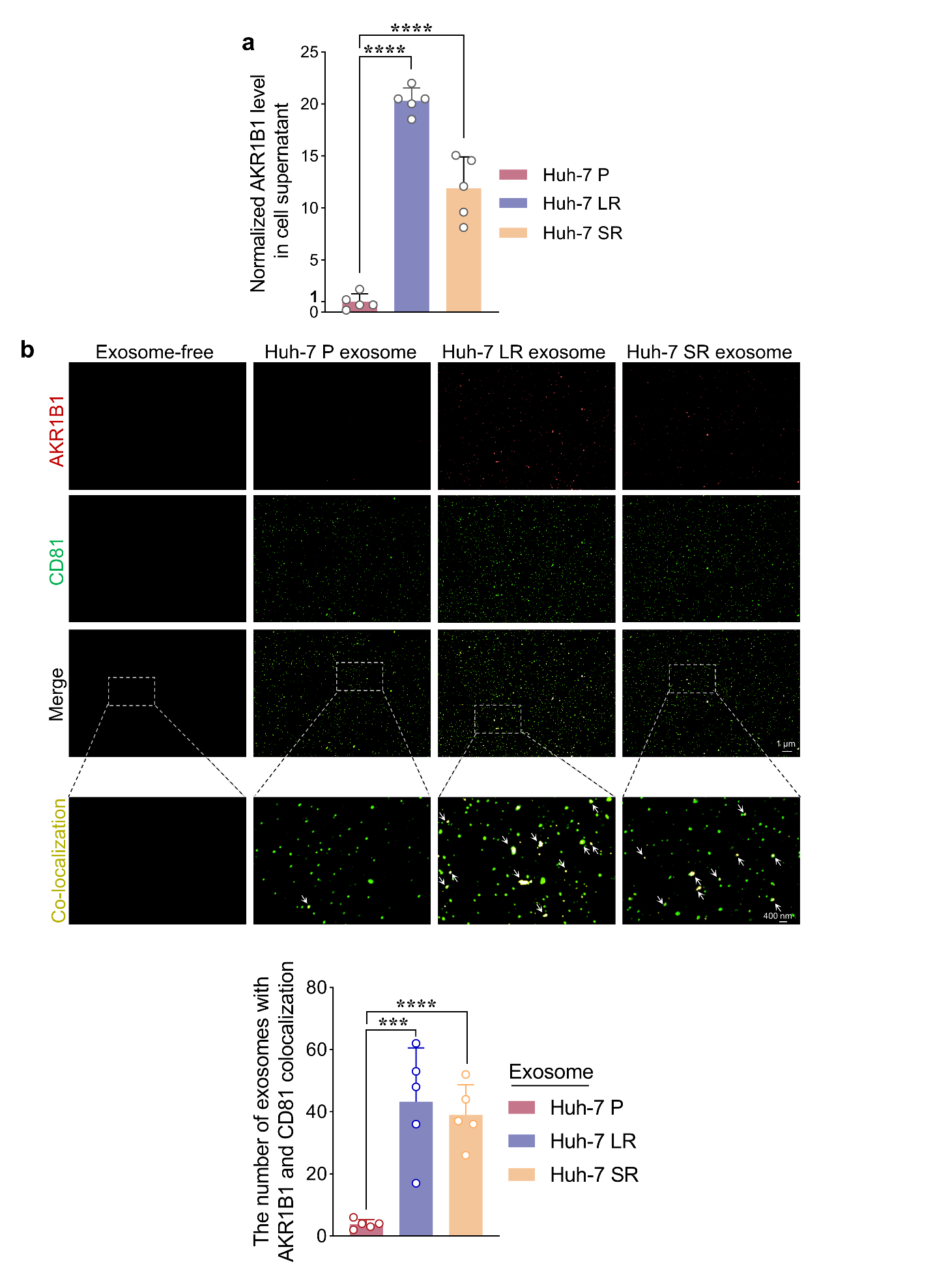


**Supplementary Fig. 43.** Exosome and AKR1B1 colocalization detection.

(a) Detection of AKR1B1 in the supernatant of drug-resistant cell culture. Testing method: Unpaired Student’s t-test. (b) IF detection of the colocalization of CD81 and AKR1B1 in purified exosomes. AKR1B1 (red), CD81 (Green). Scale bar = 1 μm / 400 nm. Testing method: Unpaired Student’s t-test.


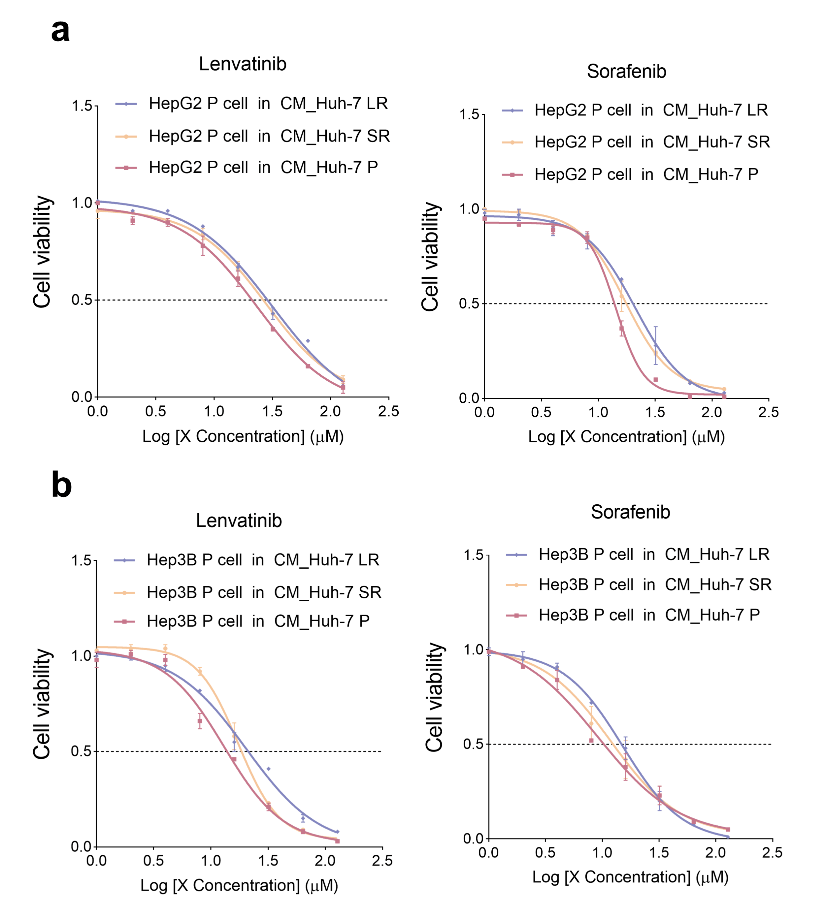


**Supplementary Fig. 44.** Conditioned medium from drug-resistant cells enhances drug resistance in multiple types of HCC cells.

(a) Cytotoxicity assay to detect the cellular activity curve for drug resistance transfer mediated by conditional medium in HepG2 cell line. (b) Cytotoxicity assay to detect the cellular activity curve for drug resistance transfer mediated by conditional medium in Hep3B cell line.


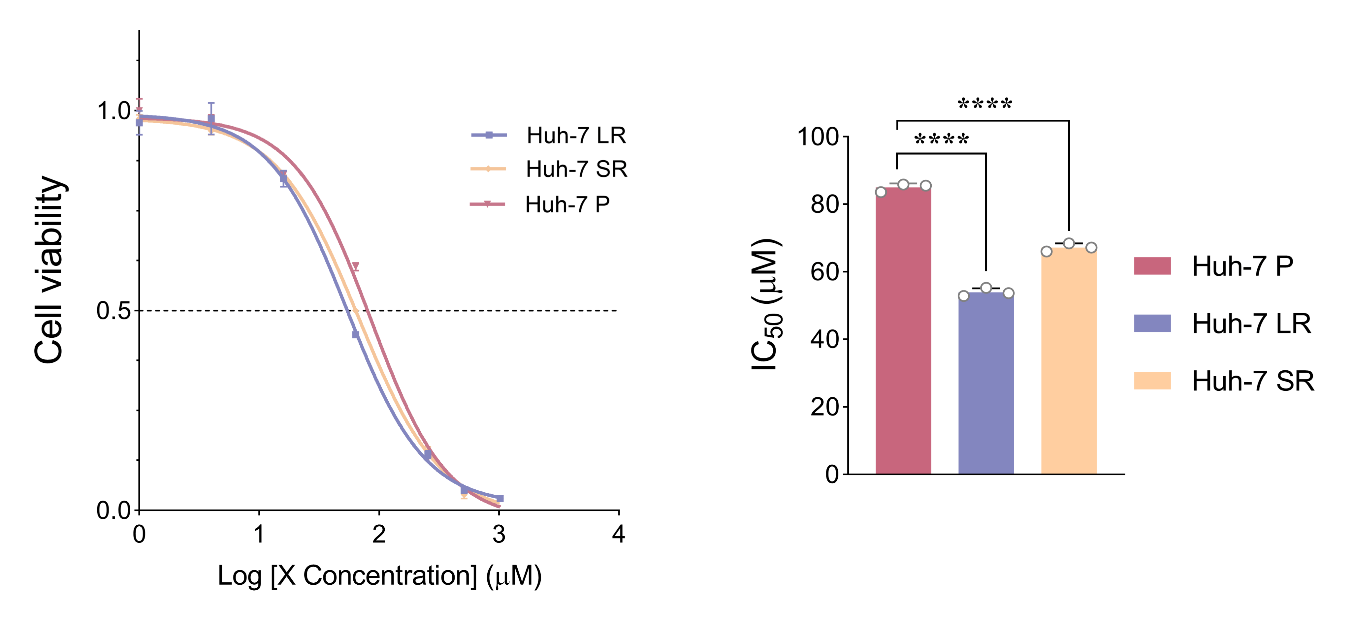


**Supplementary Fig. 45.** Cellular activity curve and IC_50_ values of drug-resistant cells and parental cells treated with Epalrestat. Testing method: Unpaired Student’s t-test.

**
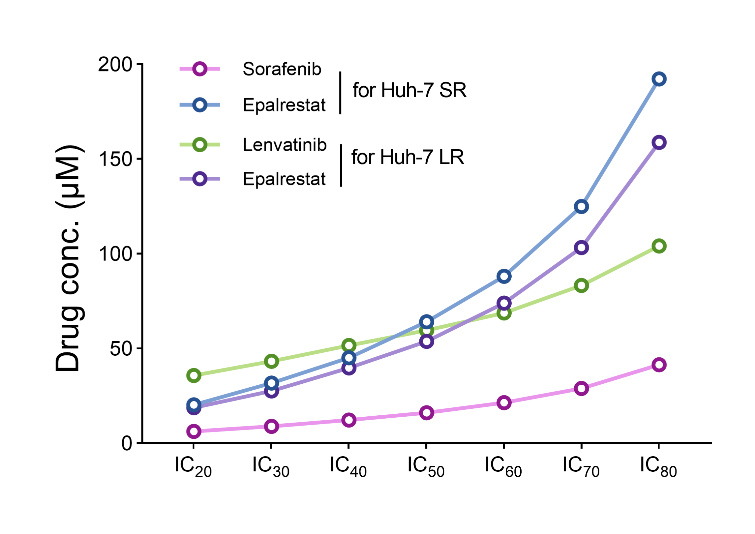
**

**Supplementary Fig. 46.** Inhibitory concentration detection of Epalrestat and targeted therapies as single agents in drug-resistant cell lines.


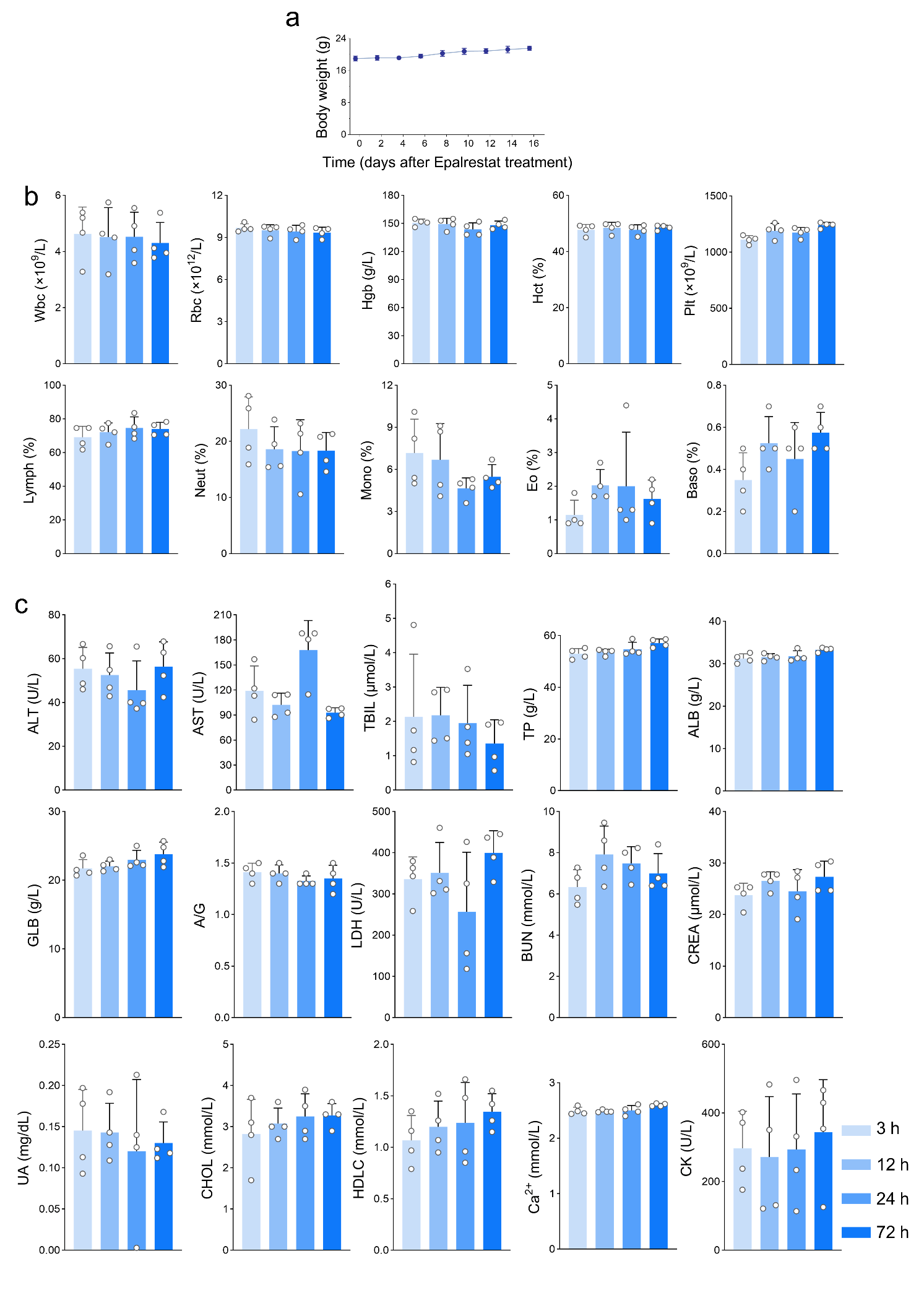


**Supplementary Fig. 47.** Epalrestat toxicity testing in vivo.

(a) Epalrestat's impact on animal model weight changes (50 mg/kg/d). (b) Epalrestat's impact on hematological indices in animal models (50 mg/kg). (c) Epalrestat's impact on blood biochemical indices in animal models (50 mg/kg).

**
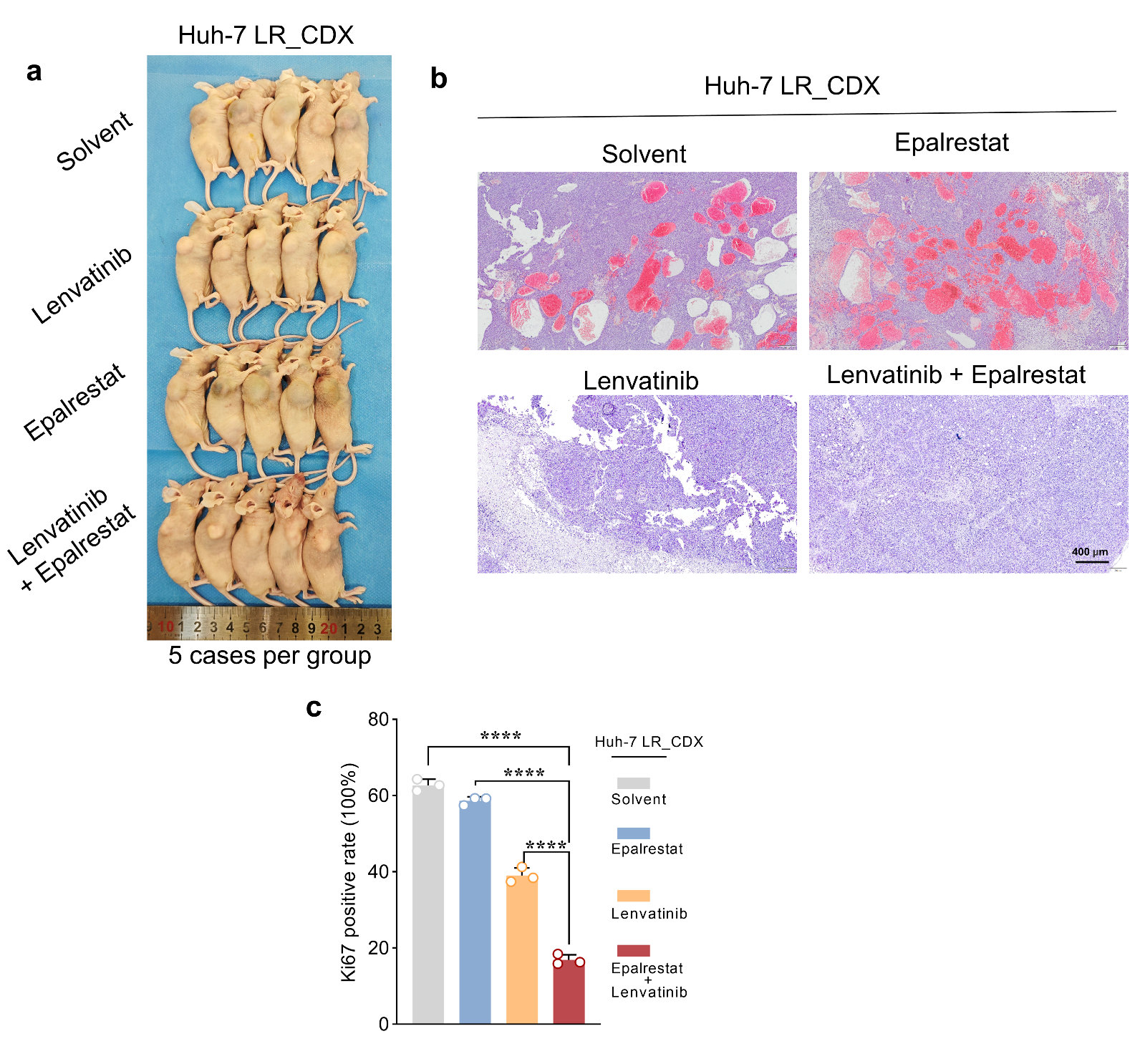
**

**Supplementary Fig. 48.** In vivo experiments to test the improvement of drug resistance by combination therapy.

(a) In vivo experiment to detect the effect of the combined application of Epalrestat and targeted therapy (n=5/group). (b) H&E staining of tumor tissues from nude mice treated with the combination therapy. Scale bar = 400 μm. (c) Ki-67 IHC staining quantification of tumor tissues from nude mice treated with the combination therapy. Testing method: Unpaired Student’s t-test.

**
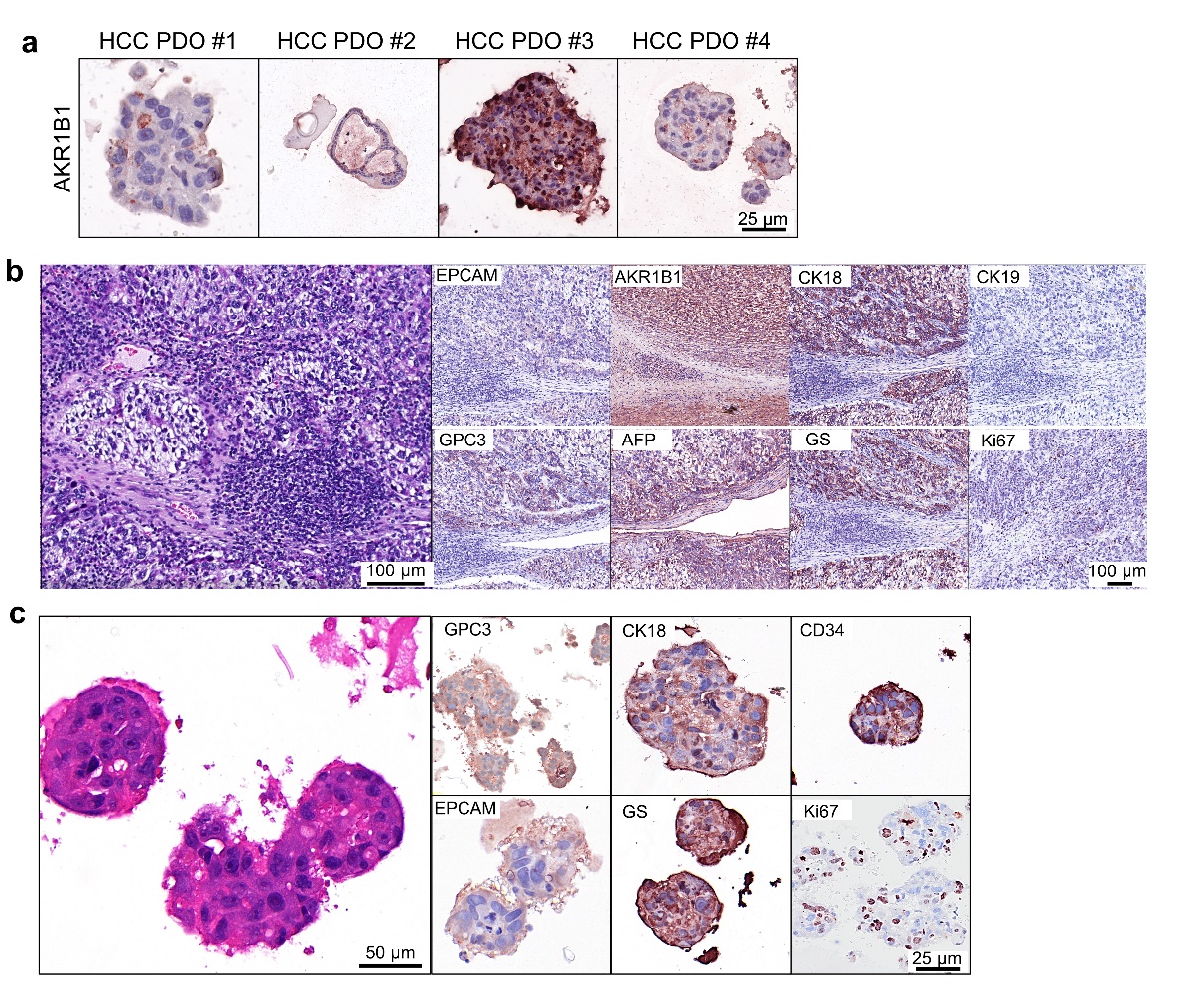
**

**Supplementary Fig. 49.** Identification of HCC PDOs.

(a) IHC detection of AKR1B1 expression in 4 cases of PDOs. Scale bar = 25 μm. (b) Identification of HCC characteristics and tumor composition in case with high expression of AKR1B1 by H&E and IHC staining. Scale bar = 100 μm. (c) Identification of HCC characteristics and tumor composition in PDO with high expression of AKR1B1 by H&E and IHC staining. Scale bar = 50 μm (H&E); 25 μm (IHC).

**
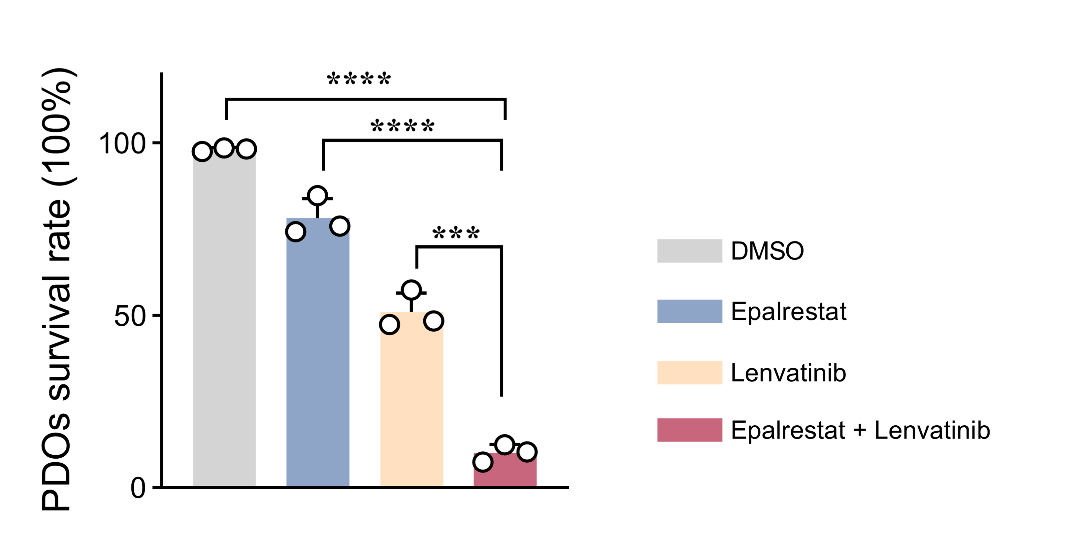
**

**Supplementary Fig. 50.** Live/dead probe staining for the statistical analysis of the survival rate of HCC PDOs after 48 h of drug treatment. Testing method: Unpaired Student’s t-test.

## Supplementary Table 1. Systemic therapy-related clinical and pathological information of HCC patients.

| Group | No. | Gender | Age (year) | T stage | HBsAg | Cirrhosis | Differentiation Grade | MVI | Child-Pugh Grading | Systemic Therapy Drugs |
| --- | --- | --- | --- | --- | --- | --- | --- | --- | --- | --- |
| N/A | N-01 | Male | 46 | T1a | Yes | Yes | Moderate | M0 | B | - |
|  | N-02 | Male | 55 | T2 | Yes | Yes | Moderate | M0 | B | - |
|  | N-03 | Male | 47 | T4 | Yes | Yes | Moderate | M2 | C | - |
|  | N-04 | Female | 13 | T2 | No | Yes | Moderate | M2 | B | - |
|  | N-05 | Male | 40 | T4 | Yes | Yes | Moderate | M2 | C | - |
|  | N-06 | Male | 50 | T1b | Yes | Yes | Moderate | M0 | B | - |
|  | N-07 | Female | 59 | T1b | No | Yes | Moderate | M0 | B | - |
|  | N-08 | Male | 77 | T1b | Yes | Yes | Moderate | M0 | B | - |
|  | N-09 | Male | 50 | T2 | Yes | Yes | Moderate | M0 | C | - |
|  | N-10 | Male | 58 | T1b | Yes | Yes | Moderate | M0 | B | - |
| PR | R-01 | Female | 76 | T2 | Yes | Yes | Moderate-High | M1 | B | TACE (Oxaliplatin + Fluorouracil)  Atezolizumab  Bevacizumab |
|  | R-02 | Male | 62 | T2 | Yes | Yes | Moderate | M1 | B | TACE (Oxaliplatin + Fluorouracil)  Lenvatinib  Camrelizumab |
|  | R-03 | Male | 54 | T1b | Yes | Yes | Moderate | M0 | B | TACE (Oxaliplatin + Fluorouracil)  Lenvatinib  Pembrolizumab |
|  | R-04 | Female | 61 | T2 | Yes | Yes | Moderate | M2 | C | Atezolizumab  Bevacizumab |
|  | R-05 | Male | 53 | T4 | No | Yes | Moderate | M2 | C | Sorafenib  Lenvatinib  Gefitinib  Regorafenib  Capecitabine  Oxaliplatin  Bevacizumab |
|  | R-06 | Male | 52 | T4 | Yes | Yes | Moderate | M2 | C | Lenvatinib  HAIC (Oxaliplatin + Fluorouracil) |
|  | R-07 | Male | 51 | T4 | Yes | Yes | Moderate-Poor | M2 | C | HAIC (Oxaliplatin + Fluorouracil)  Nivolumab  Lenvatinib |
|  | R-08 | Male | 60 | T3 | Yes | Yes | Moderate-High | M0 | B | HAIC (Oxaliplatin + Fluorouracil)  Sintilimab  Lenvatinib  Regorafenib |
|  | R-09 | Male | 58 | T2 | Yes | Yes | Moderate | M0 | B | TACE (Oxaliplatin + Fluorouracil)  Sintilimab  Lenvatinib |
|  | R-10 | Male | 48 | T2 | Yes | Yes | Poor | M0 | C | TACE (Oxaliplatin + Fluorouracil)  HAIC (Oxaliplatin + Fluorouracil)  Lenvatinib |
| DP | D-01 | Male | 45 | T3 | No | Yes | Poor | M2 | C | Sorafenib  Lenvatinib |
|  | D-02 | Male | 60 | T4 | Yes | Yes | Moderate-Poor | M2 | C | TACE (Oxaliplatin + Fluorouracil)  Lenvatinib  Pembrolizumab |
|  | D-03 | Female | 59 | T2 | Yes | Yes | Moderate | M1 | C | Paclitaxel  Cisplatin  Lenvatinib  Sintilimab |
|  | D-04 | Male | 52 | T4 | No | Yes | Moderate | M2 | C | Lenvatinib |
|  | D-05 | Male | 64 | T4 | Yes | Yes | Poor | M2 | C | HAIC (Oxaliplatin + Fluorouracil)  Lenvatinib  Tislelizumab |
|  | D-06 | Male | 48 | T4 | Yes | Yes | Poor | M2 | C | HAIC (Oxaliplatin + Fluorouracil)  Lenvatinib  Pembrolizumab |
|  | D-07 | Male | 35 | T4 | Yes | Yes | Moderate | M2 | C | HAIC (Oxaliplatin + Fluorouracil)  Lenvatinib  Sintilimab |

**Abbreviation:** PR: Partial Response; DP: Disease Progression; MVI: Microvascular Invasion; TACE: Transarterial Chemoembolization; HAIC: Hepatic Arterial Infusion Chemotherapy.

**Notes:** MVI-M0: Indicates that no microvascular invasion was found in the sections of the tumor tissue. M1: Belongs to the low-risk group, referring to the presence of no more than 5 microvascular invasions in all tissue sections, and these invasions all occur in the peri-tumoral liver tissue area (<1 cm of the tumor). M2: Belongs to the high-risk group, referring to the presence of more than 5 microvascular invasions in the sections of the tumor tissue, or microvascular invasions occurring in the distant peri-tumoral liver tissue area (>1 cm away from the tumor). Child-Pugh grade A: Indicates well-compensated cirrhosis with relatively preserved liver function. Patients in this category have a better prognosis and are often considered for surgical procedures or liver resection. Grade B: Suggests moderately decompensated liver disease. The prognosis is intermediate, and these patients may require more cautious management and are generally not considered ideal candidates for major liver resections. Grade C: Represents severe liver dysfunction with a poor prognosis. Patients with Child-Pugh C cirrhosis are at high risk of complications and are usually prioritized for liver transplantation rather than undergoing other invasive procedures.

## Supplementary Table 2. Clinical and pathological information of HCC patients with or without application of systemic therapy.

| Group | No. | Gender | Age (year) | T stage | HBsAg | Cirrhosis | Differentiation Grade | MVI | Systemic Therapy Drugs |
| --- | --- | --- | --- | --- | --- | --- | --- | --- | --- |
| Drug (-) | 0-01 | Male | 60 | T3 | No | Yes | Moderate | M0 | - |
|  | 0-02 | Male | 69 | T1b | Yes | Yes | Moderate | M1 | - |
|  | 0-03 | Female | 67 | Tx | Yes | Yes | Unknown | M2 | - |
|  | 0-04 | Female | 13 | T2 | No | Yes | Moderate | M2 | - |
|  | 0-05 | Male | 40 | T4 | Yes | Yes | Moderate | M2 | - |
|  | 0-06 | Male | 58 | T2 | Yes | Yes | Moderate | M1 | - |
|  | 0-07 | Male | 43 | T1a | Yes | Yes | Moderate | M0 | - |
|  | 0-08 | Male | 39 | T1b | Yes | Yes | Moderate | M0 | - |
|  | 0-09 | Male | 47 | Tx | Yes | Yes | Unknown | M0 | - |
|  | 0-10 | Male | 66 | T1b | Yes | Yes | Moderate-Poor | M0 | - |
|  | 0-11 | Male | 50 | T2 | Yes | Yes | Moderate | M0 | - |
|  | 0-12 | Male | 62 | T1b | No | Yes | Moderate | M0 | - |
|  | 0-13 | Male | 30 | T1b | Yes | Yes | Moderate | M0 | - |
|  | 0-14 | Male | 60 | T1b | No | Yes | Moderate | M1 | - |
|  | 0-15 | Male | 46 | T1a | Yes | Yes | Moderate | M0 | - |
|  | 0-16 | Male | 49 | t1b | Yes | Yes | Moderate | M0 | - |
|  | 0-17 | Male | 62 | T1a | Yes | Yes | Moderate | M0 | - |
|  | 0-18 | Male | 54 | T1a | Yes | Yes | Moderate | M0 | - |
|  | 0-19 | Male | 57 | T1a | Yes | Yes | Moderate | M0 | - |
|  | 0-20 | Male | 51 | T1a | Yes | Yes | High | M0 | - |
|  | 0-21 | Female | 84 | T1b | No | Yes | Moderate | M0 | - |
|  | 0-22 | Male | 66 | T1b | No | Yes | High-Moderate | M0 | - |
|  | 0-23 | Male | 49 | T1b | Yes | Yes | Moderate-Poor | M0 | - |
|  | 0-24 | Male | 55 | Tx | Yes | Yes | Unknown | Unknown | - |
| Drug (+) | 1-01 | Male | 68 | T1b | No | Yes | Moderate | M0 | Irinotecan  TACE (Oxaliplatin + Fluorouracil) |
|  | 1-02 | Male | 75 | T4 | No | Yes | Moderate | M2 | HAIC (Oxaliplatin + Fluorouracil) |
|  | 1-03 | Male | 62 | Tx | Yes | Yes | Moderate | M0 | TACE (Oxaliplatin + Fluorouracil) |
|  | 1-04 | Male | 69 | T4 | No | Yes | Moderate | M2 | XELOX (Oxaliplatin+Capecitabine) |
|  | 1-05 | Male | 47 | Tx | Yes | Yes | Unknown | M2 | Lenvatinib  Sintilimab |
|  | 1-06 | Female | 79 | Tx | HCV-cAg（+） | Yes | Unknown | M2 | TACE (Oxaliplatin + Fluorouracil)  Lenvatinib  Sintilimab |
|  | 1-07 | Male | 56 | Tx | Yes | Yes | Unknown | M0 | TACE (Oxaliplatin + Fluorouracil)  Lenvatinib  Sintilimab |
|  | 1-08 | Male | 54 | T4 | Yes | Yes | Poor | M2 | Sorafenib  Lenvatinib  Tislelizumab |
|  | 1-09 | Female | 47 | Tx | Yes | Yes | Unknown | Unknown | Lenvatinib  Tislelizumab  HAIC (Oxaliplatin + Fluorouracil)  Sorafenib  Sintilimab |
|  | 1-10 | Male | 57 | Tx | Yes | Yes | High | M0 | TACE (Oxaliplatin + Fluorouracil) |
|  | 1-11 | Male | 60 | T1b | Yes | Yes | High-Moderate | M0 | TACE (Oxaliplatin + Fluorouracil) |
|  | 1-12 | Male | 45 | T3 | Yes | Yes | Moderate-Poor | M0 | Lenvatinib  Camrelizumab |
|  | 1-13 | Male | 60 | Tx | No | Yes | Moderate | M0 | XELOX (Oxaliplatin+Capecitabine)  FOLFOX (Oxaliplatin + Fluorouracil)  Cetuximab  Irinotecan  Fluorouracil  Oxaliplatin  Bevacizumab  Camrelizumab |
|  | 1-14 | Male | 50 | T2 | Yes | Yes | Moderate | M0 | TACE (Oxaliplatin + Fluorouracil) |
|  | 1-15 | Male | 54 | Tx | Yes | Yes | Moderate | M2 | Lenvatinib  Pembrolizumab  TACE (Oxaliplatin + Fluorouracil) |
|  | 1-16 | Female | 60 | Tx | Yes | Yes | Moderate-Poor | M2 | Comprehensive Treatment |
|  | 1-17 | Male | 59 | Tx | Yes | Yes | Unknown | Unknown | TACE (Oxaliplatin + Fluorouracil)  Lenvatinib |
|  | 1-18 | Male | 73 | Tx | Yes | Yes | High | M0 | Comprehensive Treatment |
|  | 1-19 | Male | 54 | Tx | Yes | Yes | Moderate | M0 | HAIC (Oxaliplatin + Fluorouracil)  Lenvatinib |
|  | 1-20 | Male | 61 | T4 | Yes | Yes | Moderate | M2 | TACE (Oxaliplatin + Fluorouracil)  Lenvatinib  Tislelizumab |
|  | 1-21 | Male | 49 | Tx | Yes | Yes | High | M0 | Comprehensive Treatment |
|  | 1-22 | Male | 54 | T2 | Yes | Yes | Moderate | M0 | TACE (Oxaliplatin + Fluorouracil)  Lenvatinib  Sintilimab  Sorafenib |
|  | 1-23 | Female | 67 | Tx | No | Yes | Moderate | M0 | Comprehensive Treatment |
|  | 1-24 | Male | 60 | Tx | Yes | Yes | Unknown | Unknown | HAIC (Oxaliplatin + Fluorouracil)  Lenvatinib  Sintilimab  Sorafenib |
|  | 1-25 | Male | 58 | T3 | No | Yes | Moderate | M2 | Lenvatinib |
|  | 1-26 | Male | 48 | T4 | No | Yes | Moderate | M2 | FOLFOX (Oxaliplatin + Fluorouracil)  Pembrolizumab  CTLA-4  Ipilimumab |
|  | 1-27 | Male | 60 | Tx | Yes | Yes | High-Moderate | M0 | Comprehensive Treatment |

**Abbreviation:** MVI: Microvascular Invasion; TACE: Transarterial Chemoembolization; HAIC: Hepatic Arterial Infusion Chemotherapy; CTLA-4: Cytotoxic T-Lymphocyte-Associated Protein 4.

**Notes:** MVI-M0: Indicates that no microvascular invasion was found in the sections of the tumor tissue. M1: Belongs to the low-risk group, referring to the presence of no more than 5 microvascular invasions in all tissue sections, and these invasions all occur in the peri-tumoral liver tissue area (<1 cm of the tumor). M2: Belongs to the high-risk group, referring to the presence of more than 5 microvascular invasions in the sections of the tumor tissue, or microvascular invasions occurring in the distant peri-tumoral liver tissue area (>1 cm away from the tumor).

**Supplementary Table 3.** IC_50_ values of drug-resistant cells with AKR1B1 knockdown after treatment with drugs.

| Drugs (μM) | Huh-7 LR | | |  | Huh-7 SR |  |
| --- | --- | --- | --- | --- | --- | --- |
|  | Blank | sh-NC | sh-AKR1B1 | Blank | sh-NC | sh-AKR1B1 |
| Lenvatinib | 50.61±4.62 | 53.90±1.06 | 21.44±0.92 | 33.52±2.10 | 40.46±1.129 | 16.94±1.11 |
| Sorafenib | 21.06±0.66 | 21.09±1.04 | 8.03±0.97 | 16.70±1.15 | 14.57±1.04 | 8.31±0.99 |
| Regorafenib | 8.53±0.59 | 6.86±0.79 | 5.14±0.70 | 10.31±0.40 | 10.64±0.73 | 8.49±0.65 |
| Gefitinib | 25.37±2.70 | 24.37±0.80 | 16.13±0.84 | 22.43±1.37 | 21.18±0.85 | 18.14±0.96 |
| Lapatinib | 18.78±0.75 | 19.13±0.83 | 15.29±0.96 | 25.47±1.15 | 24.58±0.96 | 18.24±0.88 |
| 5-Fluorouracil | 42.74±3.58 | 61.22±0.98 | 38.90±1.67 | 37.31±2.61 | 36.11±0.70 | 33.02±1.18 |
| Irinotecan | 22.00±1.43 | 27.72±1.05 | 20.38±1.50 | 40.76±3.72 | 42.24±1.37 | 25.12±1.14 |
| Oxaliplatin | 59.25±4.59 | 55.05+0.92 | 42.66±0.88 | 79.75±4.39 | 72.38±5.22 | 58.80±3.61 |

**Supplementary Table 4.** IC_50_ values of drug-resistant cells with AKR1B1 overexpression after treatment with drugs.

| Drugs (μM) | Huh-7 P | | |
| --- | --- | --- | --- |
|  | Blank | oe-NC | oe-AKR1B1 |
| Lenvatinib | 13.02±0.73 | 13.15±0.99 | 22.61±1.05 |
| Sorafenib | 6.78±0.13 | 7.02±1.01 | 10.85±0.98 |
| Regorafenib | 6.40±0.24 | 6.042±0.86 | 7.53±0.90 |
| Gefitinib | 13.78±0.55 | 16.22±0.96 | 22.90±0.90 |
| Lapatinib | 14.13±0.51 | 14.23±0.94 | 15.88±0.95 |
| 5-Fluorouracil | 25.19±1.64 | 30.20±2.02 | 45.71±1.83 |
| Irinotecan | 24.54±1.44 | 28.85±1.21 | 23.28±1.05 |
| Oxaliplatin | 39.98±1.60 | 41.69±2.41 | 65.90±1.22 |

**Supplementary Table 5.** IC_50_ values for drug resistance transfer mediated by conditional medium.

| Cells & Drugs (μM) | CM from Huh-7 P | CM from Huh-7 LR | CM from Huh-7 SR |
| --- | --- | --- | --- |
| Huh-7 P (Lenvatinib treatment) | 18.79±0.98 | 24.91±1.07 | - |
| Huh-7 P (Sorafenib treatment) | 8.12±1.02 | - | 12.40±1.14 |

## Supplementary Table 6. The List of gene intervention used in this study.

| Gene intervention | Base information | | Company |
| --- | --- | --- | --- |
| sh-AKR1B1 | accession: NM_001628, target sequence: TGCTGAGAACTTTAAGGTCTT | | Genechem Co. |
| oe-AKR1B1 | accession: NM_001628, target sequence: AGGTCGACTCTAGAGGATCCCGCCACCATGGCAAGCCGTCTCCTGCTC | | Genechem Co. |
|  | sense 5'-3' | antisense 5'-3' |  |
| si-AKR1B1 | GGAUGAAGGGCUGGUGAAATT | UUUCACCAGCCCUUCAUCCTT | GenePharma Co. |
| si-SORD | GCCUGGAGAACUAUCCUAUTT | AUAGGAUAGUUCUCCAGGCTT | GenePharma Co. |
| si-KHK | GGUGGACAAGUACCCUAAGTT | CUUAGGGUACUUGUCCACCTT | GenePharma Co. |
| si-ALDOB | GGGAAUCAAGUUAGACCAATT | UUGGUCUAACUUGAUUCCCTT | GenePharma Co. |
| si-AGPAT4 | GCUAAUCAUCAACACCAUUTT | AAUGGUGUUGAUGAUUAGCTT | GenePharma Co. |
| si-DGAT1 | CCCGGUUAUUUCUGGAGAATT | UUGUAGAAGUGUCUGAUGCTT | GenePharma Co. |

Notes: sh-: short hairpin; oe-: overexpression; si-: small interfering; SORD: Sorbitol dehydrogenase; KHK: Ketohexokinase; ALDOB: Aldolase fructose-bisphosphate B; AGPAT4: 1-acylglycerol-3-phosphate O-acyltransferase 4; DGAT1: Diacylglycerol O-acyltransferase 1.

## Supplementary Table 7. The List of primary antibodies used in this study.

| Antibodies | Company | Catalog No. |
| --- | --- | --- |
| GLUT1 | Proteintech | 21829-1-AP |
| FABPs | Abcam | ab222517 |
| CD31 | Proteintech | 66065-2-Ig |
| FASN | Abclonal | A21182 |
| CD36 | Abclonal | A5792 |
| AFP | Abcam | ab169552 |
| Ki67 | Abcam | ab15580 |
| AKR1B1 | Abcam | ab153897 |
| CK19 | Abcam | ab52625 |
| EPCAM | Abcam | ab223582 |
| CD45 | Abcam | ab40763 |
| CK18 | Abcam | ab133263 |
| GPC3 | Abcam | ab207080 |
| GS | Proteintech | 11037-2-AP |
| CD34 | Abcam | ab81289 |
| β-actin | Servicebio | GB11001-100 |
| p-STAT3-Y705 | Abclonal | AP0070 |
| STAT3 | Abclonal | A1192 |
| SLC7A11 | Abclonal | A2413 |
| CD81 | Abclonal  Proteintech | A4863  66866-1-Ig |
| CD63 | Abclonal | A5271 |
| β-catenin | Proteintech | 51067-2-AP |
| Non-phospho (active) β-catenin | Cell Signaling Technology | 19807T |

## Supplementary Table 8. The List of fluorescence probes used in this study.

| Probes | Commercial name | Catalog No. | Ex/Em (nm) | Company |
| --- | --- | --- | --- | --- |
| Calcein AM | LIVE/DEAD™ Viability /Cytotoxicity Kit | L3224 | 495 /515 | ThermoFisher Scientific |
| EthD-1 | LIVE/DEAD™ Viability /Cytotoxicity Kit | L3224 | 528/617 | ThermoFisher Scientific |
| BODIPY | BODIPY™ 500/510 C1, C12 | D3823 | 500/510 | ThermoFisher Scientific |
| CellROX | CellROX™ Deep Red | C10422 | 644/665 | ThermoFisher Scientific |
| mBCI | Monochlorobimane | M1381MP | 394/490 | ThermoFisher Scientific |
| CellMask | CellMask™ Deep Red plasma membrane stain | C10046 | 649/666 | ThermoFisher Scientific |
| Nile Red | Nile Red | N1142 | 552/636 | ThermoFisher Scientific |
| Hoechst | Hoechst 33342 | H1399 | 352/461 | ThermoFisher Scientific |
| DAPI | DAPI | 62248 | 360/460 | ThermoFisher Scientific |
| CFDA SE | LumiTrace CFDA SE celltracing | 16231 | 492/517 | Lumiprobe |
| CellTracker^тм^ CM-Dil | CellTracker^тм^ CM-Dil | C7000 | 553/570 | ThermoFisher Scientific |

## Supplementary Table 9. The List of assay kits used in this study.

| Kit name | Catalog No. | Producer |
| --- | --- | --- |
| Cell-Counting-Kit-8 | C0038 | Beyotime Biotechnology |
| LIVE/DEAD™ Viability/Cytotoxicity Kit | L3224 | Invitrogen |
| Resazurin assay kit | R7017 | Sigma |
| VECTASTAIN® Elite^®^ ABC-HRP Kit | PK-6200 | Vector |
| Avidin/Biotin Blocking Kit | SP-2001 | Vector |
| Vector NovaRED^®^ Substrate Kit | SK-4800 | Vector |
| Sorbitol Content Detection Kit | G0560W | Grace Biotechnology |
| Cysteine Content Detection Kit | A126-1-1 | Nanjing Jiancheng Bioengineering |
| Coenzyme I (NAD^+^+NADH) Content Colorimetric Assay Kit | A114-1-1 | Nanjing Jiancheng Bioengineering |
| Coenzyme Ⅱ (NADP^+^+NADPH) Content Colorimetric Assay Kit | A115-1-1 | Nanjing Jiancheng Bioengineering |
| Triglyceride Single Reagent GPO-PAP Method Assay Kit | A110-1-1 | Nanjing Jiancheng Bioengineering |
| Omni-EasyTM Ready-to-Use BCA Protein Quantification Assay Kit | ZJ102 | Epizyme Biotech. |
| Cell Mitochondria Isolation Kit | C3601 | Beyotime Biotechnology |
| GENMED® Fatty Acid β-Oxidation Rate Colorimetric Assay Kit | GMS50679 | Genmed B.V. |
| Human AKR1B1 ELISA kit | EH2164 | FineTest |

## Supplementary Data List.

**Supplementary Data 1.** FPKM and count values of RNA_seq from parental cells and drug-resistant cells.

**Supplementary Data 2.** The list of 2906 upregulated genes in Huh-7 LR compared to parental cells.

**Supplementary Data 3.** The list of 1965 upregulated genes in Huh-7 SR compared to parental cells.

**Supplementary Data 4.** Huh-7 LR & SR co-upregulated gene KEGG pathway analysis Compared to the parental cells.

**Supplementary Data 5.** The downstream genes of transcription factors in the "On" state of Huh-7 LR undergo PPI analysis compared to the parental cells.

**Supplementary Data 6.** The downstream genes of transcription factors in the "On" state of Huh-7 P undergo PPI analysis compared to Huh-7 LR.

**Supplementary Data 7.** The proportion of pathways in each GEO dataset.

**Supplementary Data 8.** Metabolomics analysis reveals altered metabolic pathways in drug-resistant cells & parental cells.

**Supplementary Data 9.** FFA detection results in drug-resistant cells & parental cells.

**Supplementary Data 10.** KEGG enrichment analysis of upregulated metabolites in Huh-7 LR compared to the parental cells.

**Supplementary Data 11.** Real-time tracking of fatty acid uptake in drug-resistant cells and parental cells. FFA (green), Nucleus (blue). Scale bar = 50 μm. Tracking duration: 7 h 15 min, Time interval: 15 min.

**Supplementary Data 12.** Metabolic flux detection results in drug-resistant cells compared to the parental cells.

**Supplementary Data 13.** Metabolic pathways associated with genes that are overexpressed in the Huh-7 LR compared to the parental cells.

**Supplementary Data 14.** Real-time tracking of extracellular vesicles transfer from the drug-resistant cell to the parental cell. Huh-7 LR tracking probe (green), Huh-7 P tracking probe (orange). Scale bar = 10 μm. Tracking duration: 4 h 30 min, Time interval: 30 min.

**Supplementary Data 15.** Real-time tracking of the effects of combined drug therapy in HCC PDOs. Scale bar = 500 μm. Tracking duration: 48 h, Time interval: 2 h.

**References**

1. Li, C. *et al.* cFUT8 promotes liver cancer progression by miR-548c/FUT8 axis. *Signal Transduct Target Ther* **6**, 30 (2021).

2. Li, Q. *et al.* HIF-1alpha-induced expression of m6A reader YTHDF1 drives hypoxia-induced autophagy and malignancy of hepatocellular carcinoma by promoting ATG2A and ATG14 translation. *Signal Transduct Target Ther* **6**, 76 (2021).

3. Shan, Q. *et al.* The p-MYH9/USP22/HIF-1alpha axis promotes lenvatinib resistance and cancer stemness in hepatocellular carcinoma. *Signal Transduct Target Ther* **9**, 249 (2024).

4. Zimmermann, M., Sauer, U. & Zamboni, N. Quantification and mass isotopomer profiling of alpha-keto acids in central carbon metabolism. *Anal Chem* **86**, 3232-3237 (2014).

5. Song, J.W. *et al.* Omics-Driven Systems Interrogation of Metabolic Dysregulation in COVID-19 Pathogenesis. *Cell Metab* **32**, 188-202 e185 (2020).

6. Tian, H. *et al.* Precise Metabolomics Reveals a Diversity of Aging-Associated Metabolic Features. *Small Methods*, e2200130 (2022).

7. Lam, S.M. *et al.* Quantitative Lipidomics and Spatial MS-Imaging Uncovered Neurological and Systemic Lipid Metabolic Pathways Underlying Troglomorphic Adaptations in Cave-Dwelling Fish. *Mol Biol Evol* **39** (2022).

8. Lam, S.M. *et al.* A multi-omics investigation of the composition and function of extracellular vesicles along the temporal trajectory of COVID-19. *Nat Metab* **3**, 909-922 (2021).

9. Shen, L. *et al.* Serine metabolism antagonizes antiviral innate immunity by preventing ATP6V0d2-mediated YAP lysosomal degradation. *Cell Metab* **33**, 971-987 e976 (2021).

10. Jiang, Y. *et al.* Proteomics identifies new therapeutic targets of early-stage hepatocellular carcinoma. *Nature* **567**, 257-261 (2019).
