# Supplementary figures and images for: Targeting AKR1B1 inhibits metabolic reprogramming to reverse systemic therapy resistance in hepatocellular carcinoma
Source: Signal Transduct Target Ther. 2025 Aug 1;10:244. doi: 10.1038/s41392-025-02321-9 (PMC12317016; doi:10.1038/s41392-025-02321-9)

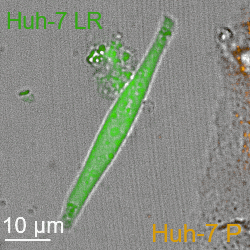

Supplement: Supplementary file 15 — Supplementary Data 14 [file 41392_2025_2321_MOESM15_ESM.gif]
